# Fungal metabolite Ochratoxin A inhibits MrkD1P of multidrug-resistant *Klebsiella pneumoniae*: Integrated computational and in vitro validation

**DOI:** 10.1007/s10822-025-00661-w

**Published:** 2025-09-16

**Authors:** Md Roqunuzzaman, Ariful Islam, Sumaiya Jahan Supti, Mahbub Hasan Rifat, Mohammad Saiful Islam, Ummay Habiba Ananna, Khalid Saifullah Tusher, Aamal A. Al-Mutairi, Magdi E. A. Zaki, Subir Sarker, Md. Eram Hosen

**Affiliations:** 1 Department of Genetic Engineering and Biotechnology, University of Rajshahi, Rajshahi, Bangladesh; 2 Department of Chemistry, College of Science, Imam Mohammad Ibn Saud Islamic University (IMSIU), Riyadh, 11623 Kingdom of Saudi Arabia; 3 Biomedical Sciences and Molecular Biology, College of Medicine and Dentistry, James Cook University, Townsville, QLD 4811 Australia; 4 Department of Microbiology, Shaheed Shamsuzzoha Institute of Biosciences, Affiliated with University of Rajshahi, Rajshahi, Bangladesh

**Keywords:** Fungal metabolites, *Klebsiella pneumoniae*, Fimbrial adhesin protein MrkD1P, Molecular docking, Dynamics, PCA analysis, Antibacterial activity

## Abstract

Multidrug-resistant (MDR) *Klebsiella pneumoniae* poses a significant global health concern, particularly in hospital setting where it causes severe and hard-to-treat infections. In this study, 329 fungal-derived compounds were screened for their potential to inhibit MrkD1P, a key fimbrial adhesin protein (PDB ID: 3U4K) involved in host tissue adhesion. Molecular docking analysis identified ochratoxin A (− 9.1 kcal/mol), bromadiolone (− 8.6 kcal/mol), and permethrin (− 8.2 kcal/mol) as top-performing candidates, exhibiting strong binding affinities and stable molecular interactions, including hydrogen bonding and hydrophobic contacts. These findings were reinforced by 100-nanosecond molecular dynamics (MD) simulations, which showed sustained ligand–protein stability, particularly for ochratoxin A. Free energy estimations using the MM/PBSA method further suggested the thermodynamic favourability of these interactions. Pharmacokinetic profiling (ADMET) indicated favourable absorption and distribution properties for all three compounds, with low toxicity predictions, though some hepatotoxicity was noted. Principal component analysis (PCA) demonstrated that ochratoxin A and permethrin induced substantial alterations in protein dynamics, suggesting ligand-specific structural effects. Experimental validation confirmed the antibacterial activity of ochratoxin A against *K. pneumoniae*, producing a 34 ± 0.67 mm inhibition zone at 100 µg/disc, surpassing ciprofloxacin (33 mm) with a MIC of 18.33 ± 0.72 µg/mL and MBC of 39.33 ± 1.36 µg/mL (*p* < 0.05). Collectively, these in silico and in vitro results highlight fungal metabolites, particularly ochratoxin A, as promising therapeutic leads against MDR K. *pneumoniae*. However, further in vivo investigations are required to establish their safety and clinical potential.

**Graphical abstract:**

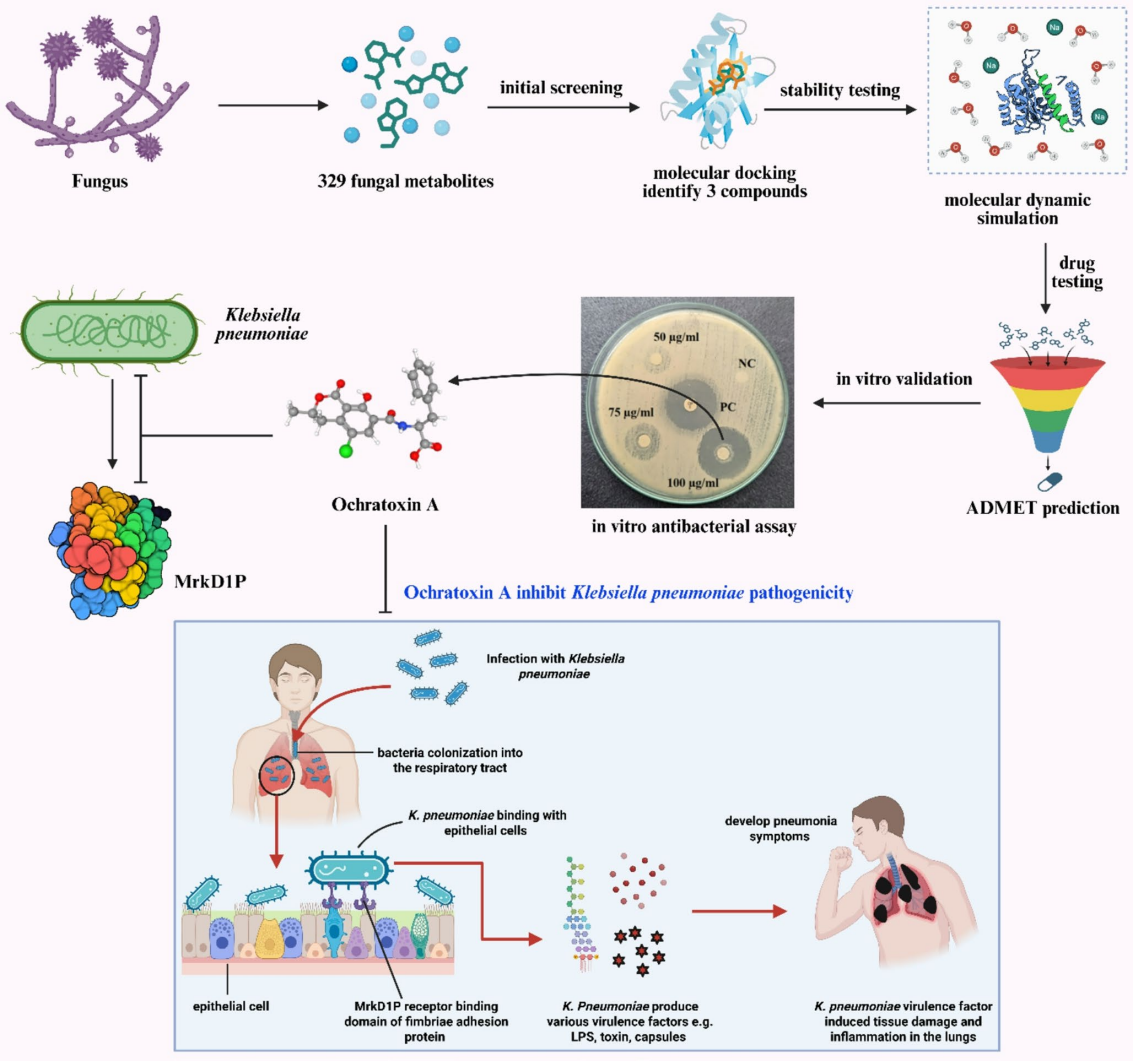

**Supplementary Information:**

The online version contains supplementary material available at 10.1007/s10822-025-00661-w.

## Introduction

Bacterial infections represent a significant global health concern, contributing substantially to morbidity, mortality, and increased healthcare costs. Among pathogenic bacteria, multidrug-resistant (MDR) strains pose an escalating threat, causing difficult-to-treat infections and prolonged hospitalizations [1]. The World Health Organization (WHO) estimates that infections caused by MDR pathogens could result to as many as 10 million deaths annually by 2050, underscoring the urgent need for novel therapeutic interventions [2]. *Klebsiella pneumoniae* is a prominent gram-negative bacterium belonging to the *Enterobacteriaceae* family, frequently implicated in both community-acquired and healthcare-associated infections [3]. This opportunistic pathogen is commonly associated with pneumonia, urinary tract infections, bloodstream infections, wound infections, and meningitis, particularly affecting immunocompromised patients, elderly individuals, and neonates [4]. *K. pneumoniae* is ubiquitously present in environmental reservoirs, including water and soil, and is also found animals, facilitating its persistence and wide dissemination across healthcare and communities settings [5].

The clinical management of *K. pneumoniae* infections is further complicated by the emergence of hypervirulent and antibiotic-resistant strains [6]. Carbapenem-resistant *K. pneumoniae* (CRKP), in particular, poses a critical global health concern due to resistance to nearly all available β-lactam antibiotics, including carbapenems, which are typically reserved as last-line therapeutic options [7]. Resistance arises through multiple mechanisms, including f carbapenemase production, modification of antibiotic targets, efflux pump overexpression, and reduced membrane permeability via porin alterations [8, 9]. CRKP strains frequently harbor plasmids encoding carbapenemases that hydrolyze carbapenems, conferring extensive drug resistance and leaving limiting treatment options [10, 11].

**Fig. 1:**
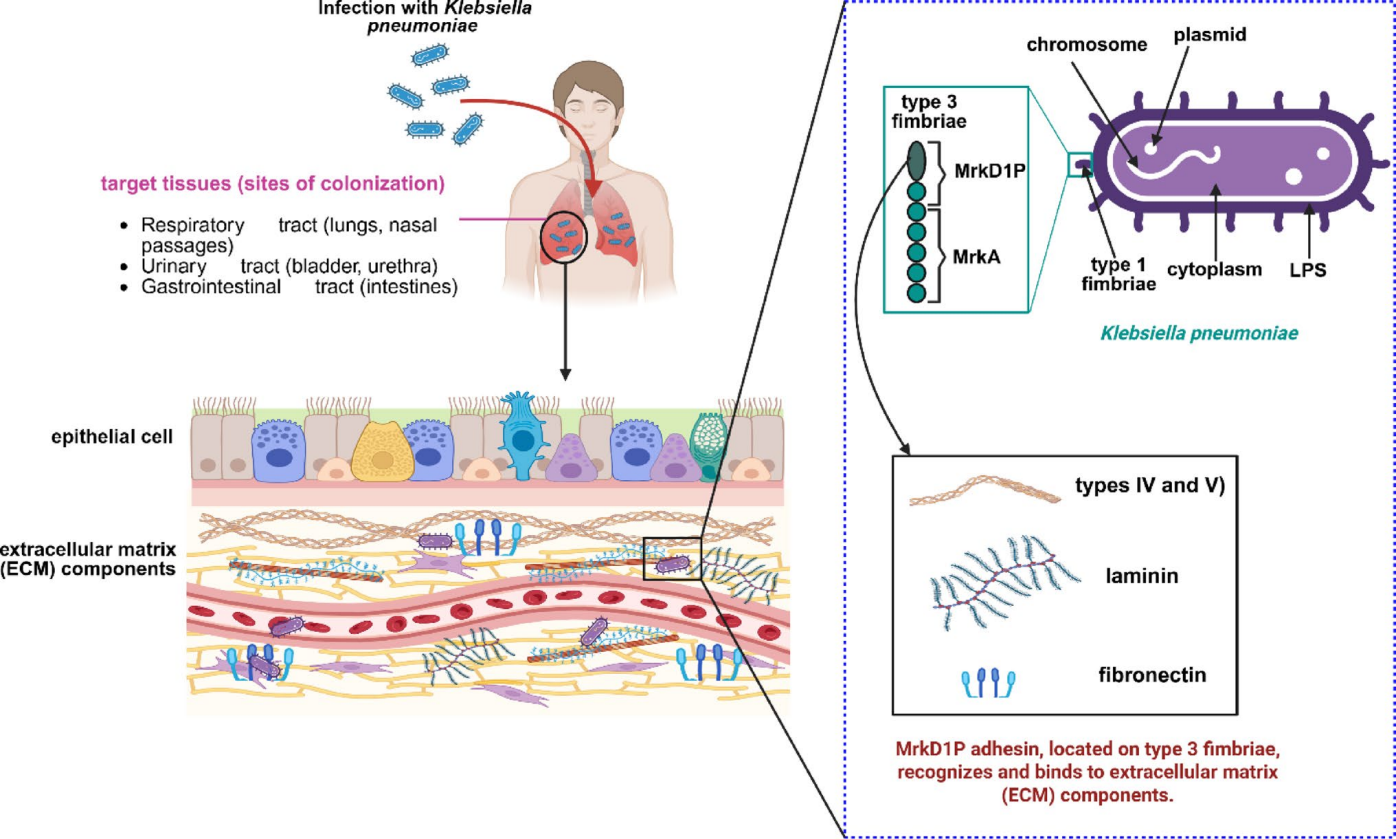
Mechanistic pathway of *K. pneumoniae* colonization and attachment. The bacterium colonizes the respiratory, urinary, and gastrointestinal tracts and adheres to host epithelial surfaces via type 1 and type 3 fimbriae. The MrkD1P adhesin, located on type 3 fimbriae, mediates specific binding to host extracellular matrix components, facilitating stable attachment. This pathway highlights MrkD1P as a critical molecular target for interventions aimed at preventing *K. pneumoniae* colonization and subsequent infection

A central factor in the pathogenicity of *K. pneumoniae* is its ability to adhere to host tissues, a prerequisite for colonization and infection establishment [12]. The pathogenic process follow a multistep process involving colonization of the respiratory tract, adhesion to epithelial surfaces, and the deployment of a suite of virulence determinants [13], including capsular polysaccharides, lipopolysaccharide (LPS), and secreted toxins, which collectively drive host tissue injury and inflammatory responses [14, 15]. Among these, fimbrial adhesins play a central role by mediating host cell adherence and biofilm formation, thereby facilitating persistent colonization including the respiratory, urinary, and gastrointestinal tracts. This process is primarily mediated by fimbrial structures, notably type 1 and type 3 fimbriae, which facilitate adhesion to host epithelial surfaces. Among these, the MrkD1P adhesin located on type 3 fimbriae plays a pivotal role by specifically binding to extracellular matrix components e.g. collagens (types IV and V), laminin, and fibronectin in the lungs and kidneys, promoting stable attachment. LPS further enhances adhesion, particularly to mucosal and abiotic surfaces, and collectively these interactions trigger the early stages of biofilm formation [16, 17] (Fig. 1). This distinct collagen-binding property makes MrkD1P functionally different from other fimbrial adhesins and positions it as a unique therapeutic target. Unlike resistance mechanisms such as β-lactamases or efflux pumps, adhesins represent an underexplored therapeutic avenue, as inhibiting adhesion can prevent bacterial attachment and infection at an early stage. Therefore, targeting MrkD1P is strategically significant because it represents a key determinant of tissue-specific colonization and biofilm initiation, making it a potential candidate for anti-adhesion therapies or drug development against *K. pneumoniae* infections. Structurally, MrkD1P exhibits a jelly-roll β-barrel fold, closely related to the F17-GafD family of receptor domains that specifically recognize N-acetyl-D-glucosamine (GlcNAc) residues [18]. This structural similarity underscores the importance of MrkD1P as a promising therapeutic target, as inhibition of this adhesin could effectively disrupt bacterial colonization and subsequent infection [19].

Given the growing prevalence of antibiotic resistance and the limited pipeline of new antibiotics, natural products, particularly fungal metabolites, are increasingly recognized as promising alternatives for antimicrobial drug discovery [20]. Fungal metabolites exhibit remarkable chemical diversity and biological activities, including antimicrobial, anticancer, and immunomodulatory properties [21–23]. These secondary metabolites often provide unique chemical scaffolds not found in synthetic compounds, making them ideal candidates to combat antibiotic resistance by targeting novel mechanisms and pathways in pathogenic bacteria [24, 25]. Given their remarkable structural diversity and bioactivity, fungal metabolites were considered promising candidates for targeting the MrkD1P adhesin. These natural products offer unique chemical scaffolds not typically found in synthetic libraries and have been reported to possess antimicrobial and anti-virulence activities, making them attractive for anti-adhesion–based strategies against *K. pneumoniae*. In recent decades, computational approaches such as in silico drug design have significantly advanced drug discovery efforts, particularly in the identification of novel antimicrobial agents [26]. Techniques including molecular docking, molecular dynamics (MD) simulations, virtual screening, and ADMET (Absorption, Distribution, Metabolism, Excretion, and Toxicity) profiling enable rapid and cost-effective evaluation of compound libraries. These methodologies facilitate the prediction of ligand-protein binding affinities, interaction stability, pharmacokinetics, and potential toxicities, streamlining the selection of viable drug candidates for subsequent experimental validation [27]. The integration of computational tools with in vitro experimentation represents a robust strategy for developing effective antimicrobials against MDR pathogens [28].

Therefore, the present study aims to investigate the inhibitory potential of fungal metabolites, specifically ochratoxin A, against the MrkD1P protein of MDR *K. pneumoniae*. By integrating comprehensive computational analyses, alongside experimental in vitro validation of antibacterial efficacy, this study provides crucial insights into the potential of fungal-derived metabolites as promising therapeutic leads. Such a multidisciplinary approaches hold significant promise in addressing the escalating challenge posed by antibiotic-resistant bacterial infections and improve clinical outcomes globally.

## Materials and methods

### Preparation of the target protein, Data collection of the target protein and its preparation

In the initial stage of the study, the target protein, Fimbria adhesin protein (MrkD1P) from *K. pneumoniae*, was selected for computational analysis following a thorough review of the available literature. The crystal structure of this protein (PDB ID: 3U4K) was retrieved from the Protein Data Bank (PDB) (https://www.rcsb.org/structure/3U4K). The structure was then subjected to a series of preparatory steps to ensure its suitability for molecular docking simulations. The protein was first cleaned by removing any bound heteroatoms, ligands, and water molecules using Discovery Studio 2024, a widely used molecular modeling tool, to eliminate extraneous elements that could interfere with the docking process (https://www.3ds.com/products/biovia/discovery-studio). Subsequently, the protein’s conformational energy was minimized to relieve steric clashes and optimize the structure using the SwissPDB Viewer software (v4.1) (https://spdbv.unil.ch/disclaim.html). The minimization process utilized the GROMOS96 43b1 force field, which is commonly employed for energy calculations and refinement of protein structures in computational studies [29]. To further ensure the accuracy of electrostatic interactions during docking, the protonation states of ionizable residues were checked and adjusted. These meticulous preparation steps were essential to prepare the protein for subsequent docking simulations and ensure high-quality docking results.

### Ligand identification and preparation

The selection of fungal metabolites was based on the following criteria: (i) documented occurrence in exo- and endophytic fungi, (ii) reported antimicrobial or anti-virulence activities in the literature, (iii) structural diversity to represent major mycotoxin classes, and (iv) commercial availability in analytical grade to ensure reproducibility of subsequent in vitro validation. This systematic approach ensured that the chosen ligands were biologically relevant, structurally diverse, and experimentally feasible. Based on these criteria, a comprehensive search of literature was performed, resulting in the identification of 329 potential ligands with the potential to modulate the activity of the target protein. The 3D molecular structures of these selected secondary metabolites were obtained in SDF (Structure Data File) format from the PubChem database [30]. To facilitate their use in docking simulations, the SDF files were converted into the PDBQT format using the Open Babel software [31], ensuring compatibility with the docking software.

### Binding site prediction

The active site of the Fimbria adhesin protein (3U4K) was identified using the CASTp server (Computer Atlas of Surface Topology of Proteins) (http://sts.bioe.uic.edu/castp/index.html?2r7g). CASTp is a computational tool designed to automatically locate and measure protein pockets and cavities using advanced geometric algorithms. This method is based on the precise application of alpha shapes and distinct flow theory to analyze protein structures. CASTp enables the detection of both solvent-accessible surface pockets and interior inaccessible cavities by locating, delineating, and quantifying hollow regions on the three-dimensional structure of the protein. The measurements are carried out using two models: the solvent-accessible surface model (Richards’ surface) and the molecular surface model (Connolly’s surface) [32].

### Molecular docking

Molecular docking was performed to assess the binding affinity of secondary metabolites (SMs) with the Fimbria adhesin protein of *Klebsiella pneumoniae*. The PyRx software (v0.8) was employed for docking analysis, utilizing the AutoDock Vina configuration with slight adjustments to enhance the accuracy of the results (https://sourceforge.net/projects/pyrx/). To prepare the input structures for docking, the protein structure was converted into a macromolecule format compatible with PyRx, and the ligands were imported in PDBQT format. The docking process required careful configuration of the grid box parameters, which define the region of the protein where the docking simulations occur. The center coordinates for the grid box were set to (3.8347 Å, -0.1554 Å, 8.7332 Å), and the dimensions of the grid box were defined as 48.7233 Å x 41.3090 Å x 59.7350 Å, respectively, ensuring that the active site of the protein was fully covered for docking analysis. The results were then refined by analyzing the docking interactions in Discovery Studio, which allowed for a more detailed examination of the molecular interactions and conformational changes in the protein-ligand complexes.

### Molecular dynamics simulation

Molecular dynamics (MD) simulations were conducted to evaluate the structural dynamics and stability of the protein–ligand complexes using YASARA Dynamics (v19.12.4) with the AMBER14 force field for reliable molecular interaction modeling [33]. System preparation included optimization of the hydrogen bonding network. Complexes were solvated in a TIP3P water box at 25 °C and 1 atm, maintaining a water density of 0.997 g/L [34]. Simulations mimicked physiological conditions, with 0.9% NaCl, a pH of 7.4, and a temperature of 310 K to ensure system neutrality [35]. Electrostatic interactions were treated using the Particle Mesh Ewald (PME) method with an 8.0 Å cutoff [36]. The integration timestep was set at 1.25 fs, and trajectory frames were recorded every 100 ps for a total simulation duration of 100 ns [37]. Post-simulation analyses included the computation of Root-Mean-Square Deviation (RMSD), Root-Mean-Square Fluctuation (RMSF), Radius of Gyration (Rg), Solvent Accessible Surface Area (SASA), and hydrogen bond interaction to assess conformational stability, structural compactness, molecular flexibility, and intermolecular binding characteristics over time.

### Binding-free energy estimation via MM/PBSA

To assess the binding affinity and stability of the protein–ligand complexes, the Molecular Mechanics Poisson–Boltzmann Surface Area (MM/PBSA) method was employed. This approach enables decomposition of binding free energy into molecular mechanical and solvation energy components, offering a comprehensive understanding of the energetic factors influencing complex formation [38, 39]. Calculations were performed using YASARA Dynamics (v19.12.4) with the AMBER14 force field, based on representative frames extracted from the molecular dynamic’s trajectories.

The binding free energy was determined using the following equation:

MM/PBSA binding free energy = E_potReceptor_ + E_solvReceptor_ + E_potLigand_ + E_solvLigand_ – E_potComplex_ − E_solvComplex_.

This formulation incorporates both the gas-phase potential energy and solvation energy of the isolated components and the bound complex. YASARA’s built-in MM/PBSA macros were utilized for efficient energy computation and automation of the analysis pipeline [40].

### Principal components analysis (PCA)

To investigate the conformational variability and dynamic behaviour of the protein–ligand complexes, PCA was performed. This multivariate statistical technique allowed for a comprehensive comparison across simulation systems, including the control protein structure and a known drug-bound reference complex. PCA facilitated the identification of dominant motion patterns and structural changes throughout the 100 ns MD simulations by reducing high-dimensional trajectory data into principal components [41, 42]. The analysis involved the construction and diagonalization of covariance matrices derived from atomic positional fluctuations, followed by the computation of eigenvalues and eigenvectors. Eigenvalues represented the magnitude of structural variance, while eigenvectors described the directionality of these movements. Prior to PCA, trajectory data were mean-centered and standardized to unit variance. The analysis was conducted using Python (v3.11) with the Scikit-learn (v1.2) library, and visualizations were generated using Matplotlib (v3.7) [43, 44].

### ADMET analysis

After completing molecular docking and molecular dynamics simulations, the shortlisted phytochemical compounds were subjected to pharmacokinetic and toxicity evaluation through ADMET (Absorption, Distribution, Metabolism, Excretion, and Toxicity) analysis. The PKCSM online platform [45] was employed to predict critical pharmacokinetic properties, including absorption potential, tissue distribution, metabolic stability, clearance, and toxicity risks. To further assess drug-likeness, the SwissADME tool was used, focusing on Lipinski’s Rule of Five- a set of criteria commonly used to estimate oral bioavailability [46]. This rule evaluates key molecular features such as molecular weight, LogP (lipophilicity), number of hydrogen bond donors and acceptors, and the count of rotatable bonds. Compliance with these criteria indicates the potential for favorable pharmacokinetic behavior and suitability for oral administration.

### In vitro antibacterial activity

#### Chemical and reagents

High-performance liquid chromatography (HPLC) grade standards of ochratoxin A was purchased from Sigma-Aldrich (USA). Methanol of HPLC-grade quality was used in sample preparation. Ciprofloxacin, used as a reference antibiotic, was obtained from Square Pharmaceuticals Ltd. Luria Bertani (LB) broth and LB agar media were also sourced from Sigma-Aldrich (USA) for microbial culture and growth experiments.

#### Bacterial sample selection and inventory

The *Klebsiella pneumoniae* bacteria sourced from the Molecular Biology and Protein Science Laboratory under the Department of Genetic Engineering and Biotechnology, University of Rajshahi, Bangladesh. For the purpose of colony formation of the desire bacteria, LB (Luria-Bertani) media was utilized. Afterwards, the plate had placed in incubator for inoculation at 37 °C for overnight. *K. pneumoniae* preserved at -80 °C for long-term preservation. According to NIH (National Institutes of Health), *K. pneumonia* grouped as Biosafety Level 2 (BSL-2) pathogen. Thus, we followed all applicable standards and regulations for correctly using and handling the microorganism. Therefore, all safety guidelines have followed to maintain and use the microorganism.

#### Determination of minimum inhibitory concentration (MIC)

The minimum inhibitory concentration (MIC) of ochratoxin A, identified as the most promising compound, was determined using the tube dilution method, following modified Clinical and Laboratory Standards Institute (CLSI) protocols [47, 48]. Serial dilutions were prepared from a 5 mg/mL stock solution in dimethyl sulfoxide (DMSO), yielding final concentrations ranging from 1 µg/mL to 20 µg/mL in sterile nutrient broth. For each test compound- ochratoxin A and the reference antibiotic ciprofloxacin—three independent sets of 20 sterile test tubes were prepared. Each tube received 1 mL of the respective diluted compound and 100 µL of an overnight bacterial culture. Tubes were incubated at 37 °C for 24 h. Ciprofloxacin-treated tubes containing bacterial inoculum served as the positive control, while the negative control consisted of bacteria grown in nutrient broth without any antimicrobial agent.

#### Determination of minimum bactericidal concentration (MBC)

The minimum bactericidal concentration (MBC) was defined as the lowest concentration of ochratoxin A, and ciprofloxacin that resulted in complete eradication of the test bacteria. MBC was determined by transferring 50 µL from the MIC test tubes showing no visible bacterial growth into 100 µL of fresh nutrient broth, followed by incubation at 37 °C for 48 h. The MBC was identified as the lowest concentration at which no bacterial growth was observed, indicating 100% bactericidal activity [49, 50].

#### Evaluation of in vitro antibacterial activity

Secondary metabolite compound with highest binding affinity that signifies the inhibitory activity against *K. pneumonia*. Therefore, this promising candidate named ochratoxin A was used in further investigation to understand its antibacterial activity towards *K. pneumonia*. To dissolve the compound, we used 60% methanol. For the experiment purpose, we had followed the disc diffusion assay [50]. The concentrations of ochratoxin A (50, 75, and 100 µg/disc) used in the disc diffusion assay were selected based on preliminary optimization experiments. Initial MIC and MBC determinations against *K. pneumoniae* were performed, yielding values of 18.33 µg/disc and 39.3 µg/disc, respectively. The concentrations chosen for the assay were therefore above the inhibitory threshold to allow clear observation of dose-dependent antibacterial effects while ensuring reproducible and reliable results. This approach ensured that the selected doses were biologically relevant and suitable for evaluating the antibacterial activity of ochratoxin A. The inoculum of the bacteria cultured through overnight shaking at 37 °C and 180 rpm in the LB nutrient broth. Nonetheless, the amount of bacterial suspension used to follow spread-plate technique for plate inoculation on LB agar plate was 1 × 10^6^ CFU/ml. Filter paper discs (Whatman No.1) with a diameter of 5 mm were used. Ochratoxin A integrated in the concentration of 50, 75, and 100 µg/disc, and the evolved discs were prepared inside the biosafety cabinet level 2. The standard antibiotic ciprofloxacin was used as a positive control. It was selected due to its broad-spectrum activity against Gram-negative pathogens, established clinical use in treating *K. pneumoniae*, and inclusion in standard antimicrobial susceptibility testing panels (CLSI and EUCAST) with well-defined MIC and disk-diffusion breakpoints [51–53]. While last-resort antibiotics such as carbapenems or colistin are highly potent, they are generally reserved for severe multidrug-resistant cases and are less suitable as routine comparators; moreover, colistin susceptibility cannot be reliably assessed by disk diffusion [54]. Therefore, ciprofloxacin provides a standardized and widely recognized benchmark for evaluating antibacterial efficacy in this study. The agar plate was incubated overnight (16 h) to prevent excessive growth, and then growth inhibition zones measured around discs. The obtained number of the inhibition zone recorded with a scale of millimeters (mm). To ensure precision, the experiment was repeated three times, and the data were subsequently presented as the mean and standard deviation of the results.

#### Statistical analysis

All data are presented as mean ± standard error (SE). Data processing was carried out using Microsoft Excel 2016. Statistical differences between the means of three replicates were evaluated using one-way ANOVA followed by Duncan’s multiple range test, with significance set at *p* < 0.05. These analyses were performed using SAS software (version 9.1.3).

## Results and discussions

### Molecular docking

**Fig. 2:**
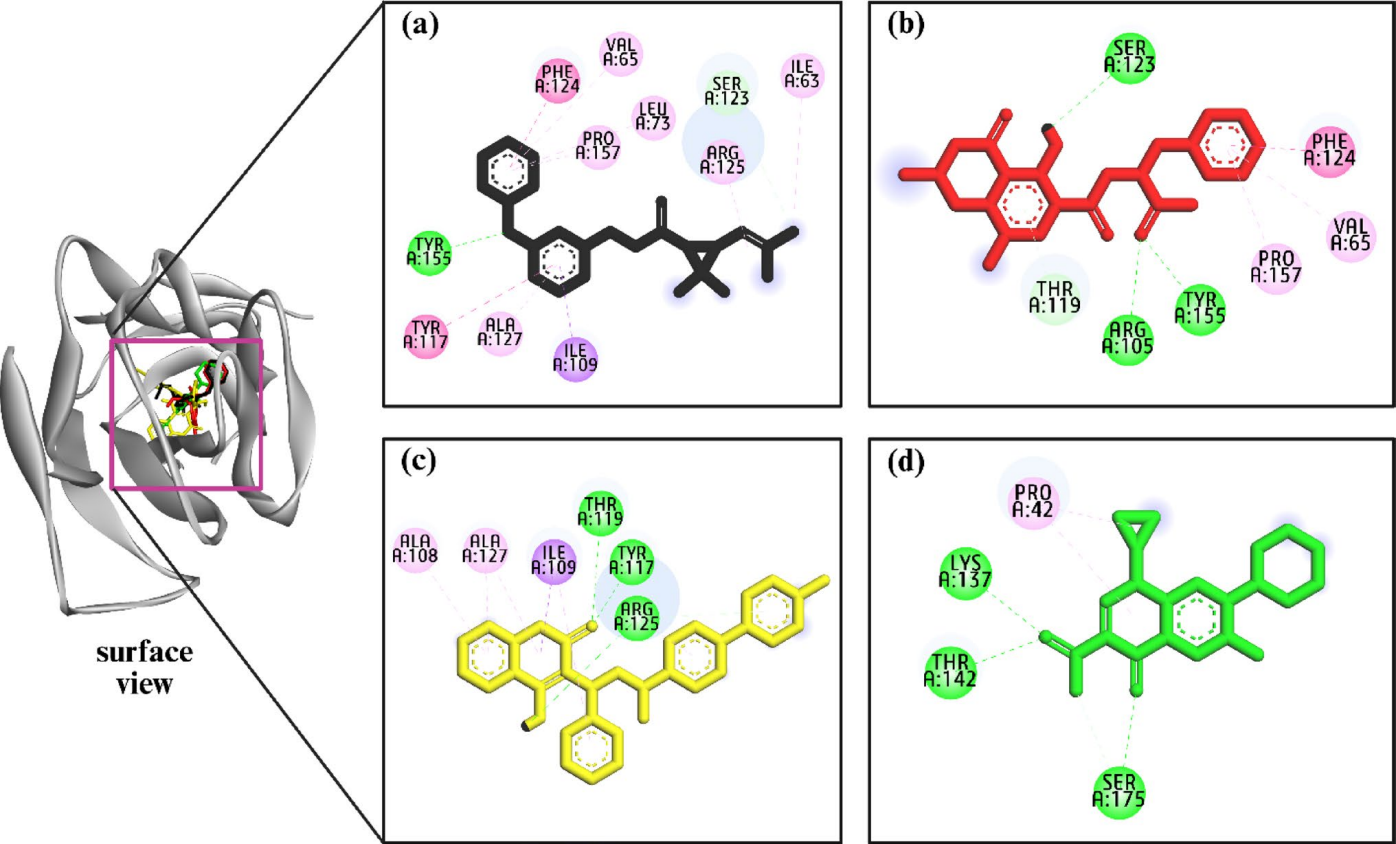
Molecular docking interactions of the compound **a** permethrin, **b** ochratoxin A, **c** bromadiolone, and **d** control ciprofloxacin with 3U4K protein of *K. pneumoniae*; surface and 2D view of compounds. Where, surface view depicts all four compounds bind to the almost same surface and active site of the protein

Molecular docking of 329 anti-pneumonia compounds against the Fimbria adhesin protein (PDB ID: 3U4K) of *K. pneumonia* revealed three lead candidates with the highest binding affinities [55], providing a foundation for rational design and optimization of improved therapeutic agents (Supplementary Table S1). Ochratoxin A showed the strongest binding energy (− 9.1 kcal/mol) and formed four strong hydrogen bonds with active-site residues ARG105*, TYR155*, SER123, and THR119*, with bond distances ranging from 2.14 to 2.57 Å, indicating high binding specificity and stability. Bromadiolone followed with a binding energy of − 8.6 kcal/mol and three hydrogen bonds with TYR117*, THR119*, and ARG125* (1.79–2.38 Å), suggesting a tightly bound complex. Permethrin, with a binding energy of − 8.2 kcal/mol, formed one hydrogen bond with TYR155* (4.42 Å) and a carbon-hydrogen bond with SER123, alongside several hydrophobic and π-interactions involving active residues like TYR117*, PRO157*, PHE124*, and ILE109*, reflecting broader engagement across the binding pocket. In contrast, the control compound exhibited the weakest binding (− 6.8 kcal/mol) and formed only three hydrogen bonds with LYS137*, THR142*, and SER175*, with longer bond distances (1.09–3.25 Å), and limited active-site interaction. Recent studies suggest compounds that target key *K. pneumoniae* proteins, namely blaNDM-1 or β-lactamases, often exhibit docking scores of -6 to -8 kcal/mol, which signifies moderate to strong binding yet lower than that of OTA’s in the current investigation [56, 57]. Moreover, prior research with equivalent molecular docking studies has highlighted the therapeutic potential through targeting bacterial adhesins named MrkD1P to disrupt the pathogenic adhesion process and biofilm development (e.g., targeting MrkD1P with benzoic acid derivatives) [58]. Collectively, the results identify ochratoxin A as the most promising inhibitor due to its strong affinity and extensive hydrogen bonding with active-site residues [59, 60], followed by bromadiolone and permethrin, both of which also demonstrate potential for further optimization (Fig. 2 and Table 1).

The Fimbria adhesin protein of *K. pneumoniae* (PDB ID: 3U4K), particularly the MrkD1P domain, remains largely uncharacterized as a therapeutic target for fungal-derived metabolites. Our molecular docking investigation suggests that selected compounds exhibit favourable interactions within potential binding pockets of the 3U4K protein, indicating their potential to act as inhibitors of the adhesin-mediated colonization process. Among the three compounds evaluated, permethrin, although primarily known as a synthetic pyrethroid insecticide, has shown limited antibacterial activity at high concentrations presumably through disruption of bacterial membranes, including against methicillin-resistant *Staphylococcus aureus* [61]. However, its clinical relevance as an antimicrobial agent remains minimal. Bromadiolone, an anticoagulant rodenticide, lacks any documented bactericidal or inhibitory effects against *K. pneumoniae*, and no peer-reviewed literature supports its antimicrobial use.

In contrast, ochratoxin A, a mycotoxin biosynthesized by *Aspergillus* and *Penicillium* species and commonly encountered as a food contaminant in cereals, coffee, and dried fruits has recently garnered interest for its antimicrobial potential [62]. While traditionally recognized for its nephrotoxic, immunosuppressive, and carcinogenic properties [63], studies have reported its inhibitory activity against several bacterial species [64]. This duality action of both toxicological and antimicrobial makes ochratoxin A, a molecule of particular interest. Although classified by the International Agency for Research on Cancer (IARC) as a possible human carcinogen (Group 2B) [65], recent efforts have focused on characterizing the thresholds at which it may confer antibacterial effects without inducing toxicity. Chronic exposure to ochratoxin A has been linked to renal fibrosis, oxidative stress, DNA damage, and increased cancer risk [65]. Nonetheless, given the escalating global burden of multidrug-resistant *K. pneumoniae* infections, exploring non-traditional antimicrobial candidates, including fungal secondary metabolites, is of high relevance. The inclusion of ochratoxin A in this study is therefore justified by its emerging antibacterial profile and underscores the importance of defining safe therapeutic windows where efficacy can be achieved without compromising host safety.

**Table 1:** Interaction of the ligand molecule permethrin, Ochratoxin A, bromadiolone, and control against 3U4K protein of *K. pneumoniae* mentioning binding energy, non-covalent interaction, interacting amino acids, bond types and their distance

Protein-ligand Complex	Binding energy (kcal/mol)	Amino acid residues	Bond types	Distance (Å)
Permethrin (CID: 40326) + 3U4K	-8.2	TYR155* SER123 TYR117* PRO157* ILE109* PHE124* ARG125* ILE63 ALA127* VAL65* LEU73*	H CH PA A P-S PA A A A A A	4.42877 3.50169 3.9865 2.5674 3.65807 4.49423 4.47488 5.1538 5.02064 5.14364 5.37651
Ochratoxin A (CID: 442530) + 3U4K	-9.1	ARG105* TYR155* SER123 THR119* PHE124* VAL65* PRO157*	H H H H PA A A	2.39643 2.14078 2.49448 2.56831 4.50073 5.17497 4.58066
Bromadiolone (CID: 54680085) + 3U4K	-8.6	TYR117* THR119* ARG125* ILE109* ALA127 ALA108	H H H PS A A	1.78835 2.38131 2.12419 3.68846 5.02661 4.35564
Control (Ciprofloxacin) + 3U4K	-6.8	LYS137* THR142* SER175* PRO42	H H H A	2.4561 3.2456 1.0873 4.5578

H: hydrogen bond; A: alkyl; C-H: carbon hydrogen bond; P-S: Pi-Sigma bond; P-A: Pi-Alkyl. *Active site amino acids

### Molecular dynamic simulation

**Fig. 3:**
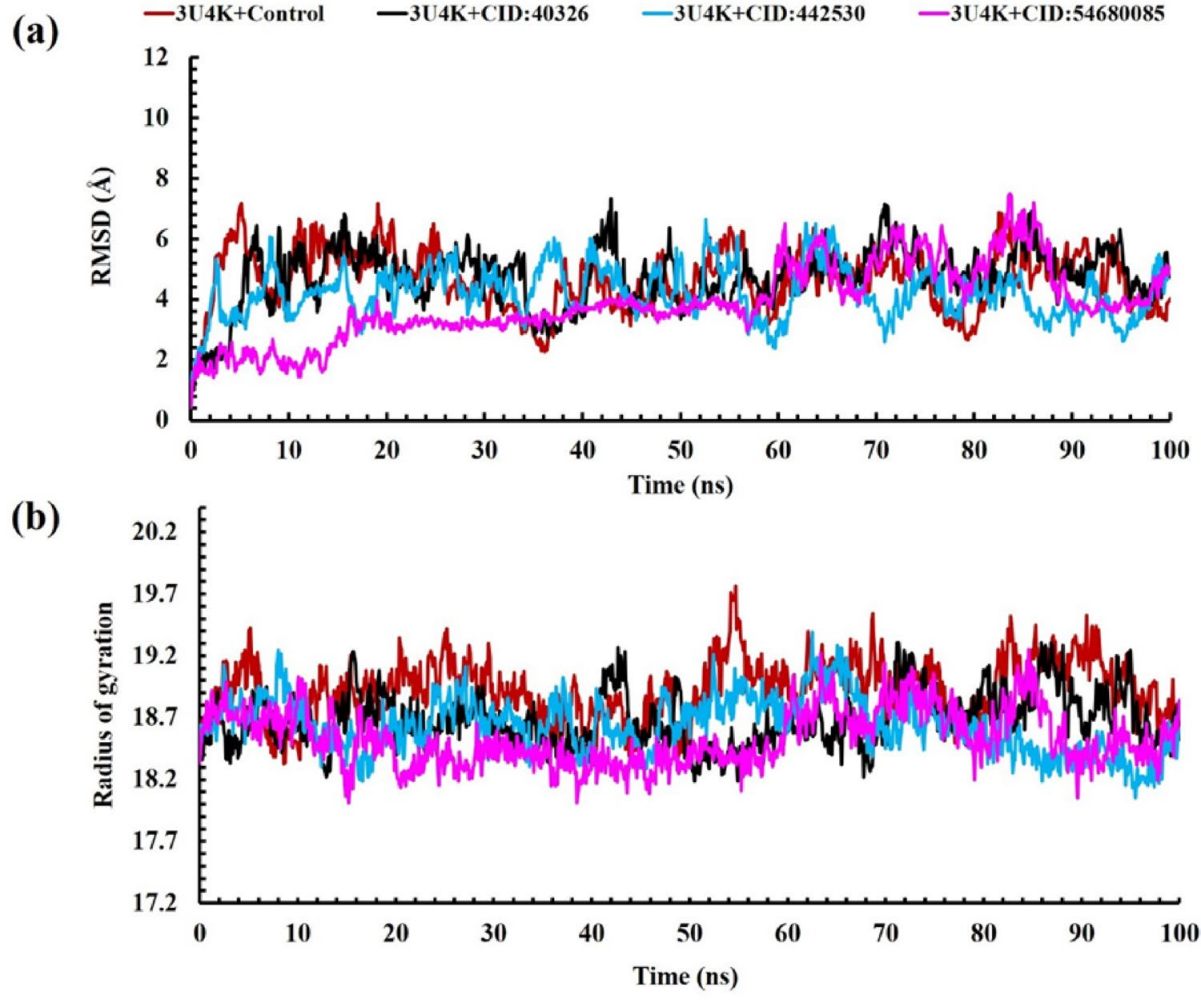
Analysis of **a** RMSD and **b** radius of gyration of complexes between permethrin, bromadiolone, ochratoxin A, and target protein of Fimbria adhesin protein- *Klebsiella pneumonia* (PDB ID: 3U4K) at 100 ns molecular dynamics simulation

To search the progressive characteristics of protein-ligand complexes at the molecular range, elaborate on biological action, understand structure-function relevance, and come up with a message that is very important for drug discovery, Molecular Dynamics (MD) simulations were devoted. It was then directed on the most undertaking complexes to affirm their durability and inflexibility over a 100 ns time direction to identify strong inhibitors. Utilization of analysis metrics including Root Mean Square Deviation (RMSD), Radius of Gyration (Rg), Solvent Accessible Surface Area (SASA), Hydrogen Bond analysis, and Root Mean Square Fluctuation (RMSF) was done to evaluate the complexes, focusing on the Fimbria adhesion protein (PDB ID: 3U4K) complexes as follows in Fig. 3.

### Analysis of RMSD

Root means square deviation (RMSD) is one of the most widely employed metrics for quantifying structural variations in macromolecules, particularly in the context of molecular dynamics (MD) simulations. By computing the average deviation of atomic positions typically the Cα backbone following optimal rigid-body superposition, RMSD provides insight into the conformational stability and equilibration of biomolecular systems over time [66]. In this study, we examined the Cα-RMSD trajectories of the 3U4K protein in complex with three candidate ligands—permethrin, ochratoxin A, and bromadiolone over a 100 ns simulation period to assess the dynamic stability of each complex. As shown in Fig. 3a, each protein–ligand system exhibited distinct fluctuation patterns. The control complex (3U4K–ciprofloxacin) exhibited a mean RMSD of 4.652 Å, closely aligned with the permethrin-bound complex (4.721 Å), which stabilized after approximately 20 ns with moderate fluctuations throughout. Ochratoxin A yielded a slightly lower mean RMSD of 4.216 Å, suggesting improved conformational restraint relative to permethrin. Bromadiolone exhibited the greatest structural stability, with a mean RMSD of 3.863 Å and minimal deviations from the initial structure across the simulation window. The control complex displayed RMSD values ranging from 0.446 Å to 7.169 Å, while permethrin ranged from 0.460 Å to 7.337 Å. Ochratoxin A and bromadiolone maintained more confined fluctuation ranges of 0.468–6.642 Å and 0.429–7.483 Å, respectively. Notably, bromadiolone induced the least conformational perturbation, indicating a stabilizing effect on the protein structure. Previously reported RMSD values for *K. pneumoniae* Carbapenemase-2 (KPC-2) complexes are considerably higher than our findings, ranging from 1.5 to 2.0 nm (15–20 Å), likely due to the high flexibility of active site residues. In contrast, studies investigating flavonoid compounds against *K. pneumoniae* reported RMSD values within a more acceptable range of 0.2–0.5 nm (2–5 Å), indicating stability comparable to the native protein structure and consistency with the data presented in this study. Moreover, only minor conformational changes were observed over the simulation time course [67, 68]. Overall, the lower and more consistent RMSD profiles of ochratoxin A and bromadiolone suggest superior binding stability compared to permethrin and the control, reinforcing their potential as viable inhibitors of the 3U4K adhesin protein.

### Analysis of radius of gyration (Rg)

The radius of gyration (Rg) is used to identify the compactness and the size of the protein molecules [69]. As illustrated in Fig. 3b, Rg values were analysed for 3U4K in complex with ciprofloxacin (control), permethrin, ochratoxin A, and bromadiolone over a 100 ns simulation period. The control complex exhibited an average Rg of 18.929 Å, fluctuating between 18.329 Å and 19.760 Å. Notably, a slight increase in Rg was observed between 52 and 60 ns, with an average of 19.127 Å during that interval, suggesting transient expansion of the protein structure. The 3U4K–permethrin complex showed Rg values ranging from 18.186 Å to 19.309 Å, with a mean of 18.681 Å. Although slightly more compact than the control on average, this complex exhibited larger fluctuations, indicating potential conformational instability or weaker ligand-protein interactions. In comparison, the 3U4K–ochratoxin A complex demonstrated greater compactness and stability, with Rg values spanning 18.052–19.392 Å and a mean of 18.639 Å. While some fluctuations emerged after 40 ns, the structure remained relatively stable overall. The most compact and stable profile was observed in the 3U4K–bromadiolone complex, with Rg values ranging from 18.014 Å to 19.249 Å and a mean of 18.531 Å. Early in the simulation, the complex exhibited the highest compactness across all conditions. Although a moderate increase in Rg was noted between 30 and 60 ns, the structure stabilized near 18.8 Å toward the simulation’s end, indicating a return to a compact, ordered state. Although there are few direct Rg values for OTA protein complexes in the literature, several MD studies provide stability markers indicating that OTA typically interacts with target proteins in a stable manner. OTA recognition studies revealed steady hydrogen bonding structures and minor structural shifts during simulations. These results support OTA’s ability to retain protein integrity during binding [70], as demonstrated by the current study’s Rg data, which showed that OTA sustained its compact 3U4K structure with a mean Rg of 18.639 Å. Overall, the Rg analysis underscores bromadiolone’s ability to confer greater structural compactness and stability to the 3U4K protein compared to the other ligands, reinforcing its potential as a structurally stabilizing agent.

### Analysis of SASA

**Fig. 4:**
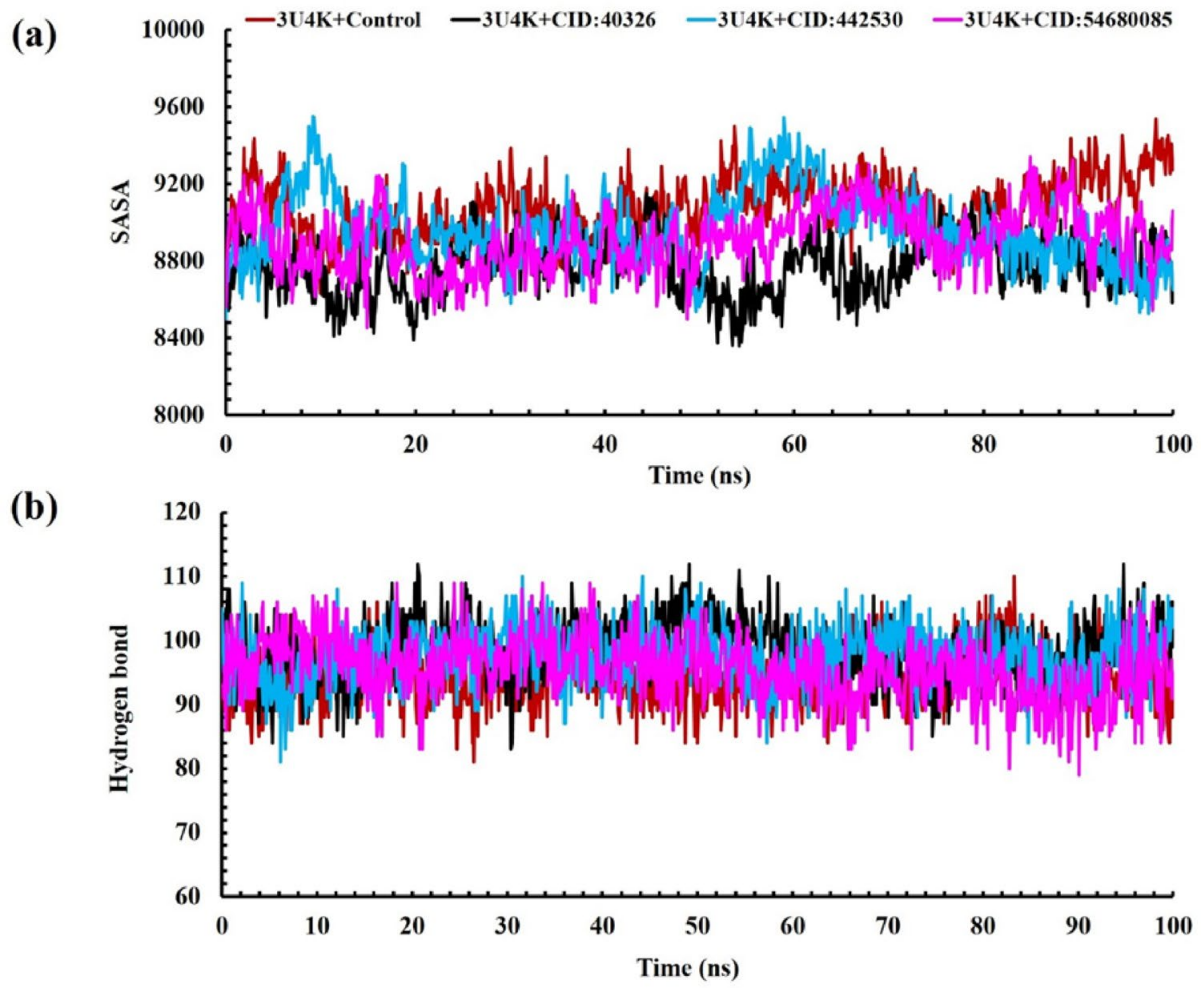
Analysis of **a** SASA and **b** hydrogen bond of complexes between permethrin, bromadiolone, ochratoxin A, and target protein of Fimbria adhesin protein of *Klebsiella pneumonia* (PDB ID: 3U4K) at 100 ns molecular dynamics simulation

One important consideration in the research of protein folding and stability is the solvent-accessible surface area (SASA) of proteins. The SASA profile 3U4K-ligand complexes shown in Fig. 4a assess the protein’s exposure to the diluent, thereby indicating the potential unfolding of the protein throughout 100 ns MD simulation. We can see that ochratoxin A and bromadiolone maintained similar solvent accessibility when bound to protein 3U4K throughout the 100 ns simulation period, indicating stable interactions. For the 3U4K-Control complex, the SASA values vary from 8563.504 Å² to 9538.881 Å² with an average value of 9078.903805 Å², while for the 3U4K-permethrin complex, it ranged between 8358.451 and 9154.07 Å² with a mean value of 8772.420844 Å². This indicates that permethrin promotes slight folding or more compact formation, reducing the surface area of the control complex. The complex of 3U4K-ochratoxin A fluctuates between 8499.79 and 9550.67 Å² with an average value of 8967.439 Å². These relatively lower SASA values indicate a significant stabilization of the complex in a compact conformation, minimizing the solvent-accessible areas, and could imply stronger interactions leading to enhanced folding or tightly packing of the protein structure. The last complex, 3U4K-bromadiolone, compasses within a specific range of about 8453.985 and 9341.602 Å² with a mean value of 8906.158 Å². This suggests that bromadiolone induces moderate compaction in the protein and has moderate stabilizing effects, reducing the solvent surface area to a certain degree. A study on porin proteins of *K. pneumoniae* resistant to carbapenems reported SASA values for various drugs, including cefepime and meropenem (200–450 Å² and 160–400 Å², equilibrium ~ 300 Å²), zinc oxide (160–320 Å², equilibrium ~ 150 Å²), and imipenem (160–320 Å², equilibrium ~ 200 Å²) [71]. The result of OTA contrasts significantly in comparison with the stated data from the prior investigation, reflecting a larger protein system, yet the result for OTA manifests improved stability and compactness. These lower and consistent SASA values suggest that ochratoxin A induces more compact conformations and stable interactions with the 3U4K protein, indicating familiar structural stability. In a nutshell, the overall 100 ns MD simulation of permethrin, ochratoxin A, and bromadiolone SASA values implies that ochratoxin A induces more compact or stable protein conformation as reflected in the reduced solvent exposure and showing the most pronounced effects.

### Analysis of hydrogen bond

Understanding the stability and nature of protein-ligand complex interactions requires careful examination of hydrogen bonds in molecular dynamics (MD) simulations [72]. The specificity and strength of the interaction between the protein and ligand are greatly affected by hydrogen bonding. A better understanding of the complex dynamics, including transitory contacts and binding interface stability, can be obtained by tracking the occurrence of hydrogen bond creation and breaking events throughout the simulation [73]. The amount of hydrogen bonds formed between the given ligands and 3U4K throughout the 100 ns simulation time is displayed in Fig. 4b. All the ligands showed an increase in hydrogen bonding when coupled to 3U4K. On average, 94.38 hydrogen bonds were generated by the 3U4K-ciprofloxacin complex and 98.69 hydrogen bonds by the 3U4K: permethrin complexes. In terms of average hydrogen bond formation, the 3U4K: ochratoxin A complexes achieved 97.62 bonds whereas the 3U4K: bromadiolone complexes reached 95.51 bonds. Everything points to a ligand-protein 3U4K binding interaction that is modest at best. It is worth mentioning that these patterns of hydrogen bonds were consistent throughout the simulation, comparable to what was observed in the apoprotein state. The results showed that ligand-protein interactions are dynamic. Theoretically, they postulated that all ligands possess inhibitory potential, and that different protein targets exhibit different hydrogen bond patterns.

### Analysis of RMSF

**Fig. 5:**
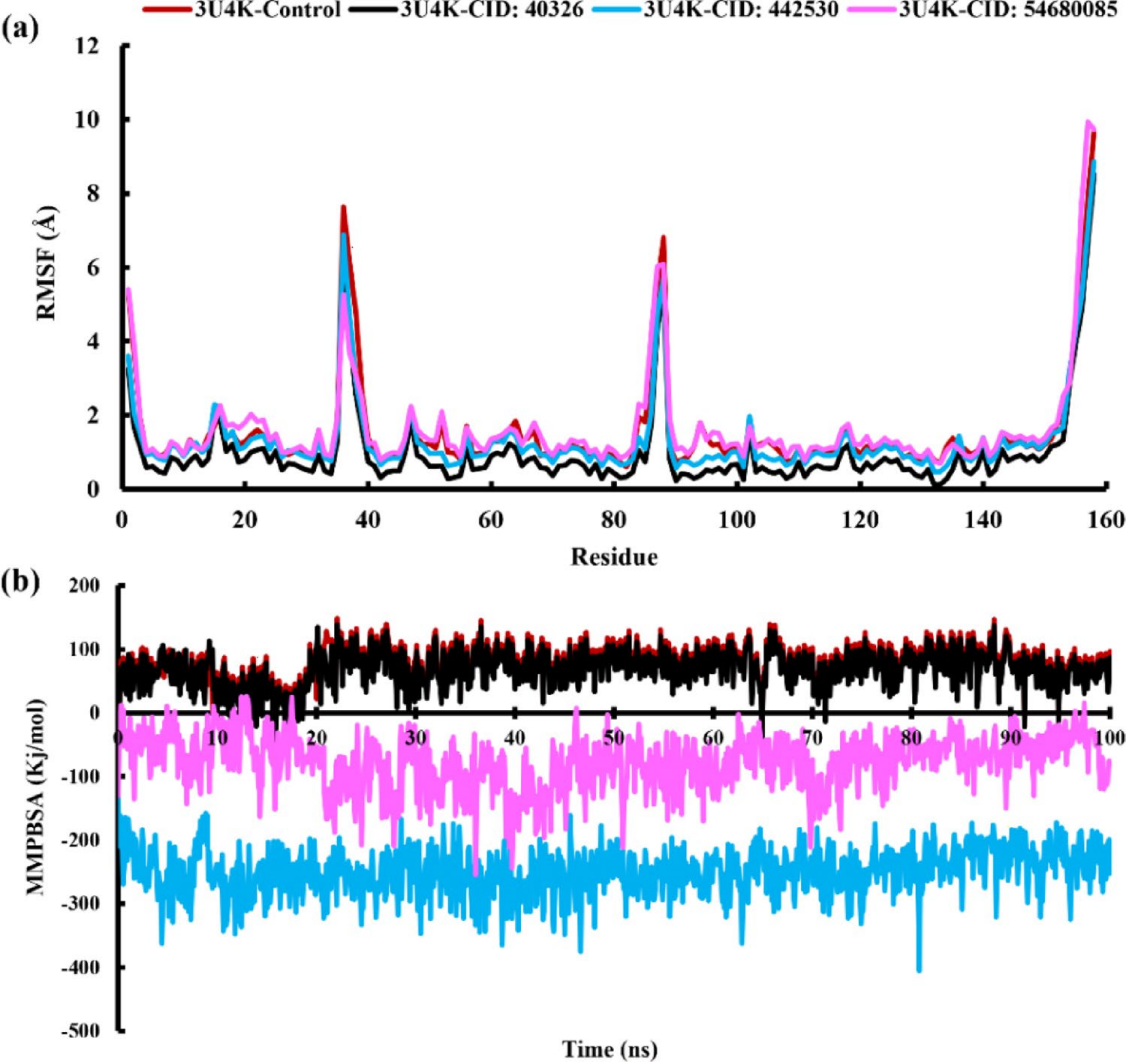
The analysis of **a** RMSF **b** MMGBSA binding free energy of complexes at 100 ns simulation period

RMSF (Root Mean Square Fluctuation) analysis to study the dynamic and flexible behavior of individual atoms or residues in a biomolecular system. By analyzing these variations, we were also able to identify which residues were responsible for them [74]. By using RMSF, one can determine the mobility and flexibility of certain (amino acid) residues. Figure 5a shows the 3U4K-ligand complexes and the degree to which each amino acid residue is amenable, as part of the RMSF profile explanation. Trajectory research shows that when bound to 3U4K, all complexes exhibit nearly identical oscillations. Specifically, the average fluctuations of 3U4K-ciprofloxacin, 3U4K- permethrin, 3U4K- ochratoxin A, and 3U4K-bromadiolone are 1.53 Å, 0.99 Å, 1.34 Å, and 1.65 Å. During the simulation study, all compounds show common fluctuations at the following amino acid residues: LEU158, ILE157, PRO36, SER88, THR156, and ILE87. We observed that 3U4K- ciprofloxacin and 3U4K-ochratoxin A had significant RMSF peaks at ASP37, 3U4K- ciprofloxacin and 3U4K-bromadiolone at GLY1, and 3U4K-ochratoxin A and 3U4K-bromadiolone at CYS155. Functionally significant effects, such as facilitating binding events, allowing structural modifications, or contributing to molecular recognition processes, may result from the enhanced flexibility of these specific residues. All peaks show that control RMSF values are greater than other complexes. Similar RMSF patterns are shown in 3U4K- ochratoxin A and 3U4K- bromadiolone, with lower RMSF values over most regions and significantly lesser variations at the peak, indicating a more stable structure for the protein. The 3U4K-control complex has the highest total protein amenability, but the 3U4K-ochratoxin A and 3U4K-bromadiolone complexes have the lowest. This suggests that the ligands fix the protein better, limiting its flexibility and mobility. The undocked KpLpxC system from a prior investigation showed a lower average RMSD of 2.453 Å over 12 ns but higher residue-specific fluctuations during simulation period, with RMSF peaks reaching 5.415 Å in loop and terminal regions, reflecting intrinsic protein mobility [75]. OTA binding to 3U4K not only maintained stable RMSD values but also reduced overall residue fluctuations, suggesting a ligand-induced rigidification of the protein structure.

### Analysis of MMPBSA

Based on the 100 ns molecular dynamics simulation and MM/PBSA binding energy calculations, the binding affinities of three ligands (permethrin, ochratoxin A, bromadiolone) to the 3U4K protein were evaluated and compared to the control. As shown in the Fig. 5b, the binding energy profiles reveal significant variation in stability and interaction strength over the simulation period. The control complex (3U4K-Control) displayed an average binding energy of 77.15 kcal/mol, remaining consistently in the positive range throughout the 100 ns, which suggests an overall weak or non-favourable binding interaction. Permethrin followed a similar trend, fluctuating within a slightly lower energy window, with an average of 67.70 kcal/mol, indicating unstable and energetically unfavourable binding, as evidenced by the erratic peaks seen in the plot. In contrast, bromadiolone maintained a consistently negative binding energy, averaging − 76.61 kcal/mol. The yellow trace in the graph demonstrates moderate fluctuation but remains within the negative range across the entire timeframe, indicating stable and favourable binding. The most notable result was observed for ochratoxin A, which exhibited the strongest and most stable interaction with 3U4K, with an average binding energy of -249.33 kcal/mol. The grey line shows minimal fluctuation and consistently deep binding energy values, indicating a highly favourable and persistent interaction over the simulation period. The MM/PBSA binding free energies of *K. pneumoniae* MurI (glutamate racemase) top inhibitors reported very weak average binding free energies of − 27.26 ± 3.06 kcal/mol and − 29.53 ± 4.29 kcal/mol [76]. In contrast, OTA demonstrated substantially stronger binding affinity, highlighting its superior interaction potential. Overall, the 100 ns simulation confirms that ochratoxin A is the most potent ligand in terms of binding affinity and stability, followed by bromadiolone, while permethrin and the control lack significant binding potential. This suggests that CID: 442,530 is a promising lead compound for further structural and functional optimization.

### Principal components analysis (PCA)

**Fig. 6:**
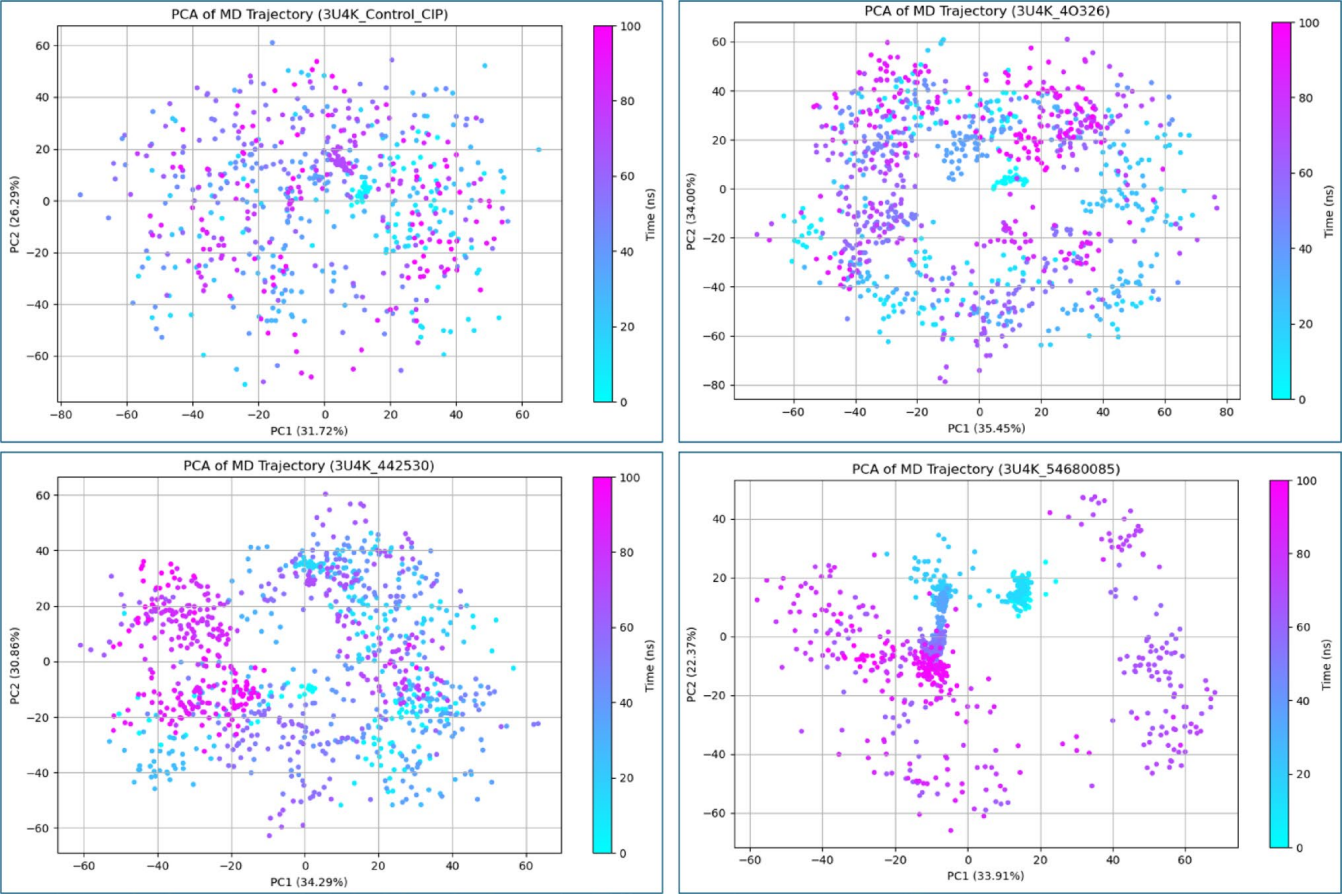
Principal Component Analysis (PCA) of protein-ligand complexes involving PDB: 3U4K

In this study, Principal Component Analysis (PCA) was employed to evaluate the conformational dynamics of the PDB: 3U4K protein in complex with different ligands, using the ciprofloxacin-bound complex as the control. The control complex exhibited moderate structural variability, with PC1 and PC2 accounting for 31.72% and 26.29% of the total motion, respectively. Among the test ligands, CID: 40326 induced the most significant conformational changes (PC1: 35.45%, PC2: 34.00%), indicating enhanced flexibility and large-scale collective motions likely driven by dynamic interactions within the binding pocket. Similarly, CID: 442530 showed high variability (PC1: 34.29%, PC2: 30.86%), suggesting notable rearrangements in the protein structure upon binding. In contrast, CID: 54680085 displayed a more moderate dynamic shift (PC1: 33.91%, PC2: 22.37%), reflecting a relatively stable profile with less perturbation of the overall protein conformation. These findings highlight how different ligands uniquely modulate the structural behavior of the protein, potentially influencing their binding affinity and therapeutic efficacy (Fig. 6).

PCA was performed on 100 ns molecular dynamics trajectories to investigate the conformational dynamics of PDB: 3U4K in complex with ciprofloxacin (control) and three test ligands: CID: 40326, CID: 442530, and CID: 54680085. The plot illustrates the distribution of motion captured by the first two principal components (PC1 and PC2), which represent the most significant collective structural fluctuations. Ciprofloxacin (PC1: 31.72%, PC2: 26.29%) served as the reference for baseline dynamics. CID: 40326 (PC1: 35.45%, PC2: 34.00%) and CID: 442530 (PC1: 34.29%, PC2: 30.86%) induced higher conformational variability, whereas CID: 54680085 (PC1: 33.91%, PC2: 22.37%) showed relatively lower structural shifts.

### ADMET analysis

The computational prediction of a compound’s Absorption, Distribution, Metabolism, Excretion, and Toxicity (ADMET) properties is vital in drug development to evaluate the pharmacokinetic behavior and potential toxicity prediction [77, 78]. Prediction of ADMET through computer-based analysis is useful for the prediction of variable parameters such as drug-likeness, toxicity occurrence with pharmacokinetic estimation, and assays concerning experiments. In this investigation, computational models were assessed to anticipate the viable characteristics of ADMET for permethrin, ochratoxin A, and bromadiolone via SwissADME and PkCsm web-based server, for determination of the viability of the candidates guiding drug development (Table 2). Water solubility, CaCO2 permeability, and percentage of human intestinal absorption (HIA) were evaluated as Absorption properties. Ochratoxin A showed the highest water solubility of -2.989, following bromadiolone (-5.57) and permethrin (-6.853). These values suggest adequate solubility compared to ideal ranges where compounds with LogSw values less than (more negative than) -6 are considered to be poorly soluble [79, 80]. CaCO2 permeability is used to predict oral absorption. CaCO2 permeability is much higher in permethrin (1.028) in comparison with ochratoxin A (0.935) and bromadiolone (0.203). Moreover, the predicted percentage of HIA was found to be 91.958% for permethrin, 52.448% for ochratoxin A, and 91.319 for bromadiolone. These numbers imply variations in the three compounds’ potential for absorption, with permethrin exhibiting higher intestinal absorption compared to ochratoxin A and bromadiolone.

**Table 2:** ADMET prediction of permethrin, Ochratoxin A and Bromadiolone from SwissADME and PKCSM tools where each molecule exhibited nearly ideal drug-like qualities

Parameters		Molecules		
		Permethrin	Ochratoxin A	Bromadiolone
Absorption	Water solubility	-6.853	-2.989	-5.57
	CaCO2 permeability	1.028	0.935	0.203
	Intestinal absorption (human) (%)	91.958	52.448	91.319
Distribution	VDss (human) (log L/kg)	0.541	-1.94	-1.199
	BBB permeability	-0.021	-0.887	-0.368
	CNS permeability	-1.399	-3.013	-1.29
Metabolism	CYP1A2 inhibitor	Yes	No	No
	CYP2D6 inhibitor	No	No	No
	P-glycoprotein I inhibitor	Yes	No	Yes
	P-glycoprotein II inhibitor	Yes	No	Yes
Excretion	Total Clearance	-0.167	0.018	-0.06
	Renal OCT2 substance	No	No	No
Toxicity	AMES toxicity	Yes	No	No
	Max. tolerated dose (human) (log mg/kg/day)	0.466	0.645	0.463
	hERG I inhibitor	No	No	No
	hERG II inhibitor	No	No	Yes
	Oral Rat Acute Toxicity (LD50) (mol/kg)	3.164	2.808	3.295
	Oral Rat Chronic Toxicity (LOAEL) (log mg/kgbw/day)	1.11	2.442	0.279
	Hepatotoxicity	No	Yes	No
	Skin sensitization	No	No	No

The distribution of compounds within the body was assessed through parameters such as volume of distribution (VDss), blood-brain barrier (BBB) permeability, and central nervous system (CNS) permeability [81]. The blood-brain barrier (BBB) is a sophisticated barrier that segregates the central nervous system (CNS) from peripheral tissues [82]. Its primary function is to uphold CNS homeostasis by regulating the movement of substances, essential nutrients, and cells between the bloodstream and the brain [83]. Additionally, the BBB plays a crucial role in eliminating cellular waste products and toxins from the brain into the bloodstream [84]. permethrin, ochratoxin A, and bromadiolone exhibited varied BBB permeability values, with permethrin showing a value of − 0.021, ochratoxin A showing − 0.887, and bromadiolone showing a value of -0.368. These values suggest that permethrin, ochratoxin A, and bromadiolone are moderately permeable according to Standard thresholds for BBB penetration. permethrin displayed a high volume of distribution (VDss) in humans (0.541 log L/kg) compared to ochratoxin A (-1.94 log L/kg) and bromadiolone (-1.199 log L/kg), indicating comprehensive allocation to the body. In addition, ochratoxin A showed significantly lower central nervous system (CNS) permeability (-3.013) compared to permethrin (-1.399) and bromadiolone (-1.29), suggesting reduced potential for CNS penetration. In the case of central nervous system permeability, ochratoxin A showed enormously lower results (-3.013) compared to permethrin (-1.399) and bromadiolone (-1.29), suggesting reduced potential for CNS penetration. The metabolism of permethrin, ochratoxin A, and bromadiolone were assessed on focusing their ability to inhibit key cytochrome P450 (CYP) enzymes, like CYP1A2, CYP2D6, P- glycoprotein I, and P-glycoprotein II. ochratoxin A and bromadiolone showed no evidence of inhibition for CYP1A2 or CYP2D6 enzymes, indicating a low chance for metabolic interactions or toxicity issues. This suggests that ochratoxin A and bromadiolone have favorable metabolic profiles, which lower the likelihood of reducing the risk of drug-drug interactions. Permethrin shows no inhibition of CYP2D6 enzymes but inhibition of CYP1A2 enzymes. This suggests that permethrin has the possibility of drug-drug interactions when it is administered with drugs metabolized by CYP1A2, so this should be considered carefully.

Furthermore, P-glycoproteins I or II are not inhibited by ochratoxin A, suggesting that the drug efflux mechanism is unruffled. But permethrin and bromadiolone inhibit P-glycoprotein I and II, and this suggests that permethrin and bromadiolone may influence the absorption or distribution of concurrently administered medications that are P-glycoprotein transporter substrates. Permethrin (-0.167) and bromadiolone (-0.06) exhibit low clearance, while permethrin exhibits high clearance. These values point to effective excretion from the body, which lowers the potential risk of accumulation and related toxicity. The OCT2 transport protein is not predicted to interact with any of the three compounds. According to various criteria, the toxicity profits of permethrin, ochratoxin A, and bromadiolone are similar and comparable. The absence of AMES toxicity in ochratoxin A and bromadiolone suggests that they have no mutagenic prospect. However, permethrin exhibits AMES toxicity that determines its potential carcinogenicity. Permethrin (0.466 log mg/kg/day) and bromadiolone (0.463 log mg/kg/day) have lower maximum tolerated dose (MTD) than ochratoxin A (0.645 log mg/kg/day), suggesting variation in human tolerance for them. Additionally, hERG I or hERG II are not inhibited by either of the compounds, except bromadiolone, which inhibits hERG II. This implies a lower risk of cardiovascular toxicity. In terms of acute toxicity in rats, permethrin, ochratoxin A, and bromadiolone all exhibit almost identical LD50 values (3.164, 2.808, and 3.295 mol/kg, respectively), indicating similar acute toxicity profiles. In compared to permethrin (1.11 log mg/kg_bw/day) and bromadiolone (0.279 log mg/kg_bw/day), ochratoxin A had higher chronic toxicity (LOAEL) in rats (2.442 log mg/kg/bw/day), urging variations in long term toxic effects. Notably, ochratoxin A is predicted to be hepatotoxic, whereas permethrin and bromadiolone are not associated with hepatotoxicity. Additionally, none of the compounds are predicted to cause skin sensitization. Evaluations of the Lipinski rule and pharmacokinetics were performed for permethrin, ochratoxin A, and bromadiolone in accordance with standard guidelines and in view of previous findings (Table 3) [85–87].

**Table 3:** Using Lipinski rules the prediction of pharmacokinetics properties for permethrin, ochratoxin A and bromadiolone from Pkcsm and SwissADME server we found each compound exhibited nearly ideal drug-like qualities.

Pubchem ID	CID: 40326	CID: 442530	CID: 54680085
Molecular Name	Permethrin	Ochratoxin A	Bromadiolone
Formula	C21H20Cl2O3	C20H18ClNO6	C30H23BrO4
Molecular Weight	391.3 g/mol	403.8 g/mol	527.41 g/mol
Number rotatable bonds	7	6	6
Number H-bond acceptors	3	6	4
Number H-bond donors	0	3	2
TPSA	35.53 Å²	112.93 Å²	70.67 Å²
Log Po/w (WLOGP)	5.96	2.57	6.86
Lipinski violations	1	0	2
Bioavailability Score	0.55	0.56	0.17

Permethrin, characterized by a molecular weight (MW) of 391.3 Da, exhibited 7 rotatable bonds, and 3 hydrogen bond acceptors, leading to a (TPSA) of 35.53 Å2. These parameters are consistent with Lipinski’s rule of five, except for the violation of MLOGP > 4.15. Ochratoxin A has a molecular weight of 403.8 Da and exhibits 6 rotatable bonds, 6 hydrogen bond acceptors, and 3 hydrogen bond donors, resulting in a TPSA of 112.93 Å2, following Lipinski’s rule of five. Bromadiolone has a molecular weight of 527 Da and exhibits 6 rotatable bonds, 4 hydrogen bond acceptors, and 2 hydrogen bond donors, resulting in a TPSA of 70.67 Å2, following Lipinski’s rule of five. Bromadiolone has 2 violations of Lipinski’s rule of five, MW > 500 and MLOGP > 4.15. The lipophilicity values, represented by LogPo/w, were 5.96 for permethrin, 2.57 for ochratoxin A, and 6.86 for bromadiolone. Only ochratoxin A has the criteria for good human oral bioavailability (0 < logP < 3) [88]. Likewise, bromadiolone displayed an inferior bioavailability score (0.17) compared to permethrin (0.55) and ochratoxin A (0.56), indicating probable objection in oral absorption for bromadiolone.

The advantageous pharmacokinetic profiles and potent binding affinities of these fungal metabolites suggest that natural compounds may yet offer effective alternatives to counter MDR *K. pneumoniae*. This study emphasizes the necessity for in depth investigation on these findings through in vitro and in vivo assays to bolster aforementioned compounds’ efficacy and safety even more. Notably, the metabolic resilience and toxicity profiles observed in ochratoxin A provides a promising candidacy for future drug development aimed at nosocomial infection control [89]. Expanding investigative efforts into naturally derived antimicrobials could also yield broader-spectrum strategies against other MDR pathogens in clinical environments [90]. The molecular insights obtained here enrich our understanding of fungal metabolites’ inhibitory potential against *K. pneumoniae* and open pathways for novel antimicrobial development, thereby addressing a critical gap in the global effort to counter antibiotic resistance.

### In vitro antibacterial activity

**Fig. 7:**
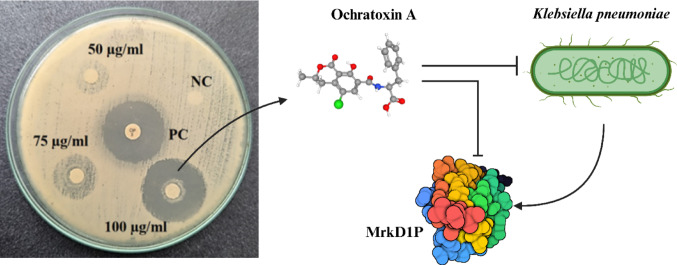
Antibacterial activity of compound ochratoxin A against *K. pneumoniae* in three multiple concentrations where ciprofloxacin is used as a positive control, and a possible OTA action on MrkD1P may inhibit the *K. pneumoniae* growth

The antimicrobial activity of ochratoxin A (OTA) against *K. pneumoniae* was assessed using disc diffusion and broth dilution methods. In the disc diffusion assay, OTA demonstrated a concentration-dependent antibacterial effect, with the diameter of the inhibition zone increasing from 18.5 ± 0.75 mm at 50 µg/disc to 27 ± 0.33 mm at 75 µg/disc, and reaching 34 ± 0.67 mm at 100 µg/disc. Notably, at the highest tested concentration, the inhibitory effect of OTA slightly exceeded that of the standard antibiotic ciprofloxacin (33 mm), indicating strong antibacterial potency (Fig. 7; Table 4). No inhibition was observed with the negative control (nutrient disc without compound), confirming that the activity was attributable to OTA alone. To quantitatively validate these findings, minimum inhibitory concentration (MIC) and minimum bactericidal concentration (MBC) values were determined using the broth dilution method. OTA exhibited a MIC of 18.33 ± 0.72 µg/mL and an MBC of 39.33 ± 1.36 µg/mL (*p* < 0.05), indicating potent bacteriostatic and bactericidal activity at relatively low concentrations (Table 4). The MIC and MBC values were consistent with the disc diffusion results, further substantiating the antimicrobial efficacy of OTA against *K. pneumoniae*. Mechanistically, the antibacterial activity of OTA may be mediated through the inhibition of the MrkD1P adhesin protein, a critical component of the type 3 fimbrial operon responsible for mediating bacterial adhesion and biofilm formation. Both in silico molecular docking and in vitro validation assays confirmed strong binding affinity and functional inhibition of MrkD1P by OTA (Fig. 7). In addition, OTA has been previously reported to inhibit phenylalanine t-RNA synthetase, an essential enzyme in bacterial protein translation, offering a possible secondary mechanism of action [91]. These multimodal inhibitory effects likely contribute synergistically to the overall antibacterial outcome. These findings reveal that OTA possesses substantial antimicrobial activity against *K. pneumoniae*, acting through disruption of critical adhesion and translation pathways. Given its efficacy at low concentrations and its distinct mechanisms of action, OTA warrants further investigation as a candidate for development into alternative or adjunctive therapies, particularly considering increasing antimicrobial resistance. In Gram-negative pathogens such as *K. pneumoniae*, OTA exerts antibacterial effects mainly disrupting protein synthesis by inhibits phenylalanyl-tRNA synthetase, preventing tRNA^Phe charging and stalling peptide elongation, which induces a stringent response and global inhibition of bacterial growth [91, 92]. This translational blockade not only halts protein production but also impairs virulence factor synthesis. Secondary effects include induction of oxidative stress, DNA damage, and inhibition of cell wall biosynthesis, although uptake barriers such as the outer membrane render Gram-negative bacteria relatively less sensitive than Gram-positive species [91, 93]. These findings support the primary mechanism of OTA as a competitive inhibitor of protein synthesis, with additional stress responses amplifying its antibacterial activity. Beyond this canonical mechanism, our docking analyses indicate that OTA can form stable interactions with the MrkD1P adhesin of *K. pneumoniae*. Such binding could potentially block type V collagen engagement, reducing adhesion, biofilm initiation, and pathogenicity (Fig. 7). While this proposed anti-adhesion mechanism requires experimental validation, it highlights a complementary pathway through which OTA may exert anti-virulence effects.

**Table 4:** Zone of Inhibition (mm) of Ochratoxin A against *Klebsiella pneumoniae.* Data are presented as mean ± SE with three biological replications.

Compound	50 µg/disc	75 µg/disc	100 µg/disc	Positive control (Pc)	Negative control (NC)	MIC (µg/mL)	MBC (µg/mL)
Ochratoxin A	18.5 ± 0.75^a^	27 ± 0.33^b^	34 ± 0.67^c^	33	N/A	18.33 ± 0.72	39.33 ± 1.36

Different superscript letters (a–c) indicate statistically significant differences at *p* < 0.05

## Conclusion

This study identifies the type 3 fimbrial adhesin MrkD1P as an underexplored anti-virulence target in *K. pneumoniae* and provides, to our knowledge, the first integrated computational experimental evaluation of a fungal metabolite against this adhesin. Among 329 screened compounds, ochratoxin A (OTA) emerged as the top candidate, consistently showing strong binding affinity, stable interactions in MD simulation, MM-PBSA analyses, and measurable in vitro antibacterial activity. These findings highlight MrkD1P as a tractable intervention point and establish a framework for developing metabolite-inspired, anti-adhesion therapeutics against multidrug-resistant *K. pneumoniae*. Further study should include target-specific functional assays to evaluate OTA’s impact on MrkD1P-mediated adhesion and biofilm formation, alongside in vivo efficacy and pharmacokinetics studies, as well as medicinal chemistry efforts to enhance potency and mitigate potential liabilities.

## Supplementary Information

Below is the link to the electronic supplementary material.

Supplementary Material 1

## Data Availability

No datasets were generated or analysed during the current study.
