# Supplementary material for: Fungal metabolite Ochratoxin A inhibits MrkD1P of multidrug-resistant Klebsiella pneumoniae: Integrated computational and in vitro validation
Source: J Comput Aided Mol Des. 2025 Sep 16;39(1):81. doi: 10.1007/s10822-025-00661-w (PMC12441064; doi:10.1007/s10822-025-00661-w)
Supplement: Supplementary file 1 — Supplementary Material 1 [file 10822_2025_661_MOESM1_ESM.docx]

*Article type: Research article*

***Supplementary file***

**Fungal metabolite ochratoxin A inhibits MrkD1P of multidrug-resistant *Klebsiella pneumoniae*: Integrated computational and in vitro validation**

**Md Roqunuzzaman^1,#^, Ariful Islam^1,#^, Sumaiya Jahan Supti^1,#^, Mahbub Hasan Rifat^1^, Mohammad Saiful Islam^1^, Ummay Habiba Ananna^1^, Khalid Saifullah Tusher^1^, Aamal A. Al-Mutairi^2^, Magdi E. A. Zaki^2^, Subir Sarker^3,*^, Md. Eram Hosen^3, 4,*^**

**Supplementary Table S1.** Interaction of the 329-ligand molecule and control against 3U4K protein of *K. pneumoniae* mentioning binding energy, non-covalent interaction, interacting amino acids, bond types and their distance. Note: *H: Hydrogen bon; A: Alkyl; C-H: Carbon Hydrogen bond; P-S: Pi-Sigma bond; P-A: Pi-Alkyl. *Active site amino acids.*

| **Protein-ligand Complex** | **Binding energy (kcal/mol)** | **Amino acid residues** | **Bond types** | **Distance (Å)** |
| --- | --- | --- | --- | --- |
| **Control (Ciprofloxacin) +3U4K** | **-6.8** | LYS137* | H | 2.4561 |
|  |  | THR142* | H | 3.2456 |
|  |  | SER175* | H | 1.0873 |
|  |  | PRO42 | A | 4.5578 |
| **Ochratoxin A (CID: 442530) + 3U4K** | **-9.1** | ARG105* | H | 2.39643 |
|  |  | TYR155* | H | 2.14078 |
|  |  | SER123 | H | 2.49448 |
|  |  | THR119* | H | 2.56831 |
|  |  | PHE124* | PA | 4.50073 |
|  |  | VAL65* | A | 5.17497 |
|  |  | PRO157* | A | 4.58066 |
| **Bromadiolone (CID: 54680085) + 3U4K** | **-8.6** | TYR117* | H | 1.78835 |
|  |  | THR119* | H | 2.38131 |
|  |  | ARG125* | H | 2.12419 |
|  |  | ILE109* | PS | 3.68846 |
|  |  | ALA127 | A | 5.02661 |
|  |  | ALA108 | A | 4.35564 |
| Permethrin (CID: 40326) + 3U4K | **-8.2** | TYR155* | H | 4.42877 |
|  |  | SER123 | CH | 3.50169 |
|  |  | TYR117* | PA | 3.9865 |
|  |  | PRO157* | A | 2.5674 |
|  |  | ILE109* | P-S | 3.65807 |
|  |  | PHE124* | PA | 4.49423 |
|  |  | ARG125* | A | 4.47488 |
|  |  | ILE63 | A | 5.1538 |
|  |  | ALA127* | A | 5.02064 |
|  |  | VAL65* | A | 5.14364 |
|  |  | LEU73* | A | 5.37651 |
| **44584013+**  **3U4K** | **-8** | A:ARG125:HH22 | Conventional Hydrogen Bond | 2.13541 |
|  |  | N:UNK1:C | Alkyl | 4.25942 |
|  |  | N:UNK1:C | Alkyl | 5.05972 |
|  |  | N:UNK1:C | Alkyl | 3.89126 |
|  |  | N:UNK1:C | Alkyl | 5.17827 |
|  |  | A:PHE124 | Pi-Alkyl | 5.29193 |
|  |  | A:PHE124 | Pi-Alkyl | 4.30197 |
| **139583786+**  **3U4K** | **-8** | A:SER75:HG | Conventional Hydrogen Bond | 1.75814 |
|  |  | A:TYR117:HH | Conventional Hydrogen Bond | 1.95842 |
|  |  | N:UNK1:H | Conventional Hydrogen Bond | 2.35637 |
|  |  | A:ALA108 | Alkyl | 5.19824 |
|  |  | A:ALA108 | Alkyl | 4.363 |
|  |  | A:ALA127 | Alkyl | 5.42018 |
| **44584013+**  **3U4K** | **-7.9** | A:ARG125:HH22 | Conventional Hydrogen Bond | 2.13541 |
|  |  | N:UNK1:C | Alkyl | 4.25942 |
|  |  | N:UNK1:C | Alkyl | 5.05972 |
|  |  | N:UNK1:C | Alkyl | 3.89126 |
|  |  | N:UNK1:C | Alkyl | 5.17827 |
|  |  | A:PHE124 | Pi-Alkyl | 5.29193 |
|  |  | A:PHE124 | Pi-Alkyl | 4.30197 |
| **6437365+**  **3U4K** | **-7.8** | A:GLN86:HN | Conventional Hydrogen Bond | 2.33184 |
|  |  | N:UNK1:C | Alkyl | 4.5045 |
|  |  | N:UNK1:C | Alkyl | 4.41487 |
|  |  | N:UNK1:C | Alkyl | 4.38124 |
|  |  | N:UNK1:C | Alkyl | 4.91886 |
| **4476324+**  **3U4K** | **-7.8** | N:UNK1:C | Carbon Hydrogen Bond | 3.40001 |
|  |  | A:ARG125:HN | Pi-Donor Hydrogen Bond | 3.02153 |
|  |  | A:PHE124 | Pi-Pi Stacked | 4.43203 |
|  |  | N:UNK1 | Pi-Alkyl | 5.03307 |
|  |  | N:UNK1 | Pi-Alkyl | 5.15683 |
|  |  | N:UNK1 | Pi-Alkyl | 4.6117 |
| **444679+**  **3U4K** | **-7.8** | A:ARG125 | Alkyl | 5.07224 |
|  |  | A:ALA127 | Alkyl | 3.73998 |
|  |  | N:UNK1:C | Alkyl | 4.60661 |
|  |  | N:UNK1:C | Alkyl | 4.96755 |
|  |  | A:PHE124 | Pi-Alkyl | 5.28991 |
|  |  | A:TYR155 | Pi-Alkyl | 5.27346 |
|  |  | A:TYR155 | Pi-Alkyl | 5.44372 |
| **6440671+**  **3U4K** | **-7.7** | A:ARG125:HN | Conventional Hydrogen Bond | 1.94474 |
|  |  | A:TYR117 | Pi-Pi Stacked | 5.63885 |
|  |  | A:ALA127 | Alkyl | 3.74736 |
|  |  | N:UNK1:C | Alkyl | 5.37643 |
| **443440+**  **3U4K** | **-7.7** | A:TYR117:HH | Conventional Hydrogen Bond | 2.77827 |
|  |  | N:UNK1:H | Conventional Hydrogen Bond | 1.98897 |
|  |  | A:ALA108 | Alkyl | 4.53115 |
|  |  | A:ALA127 | Alkyl | 4.41747 |
| **139588051+**  **3U4K** | **-7.7** | N:UNK1:C | Carbon Hydrogen Bond | 3.3977 |
|  |  | A:PHE124 | Pi-Pi Stacked | 4.54538 |
|  |  | N:UNK1 | Pi-Alkyl | 5.38299 |
|  |  | N:UNK1 | Pi-Alkyl | 5.12638 |
| **139584550+**  **3U4K** | **-7.7** | A:ASN70:HD21 | Conventional Hydrogen Bond | 2.14714 |
|  |  | A:SER123:HN | Conventional Hydrogen Bond | 2.49767 |
|  |  | A:ARG125:HH22 | Conventional Hydrogen Bond | 2.64547 |
|  |  | A:TYR117 | Pi-Alkyl | 5.49895 |
| **13892285+**  **3U4K** | **-7.7** | A:TYR117:HH | Conventional Hydrogen Bond | 1.98326 |
|  |  | N:UNK1:H | Conventional Hydrogen Bond | 1.9922 |
|  |  | A:ALA108 | Alkyl | 3.48633 |
|  |  | A:ALA127 | Alkyl | 3.84876 |
|  |  | A:PHE124 | Pi-Alkyl | 4.59184 |
| **403923+**  **3U4K** | **-7.6** | A:SER75:HG | Conventional Hydrogen Bond | 2.24041 |
|  |  | A:TYR117:HH | Conventional Hydrogen Bond | 2.46565 |
|  |  | A:THR119:CG2 | Pi-Sigma | 3.9747 |
|  |  | N:UNK1:C | Alkyl | 4.10364 |
|  |  | N:UNK1:C | Alkyl | 4.01257 |
|  |  | N:UNK1:C | Alkyl | 3.72938 |
|  |  | N:UNK1:C | Alkyl | 5.11655 |
|  |  | A:PHE124 | Pi-Alkyl | 4.60975 |
| **367425+**  **3U4k** | **-7.6** | A:ARG125:HN | Conventional Hydrogen Bond | 1.81074 |
|  |  | N:UNK1:H | Conventional Hydrogen Bond | 2.25108 |
|  |  | N:UNK1 | Pi-Alkyl | 5.47461 |
| **159013642+**  **3U4K** | **-7.6** | A:GLY87:CA | Pi-Sigma | 3.71083 |
|  |  | A:VAL91:CG2 | Pi-Sigma | 3.82362 |
|  |  | A:VAL91:CG2 | Pi-Sigma | 3.83885 |
|  |  | A:THR138:CG2 | Pi-Sigma | 3.88665 |
|  |  | N:UNK1 | Pi-Pi T-shaped | 4.95824 |
|  |  | A:ALA88 | Alkyl | 5.10906 |
|  |  | N:UNK1 | Pi-Alkyl | 4.75955 |
|  |  | N:UNK1 | Pi-Alkyl | 5.43112 |
|  |  | N:UNK1 | Pi-Alkyl | 5.49115 |
| **139585698+**  **3U4K** | **-7.6** | A:ARG105:HH11 | Conventional Hydrogen Bond | 2.89816 |
|  |  | A:THR119:HG1 | Conventional Hydrogen Bond | 2.80254 |
|  |  | A:THR119:HG1 | Conventional Hydrogen Bond | 2.02864 |
|  |  | A:ARG125:HH22 | Conventional Hydrogen Bond | 3.01486 |
|  |  | A:ALA127:HN | Conventional Hydrogen Bond | 2.68137 |
|  |  | A:TYR155:HH | Conventional Hydrogen Bond | 2.59758 |
|  |  | A:ALA108 | Alkyl | 4.55073 |
|  |  | A:ILE109 | Alkyl | 5.16302 |
|  |  | N:UNK1:C | Alkyl | 4.77381 |
|  |  | N:UNK1:C | Alkyl | 4.46704 |
|  |  | N:UNK1:C | Alkyl | 5.446 |
|  |  | A:PHE124 | Pi-Alkyl | 4.62298 |
| **10907072+**  **3U4k** | **-7.6** | A:ARG125:HN | Conventional Hydrogen Bond | 2.28165 |
|  |  | A:ARG125:HH22 | Conventional Hydrogen Bond | 2.60374 |
|  |  | N:UNK1:H | Conventional Hydrogen Bond | 2.03778 |
|  |  | A:PHE124 | Pi-Alkyl | 5.38014 |
| **10181133+**  **3U4K** | **-7.6** | A:ALA127 | Alkyl | 3.78463 |
|  |  | N:UNK1:C | Alkyl | 4.57789 |
|  |  | N:UNK1:C | Alkyl | 5.35427 |
|  |  | N:UNK1:C | Alkyl | 4.53568 |
|  |  | A:PHE124 | Pi-Alkyl | 5.3526 |
|  |  | A:PHE124 | Pi-Alkyl | 4.58951 |
| **4476323+**  **3U4K** | **-7.4** | A:ALA127:HN | Conventional Hydrogen Bond | 2.42562 |
|  |  | A:TYR155:HH | Conventional Hydrogen Bond | 2.08539 |
|  |  | A:PHE124 | Pi-Pi Stacked | 4.27597 |
|  |  | N:UNK1 | Pi-Alkyl | 5.06722 |
|  |  | N:UNK1 | Pi-Alkyl | 5.35194 |
|  |  | N:UNK1 | Pi-Alkyl | 4.5719 |
| **444679+**  **3U4K** | **-7.4** | A:ARG125 | Alkyl | 5.07224 |
|  |  | A:ALA127 | Alkyl | 3.73998 |
|  |  | N:UNK1:C | Alkyl | 4.60661 |
|  |  | N:UNK1:C | Alkyl | 4.96755 |
|  |  | A:PHE124 | Pi-Alkyl | 5.28991 |
|  |  | A:TYR155 | Pi-Alkyl | 5.27346 |
|  |  | A:TYR155 | Pi-Alkyl | 5.44372 |
| **367425+**  **3U4K** | **-7.4** | A:ARG125:HN | Conventional Hydrogen Bond | 1.81074 |
|  |  | N:UNK1:H | Conventional Hydrogen Bond | 2.25108 |
|  |  | N:UNK1 | Pi-Alkyl | 5.47461 |
| **1794427+**  **3U4K** | **-7.4** | A:ALA28:HN | Conventional Hydrogen Bond | 2.092 |
|  |  | N:UNK1:H | Conventional Hydrogen Bond | 1.97339 |
|  |  | N:UNK1:H | Conventional Hydrogen Bond | 2.39561 |
|  |  | N:UNK1 | Pi-Alkyl | 4.49577 |
| **9885027+**  **3U4K** | **-7.3** | A:ARG125:HN | Conventional Hydrogen Bond | 2.81145 |
|  |  | A:ARG125:HH21 | Conventional Hydrogen Bond | 2.96156 |
|  |  | N:UNK1:H | Conventional Hydrogen Bond | 2.18845 |
|  |  | N:UNK1:H | Conventional Hydrogen Bond | 2.19872 |
|  |  | A:PHE124:CA | Carbon Hydrogen Bond | 3.56355 |
|  |  | A:ILE109:CD | Pi-Sigma | 3.83158 |
|  |  | N:UNK1 | Pi-Alkyl | 4.99298 |
|  |  | N:UNK1 | Pi-Alkyl | 4.51321 |
|  |  | N:UNK1 | Pi-Alkyl | 4.33938 |
|  |  | N:UNK1 | Pi-Alkyl | 4.5285 |
|  |  | N:UNK1 | Pi-Alkyl | 5.06831 |
| **444679+**  **3U4K** | **-7.3** | A:ARG125 | Alkyl | 5.07224 |
|  |  | A:ALA127 | Alkyl | 3.73998 |
|  |  | N:UNK1:C | Alkyl | 4.60661 |
|  |  | N:UNK1:C | Alkyl | 4.96755 |
|  |  | A:PHE124 | Pi-Alkyl | 5.28991 |
|  |  | A:TYR155 | Pi-Alkyl | 5.27346 |
|  |  | A:TYR155 | Pi-Alkyl | 5.44372 |
| **21118293+**  **3U4K** | **-7.3** | N:UNK1:H | Conventional Hydrogen Bond | 2.5134 |
|  |  | A:LYS78 | Alkyl | 4.23087 |
|  |  | A:ALA82 | Alkyl | 3.78894 |
|  |  | N:UNK1:C | Alkyl | 4.56921 |
|  |  | N:UNK1 | Pi-Alkyl | 5.04359 |
| **139585195+**  **3U4K** | **-7.3** | N:UNK1:H | Conventional Hydrogen Bond | 2.2498 |
|  |  | A:ALA51:CB | Pi-Sigma | 3.79878 |
|  |  | N:UNK1:C | Alkyl | 4.03187 |
|  |  | N:UNK1:C | Alkyl | 4.5373 |
|  |  | N:UNK1:C | Alkyl | 4.20956 |
|  |  | N:UNK1:C | Alkyl | 4.64492 |
|  |  | N:UNK1:C | Alkyl | 5.46154 |
|  |  | N:UNK1 | Pi-Alkyl | 5.07737 |
|  |  | N:UNK1 | Pi-Alkyl | 5.37001 |
|  |  | N:UNK1 | Pi-Alkyl | 4.43599 |
|  |  | N:UNK1 | Pi-Alkyl | 4.49534 |
|  |  | N:UNK1 | Pi-Alkyl | 5.27437 |
| **13892272+**  **3U4K** | **-7.3** | A:ARG125:HN | Conventional Hydrogen Bond | 1.98895 |
|  |  | A:TYR117 | Pi-Pi Stacked | 5.63388 |
|  |  | A:ALA108 | Alkyl | 4.37564 |
|  |  | A:ALA127 | Alkyl | 3.4561 |
|  |  | N:UNK1:C | Alkyl | 5.31637 |
| **108075+**  **3U4K** | **-7.3** | A:SER93:HN | Conventional Hydrogen Bond | 2.71064 |
|  |  | A:THR94:HN | Conventional Hydrogen Bond | 2.78367 |
|  |  | A:ILE99:HN | Conventional Hydrogen Bond | 2.4944 |
|  |  | A:GLY87:CA | Carbon Hydrogen Bond | 3.07714 |
|  |  | A:GLY87:CA | Carbon Hydrogen Bond | 3.73217 |
|  |  | A:SER93:CB | Carbon Hydrogen Bond | 3.12465 |
|  |  | A:ALA88:HN | Pi-Donor Hydrogen Bond | 3.19246 |
|  |  | A:ALA88:CB | Pi-Sigma | 3.6905 |
|  |  | N:UNK1:C | Alkyl | 4.02146 |
|  |  | N:UNK1 | Pi-Alkyl | 4.55038 |
| **10245773+**  **3U4K** | **-7.3** | A:ALA127:HN | Conventional Hydrogen Bond | 2.86327 |
|  |  | A:LEU126:CA | Carbon Hydrogen Bond | 3.75738 |
|  |  | N:UNK1:C | Alkyl | 4.88164 |
|  |  | N:UNK1:C | Alkyl | 4.50465 |
|  |  | N:UNK1:C | Alkyl | 4.65386 |
|  |  | N:UNK1:C | Alkyl | 4.89753 |
|  |  | N:UNK1:C | Alkyl | 4.18497 |
|  |  | N:UNK1 | Pi-Alkyl | 5.45381 |
|  |  | N:UNK1 | Pi-Alkyl | 5.26676 |
| **24879032+**  **3U4K** | **-7.2** | N:UNK1:H | Conventional Hydrogen Bond | 2.01081 |
|  |  | N:UNK1:H | Conventional Hydrogen Bond | 2.15613 |
|  |  | N:UNK1:C | Alkyl | 4.55178 |
|  |  | N:UNK1:C | Alkyl | 4.40072 |
|  |  | N:UNK1 | Pi-Alkyl | 5.06864 |
|  |  | N:UNK1 | Pi-Alkyl | 5.30919 |
| **10929061+**  **3U4K** | **-7.2** | A:ARG125:HN | Conventional Hydrogen Bond | 2.38422 |
|  |  | A:ARG125:HH22 | Conventional Hydrogen Bond | 2.51122 |
|  |  | N:UNK1:H | Conventional Hydrogen Bond | 2.31223 |
|  |  | A:PHE124 | Pi-Alkyl | 5.43429 |
| **54677878+**  **3U4K** | **-7.1** | A:SER75:HG | Conventional Hydrogen Bond | 2.16769 |
|  |  | A:TYR117:HH | Conventional Hydrogen Bond | 2.26752 |
|  |  | N:UNK1:H | Conventional Hydrogen Bond | 1.94044 |
|  |  | A:ILE109:CD | Pi-Sigma | 3.93248 |
|  |  | A:ALA108 | Alkyl | 3.90676 |
|  |  | A:ALA108 | Alkyl | 4.04037 |
|  |  | A:ALA108 | Alkyl | 4.11424 |
|  |  | A:ALA127 | Alkyl | 3.87244 |
| **38353925+**  **3U4K** | **-7.1** | A:ARG125:HH11 | Conventional Hydrogen Bond | 2.64546 |
|  |  | A:ALA108 | Alkyl | 4.33465 |
|  |  | N:UNK1:C | Alkyl | 5.07397 |
|  |  | A:PHE124 | Pi-Alkyl | 5.19762 |
| **3002143+**  **3U4K** | **-7.1** | A:THR119:HG1 | Conventional Hydrogen Bond | 2.59984 |
|  |  | N:UNK1:C | Alkyl | 5.44476 |
|  |  | A:TYR117 | Pi-Alkyl | 4.96635 |
|  |  | A:PHE124 | Pi-Alkyl | 4.84257 |
| **25163935+**  **3U4K** | **-7.1** | A:THR119:HG1 | Conventional Hydrogen Bond | 2.59984 |
|  |  | N:UNK1:C | Alkyl | 5.44476 |
|  |  | A:TYR117 | Pi-Alkyl | 4.96635 |
|  |  | A:PHE124 | Pi-Alkyl | 4.84257 |
| **25022540+**  **3U4K** | **-7.1** | A:TYR155:HH | Conventional Hydrogen Bond | 2.00676 |
|  |  | N:UNK1:C | Alkyl | 5.28195 |
|  |  | A:TYR117 | Pi-Alkyl | 4.95954 |
|  |  | A:PHE124 | Pi-Alkyl | 5.13422 |
|  |  | A:PHE124 | Pi-Alkyl | 5.36567 |
|  |  | A:TYR155 | Pi-Alkyl | 4.87336 |
| **16132327+**  **3U4K** | **-7.1** | A:ARG125:HH11 | Conventional Hydrogen Bond | 2.64546 |
|  |  | A:ALA108 | Alkyl | 4.33465 |
|  |  | N:UNK1:C | Alkyl | 5.07397 |
|  |  | A:PHE124 | Pi-Alkyl | 5.19762 |
| **159013642+**  **3U4K** | **-7.1** | A:GLY87:CA | Pi-Sigma | 3.71083 |
|  |  | A:VAL91:CG2 | Pi-Sigma | 3.82362 |
|  |  | A:VAL91:CG2 | Pi-Sigma | 3.83885 |
|  |  | A:THR138:CG2 | Pi-Sigma | 3.88665 |
|  |  | N:UNK1 | Pi-Pi T-shaped | 4.95824 |
|  |  | A:ALA88 | Alkyl | 5.10906 |
|  |  | N:UNK1 | Pi-Alkyl | 4.75955 |
|  |  | N:UNK1 | Pi-Alkyl | 5.43112 |
|  |  | N:UNK1 | Pi-Alkyl | 5.49115 |
| **139590669+**  **3U4K** | **-7.1** | A:THR119:HG1 | Conventional Hydrogen Bond | 2.03455 |
|  |  | A:ARG125:HH22 | Conventional Hydrogen Bond | 3.06926 |
|  |  | N:UNK1:H | Conventional Hydrogen Bond | 2.55482 |
|  |  | N:UNK1:H | Conventional Hydrogen Bond | 2.25082 |
|  |  | A:ILE109:CD | Pi-Sigma | 3.73908 |
|  |  | A:PHE124 | Pi-Alkyl | 5.41178 |
|  |  | N:UNK1 | Pi-Alkyl | 5.30028 |
| **139590668+**  **3U4K** | **-7.1** | A:THR119:HG1 | Conventional Hydrogen Bond | 2.05152 |
|  |  | A:ARG125:HH22 | Conventional Hydrogen Bond | 3.0595 |
|  |  | A:ILE109:CD | Pi-Sigma | 3.72404 |
|  |  | A:PHE124 | Pi-Alkyl | 5.39877 |
|  |  | N:UNK1 | Pi-Alkyl | 5.29878 |
| **139584550+**  **3U4K** | **-7.1** | A:ASN70:HD21 | Conventional Hydrogen Bond | 2.14714 |
|  |  | A:SER123:HN | Conventional Hydrogen Bond | 2.49767 |
|  |  | A:ARG125:HH22 | Conventional Hydrogen Bond | 2.64547 |
|  |  | A:TYR117 | Pi-Alkyl | 5.49895 |
| **10954384+**  **3U4K** | **-7.1** | A:ARG105:HH11 | Conventional Hydrogen Bond | 2.12363 |
|  |  | A:ARG105:HH21 | Conventional Hydrogen Bond | 1.96573 |
|  |  | A:ALA127:HN | Conventional Hydrogen Bond | 1.83946 |
|  |  | A:PHE124 | Pi-Pi Stacked | 4.25714 |
|  |  | N:UNK1 | Pi-Alkyl | 4.99232 |
|  |  | N:UNK1 | Pi-Alkyl | 5.14709 |
|  |  | N:UNK1 | Pi-Alkyl | 4.39424 |
| **101301307+**  **3U4K** | **-7.1** | A:ARG125:HH11 | Conventional Hydrogen Bond | 2.64546 |
|  |  | A:ALA108 | Alkyl | 4.33465 |
|  |  | N:UNK1:C | Alkyl | 5.07397 |
|  |  | A:PHE124 | Pi-Alkyl | 5.19762 |
| **9996305+**  **3U4K** | **-7** | N:UNK1:H | Conventional Hydrogen Bond | 1.92017 |
|  |  | N:UNK1 | Pi-Alkyl | 5.39231 |
|  |  | N:UNK1 | Pi-Alkyl | 3.90735 |
|  |  | N:UNK1 | Pi-Alkyl | 4.79324 |
|  |  | N:UNK1 | Pi-Alkyl | 3.84653 |
|  |  | N:UNK1 | Pi-Alkyl | 5.33442 |
| **54677878+**  **3U4K** | **-7** | A:SER75:HG | Conventional Hydrogen Bond | 2.16769 |
|  |  | A:TYR117:HH | Conventional Hydrogen Bond | 2.26752 |
|  |  | N:UNK1:H | Conventional Hydrogen Bond | 1.94044 |
|  |  | A:ILE109:CD | Pi-Sigma | 3.93248 |
|  |  | A:ALA108 | Alkyl | 3.90676 |
|  |  | A:ALA108 | Alkyl | 4.04037 |
|  |  | A:ALA108 | Alkyl | 4.11424 |
|  |  | A:ALA127 | Alkyl | 3.87244 |
| **5368914+**  **3U4K** | **-7** | A:ARG125:HN | Conventional Hydrogen Bond | 2.2847 |
|  |  | N:UNK1:H | Conventional Hydrogen Bond | 2.18927 |
|  |  | N:UNK1:C | Alkyl | 4.22868 |
|  |  | N:UNK1:C | Alkyl | 3.94931 |
|  |  | N:UNK1:C | Alkyl | 5.083 |
|  |  | A:PHE124 | Pi-Alkyl | 4.29892 |
|  |  | A:PHE124 | Pi-Alkyl | 5.20374 |
|  |  | A:PHE124 | Pi-Alkyl | 4.68446 |
| **38353925+**  **3U4K** | **-7** | A:ARG125:HN | Conventional Hydrogen Bond | 2.2847 |
|  |  | N:UNK1:H | Conventional Hydrogen Bond | 2.18927 |
|  |  | N:UNK1:C | Alkyl | 4.22868 |
|  |  | N:UNK1:C | Alkyl | 3.94931 |
|  |  | N:UNK1:C | Alkyl | 5.083 |
|  |  | A:PHE124 | Pi-Alkyl | 4.29892 |
|  |  | A:PHE124 | Pi-Alkyl | 5.20374 |
|  |  | A:PHE124 | Pi-Alkyl | 4.68446 |
| **36314+**  **3U4K** | **-7** | A:TYR117:HH | Conventional Hydrogen Bond | 1.90552 |
|  |  | A:THR119:HG1 | Conventional Hydrogen Bond | 2.17113 |
|  |  | A:THR119:HG1 | Conventional Hydrogen Bond | 2.42075 |
|  |  | A:SER122:HN | Conventional Hydrogen Bond | 2.7295 |
|  |  | A:SER123:HN | Conventional Hydrogen Bond | 2.28405 |
|  |  | A:ARG125:HN | Conventional Hydrogen Bond | 2.37393 |
|  |  | A:ARG125:HE | Conventional Hydrogen Bond | 3.08852 |
|  |  | A:ARG125:HH21 | Conventional Hydrogen Bond | 2.44436 |
|  |  | A:ARG125:HH22 | Conventional Hydrogen Bond | 2.07652 |
|  |  | A:ARG125:HH22 | Conventional Hydrogen Bond | 2.34438 |
|  |  | A:ARG125:HH22 | Conventional Hydrogen Bond | 2.43856 |
|  |  | N:UNK1 | Pi-Pi Stacked | 3.75312 |
|  |  | A:TYR117 | Pi-Pi T-shaped | 5.88321 |
|  |  | N:UNK1 | Pi-Alkyl | 4.33303 |
|  |  | N:UNK1 | Pi-Alkyl | 5.29957 |
| **15939768+**  **3U4K** | **-7** | A:ARG125 | Alkyl | 5.18781 |
|  |  | N:UNK1:C | Alkyl | 4.59106 |
|  |  | A:TYR117 | Pi-Alkyl | 5.2573 |
| **139585251+**  **3U4K** | **-7** | A:TYR117:HH | Conventional Hydrogen Bond | 2.09362 |
|  |  | A:THR119:HG1 | Conventional Hydrogen Bond | 2.31122 |
|  |  | A:ARG125:HN | Conventional Hydrogen Bond | 2.44312 |
|  |  | A:ARG125:HH22 | Conventional Hydrogen Bond | 2.27716 |
|  |  | A:PHE124:CA | Carbon Hydrogen Bond | 3.3266 |
|  |  | A:ARG125:NH2 | Pi-Cation | 3.8412 |
|  |  | A:TYR117 | Pi-Pi T-shaped | 5.6141 |
|  |  | N:UNK1 | Pi-Alkyl | 4.52372 |
|  |  | N:UNK1 | Pi-Alkyl | 5.40207 |
| **13858913+**  **3U4K** | **-7** | A:TYR117:HH | Conventional Hydrogen Bond | 2.21989 |
|  |  | A:THR119:HG1 | Conventional Hydrogen Bond | 2.3246 |
|  |  | A:ARG125:HN | Conventional Hydrogen Bond | 1.82342 |
|  |  | N:UNK1:H | Conventional Hydrogen Bond | 2.21924 |
|  |  | A:TYR155:HH | Pi-Donor Hydrogen Bond | 3.0697 |
| **10954384+**  **3U4K** | **-7** | A:ARG105:HH11 | Conventional Hydrogen Bond | 2.12363 |
|  |  | A:ARG105:HH21 | Conventional Hydrogen Bond | 1.96573 |
|  |  | A:ALA127:HN | Conventional Hydrogen Bond | 1.83946 |
|  |  | A:PHE124 | Pi-Pi Stacked | 4.25714 |
|  |  | N:UNK1 | Pi-Alkyl | 4.99232 |
|  |  | N:UNK1 | Pi-Alkyl | 5.14709 |
|  |  | N:UNK1 | Pi-Alkyl | 4.39424 |
| **73431+**  **3U4K** | **-6.9** | N:UNK1:H | Conventional Hydrogen Bond | 2.33677 |
|  |  | N:UNK1:H | Conventional Hydrogen Bond | 2.3169 |
|  |  | N:UNK1:H | Conventional Hydrogen Bond | 1.95586 |
|  |  | N:UNK1:H | Conventional Hydrogen Bond | 2.35597 |
|  |  | N:UNK1:H | Conventional Hydrogen Bond | 2.56063 |
|  |  | A:ASP115:CA | Carbon Hydrogen Bond | 3.47628 |
|  |  | N:UNK1:C | Alkyl | 4.39555 |
|  |  | N:UNK1 | Pi-Alkyl | 4.50897 |
|  |  | N:UNK1 | Pi-Alkyl | 4.46905 |
| **5280793+**  **3U4K** | **-6.9** | A:ARG125 | Alkyl | 5.18781 |
|  |  | N:UNK1:C | Alkyl | 4.59106 |
|  |  | A:TYR117 | Pi-Alkyl | 5.2573 |
| **44178748+**  **3U4K** | **-6.9** | A:THR119:HG1 | Conventional Hydrogen Bond | 2.22281 |
|  |  | A:THR119:HG1 | Conventional Hydrogen Bond | 2.50379 |
|  |  | A:ARG125:HN | Conventional Hydrogen Bond | 2.39427 |
|  |  | A:ARG125:HE | Conventional Hydrogen Bond | 2.81441 |
|  |  | A:ARG125:HH22 | Conventional Hydrogen Bond | 2.32687 |
|  |  | N:UNK1:H | Conventional Hydrogen Bond | 2.6605 |
|  |  | N:UNK1:H | Conventional Hydrogen Bond | 2.27309 |
|  |  | N:UNK1:H | Conventional Hydrogen Bond | 3.02366 |
|  |  | N:UNK1:H | Conventional Hydrogen Bond | 2.79259 |
|  |  | N:UNK1:H | Conventional Hydrogen Bond | 2.39173 |
|  |  | N:UNK1:H | Conventional Hydrogen Bond | 3.03338 |
|  |  | N:UNK1:H | Conventional Hydrogen Bond | 2.31218 |
|  |  | A:ILE109:CA | Carbon Hydrogen Bond | 3.38463 |
|  |  | N:UNK1:C | Carbon Hydrogen Bond | 3.49837 |
|  |  | A:PHE124 | Pi-Alkyl | 4.49047 |
| **3043792+**  **3U4K** | **-6.9** | A:SER75:HG | Conventional Hydrogen Bond | 1.90283 |
|  |  | A:ARG125:HE | Conventional Hydrogen Bond | 2.38281 |
|  |  | A:ARG125:HH22 | Conventional Hydrogen Bond | 2.59366 |
|  |  | N:UNK1:HN | Conventional Hydrogen Bond | 2.88794 |
|  |  | A:TYR155:HH | Pi-Donor Hydrogen Bond | 3.16235 |
|  |  | A:TYR117 | Pi-Pi T-shaped | 5.5829 |
| **24853973+**  **3U4K** | **-6.9** | N:UNK1:H | Conventional Hydrogen Bond | 2.19898 |
|  |  | A:LYS106:CD | Pi-Sigma | 3.90885 |
|  |  | A:THR132:CG2 | Pi-Sigma | 3.6523 |
|  |  | N:UNK1 | Pi-Alkyl | 5.45993 |
| **162642187+**  **3U4K** | **-6.9** | A:ARG105:HH21 | Conventional Hydrogen Bond | 2.30399 |
|  |  | A:ALA127:HN | Conventional Hydrogen Bond | 1.95737 |
|  |  | A:TYR155:HH | Conventional Hydrogen Bond | 2.25981 |
|  |  | N:UNK1:H | Conventional Hydrogen Bond | 2.24028 |
| **11336960+**  **3U4K** | **-6.9** | A:ARG125 | Alkyl | 5.18781 |
|  |  | N:UNK1:C | Alkyl | 4.59106 |
|  |  | A:TYR117 | Pi-Alkyl | 5.2573 |
| **139589737+**  **3U4K** | **-6.9** | N:UNK1:H | Conventional Hydrogen Bond | 2.08033 |
|  |  | N:UNK1:H | Conventional Hydrogen Bond | 2.0081 |
|  |  | N:UNK1 | Alkyl | 5.4361 |
|  |  | N:UNK1:C | Alkyl | 3.70262 |
|  |  | A:TYR117 | Pi-Alkyl | 5.15517 |
|  |  | A:PHE124 | Pi-Alkyl | 5.42179 |
| **125114671+**  **3U4K** | **-6.9** | A:LYS78 | Alkyl | 5.36388 |
|  |  | A:PRO114 | Alkyl | 5.19215 |
| **54691338+**  **3U4K** | **-6.8** | A:GLY121:CA | Carbon Hydrogen Bond | 3.34857 |
|  |  | N:UNK1:C | Alkyl | 4.97403 |
|  |  | N:UNK1:C | Alkyl | 5.33014 |
|  |  | N:UNK1:C | Alkyl | 4.53651 |
|  |  | N:UNK1:C | Alkyl | 4.44317 |
|  |  | N:UNK1:C | Alkyl | 4.10761 |
|  |  | A:TYR117 | Pi-Alkyl | 5.04744 |
|  |  | A:PHE124 | Pi-Alkyl | 4.82652 |
|  |  | A:PHE124 | Pi-Alkyl | 4.48625 |
|  |  | A:PHE124 | Pi-Alkyl | 4.35839 |
|  |  | A:TYR155 | Pi-Alkyl | 5.31657 |
| **54682463+**  **3U4K** | **-6.8** | A:SER122:HN | Conventional Hydrogen Bond | 1.82994 |
|  |  | A:SER123:HN | Conventional Hydrogen Bond | 2.30439 |
|  |  | N:UNK1:H | Conventional Hydrogen Bond | 2.53062 |
|  |  | A:ARG125 | Alkyl | 4.32899 |
|  |  | N:UNK1:C | Alkyl | 5.36831 |
|  |  | N:UNK1:C | Alkyl | 4.35025 |
|  |  | N:UNK1 | Pi-Alkyl | 5.10747 |
| **54680181+**  **3U4K** | **-6.8** | N:UNK1:H | Conventional Hydrogen Bond | 2.43839 |
|  |  | A:ILE109:CD | Pi-Sigma | 3.96185 |
|  |  | A:ALA108 | Alkyl | 3.83758 |
|  |  | A:ALA108 | Alkyl | 3.67766 |
|  |  | A:ALA127 | Alkyl | 4.13751 |
|  |  | N:UNK1 | Pi-Alkyl | 5.45912 |
| **443788+**  **3U4K** | **-6.8** | A:THR119:HG1 | Conventional Hydrogen Bond | 3.02667 |
|  |  | A:TYR117 | Pi-Pi T-shaped | 5.57371 |
|  |  | A:ALA127 | Alkyl | 3.77728 |
|  |  | N:UNK1:C | Alkyl | 4.36901 |
|  |  | N:UNK1:C | Alkyl | 4.79411 |
|  |  | N:UNK1 | Pi-Alkyl | 5.43464 |
| **44254253+**  **3U4K** | **-6.8** | A:THR138:HG1 | Conventional Hydrogen Bond | 2.93301 |
|  |  | N:UNK1:H | Conventional Hydrogen Bond | 2.25324 |
|  |  | A:SER93:CB | Carbon Hydrogen Bond | 3.36203 |
|  |  | A:SER93:HG | Pi-Donor Hydrogen Bond | 2.8102 |
|  |  | A:THR138:CG2 | Pi-Sigma | 3.48054 |
|  |  | A:VAL91:C,O;TYR92:N | Amide-Pi Stacked | 5.11504 |
|  |  | A:ALA88 | Alkyl | 3.99872 |
|  |  | A:ALA88 | Alkyl | 3.78611 |
|  |  | N:UNK1:C | Alkyl | 4.55967 |
|  |  | N:UNK1:C | Alkyl | 4.32 |
|  |  | N:UNK1:C | Alkyl | 4.97076 |
|  |  | N:UNK1:C | Alkyl | 5.00307 |
|  |  | N:UNK1:C | Alkyl | 3.87114 |
|  |  | N:UNK1 | Pi-Alkyl | 5.09699 |
| **44254250+**  **3U4K** | **-6.8** | A:ARG105:HH11 | Conventional Hydrogen Bond | 2.7633 |
|  |  | A:THR119:HG1 | Conventional Hydrogen Bond | 2.48472 |
|  |  | N:UNK1:H | Conventional Hydrogen Bond | 2.34501 |
|  |  | N:UNK1:C | Alkyl | 4.8004 |
|  |  | N:UNK1:C | Alkyl | 4.60193 |
|  |  | N:UNK1:C | Alkyl | 5.23506 |
|  |  | A:TYR117 | Pi-Alkyl | 5.30324 |
|  |  | A:PHE124 | Pi-Alkyl | 4.78569 |
|  |  | A:PHE124 | Pi-Alkyl | 5.37402 |
| **44178749+**  **3U4K** | **-6.8** | A:THR120:HN | Conventional Hydrogen Bond | 1.97713 |
|  |  | A:ARG125:HN | Conventional Hydrogen Bond | 2.13552 |
|  |  | A:ALA127:HN | Conventional Hydrogen Bond | 2.92892 |
|  |  | N:UNK1:H | Conventional Hydrogen Bond | 2.45552 |
|  |  | N:UNK1:H | Conventional Hydrogen Bond | 2.25243 |
|  |  | N:UNK1:H | Conventional Hydrogen Bond | 2.34502 |
|  |  | N:UNK1:H | Conventional Hydrogen Bond | 2.66541 |
|  |  | N:UNK1:H | Conventional Hydrogen Bond | 2.54103 |
|  |  | N:UNK1:H | Conventional Hydrogen Bond | 2.03371 |
|  |  | N:UNK1:H | Conventional Hydrogen Bond | 3.05429 |
| **169680+**  **3U4K** | **-6.8** | A:GLY83:HN | Conventional Hydrogen Bond | 2.44116 |
|  |  | N:UNK1:H | Conventional Hydrogen Bond | 2.71779 |
|  |  | N:UNK1:H | Conventional Hydrogen Bond | 1.9063 |
|  |  | N:UNK1:H | Conventional Hydrogen Bond | 3.00218 |
|  |  | N:UNK1:H | Conventional Hydrogen Bond | 2.18778 |
|  |  | N:UNK1:H | Conventional Hydrogen Bond | 1.98572 |
|  |  | A:ALA82 | Alkyl | 3.89832 |
|  |  | N:UNK1 | Pi-Alkyl | 5.09481 |
|  |  | N:UNK1 | Pi-Alkyl | 4.51298 |
| **11336960+**  **3U4K** | **-6.8** | A:THR119:HG1 | Conventional Hydrogen Bond | 2.35058 |
|  |  | A:ARG125:HE | Conventional Hydrogen Bond | 3.09694 |
|  |  | N:UNK1:H | Conventional Hydrogen Bond | 2.1715 |
|  |  | A:PHE124 | Pi-Pi T-shaped | 5.43461 |
| **10474528+**  **3U4K** | **-6.8** | A:TYR117:HH | Conventional Hydrogen Bond | 1.92064 |
|  |  | A:THR119:HG1 | Conventional Hydrogen Bond | 2.52783 |
|  |  | A:ARG125:HH21 | Conventional Hydrogen Bond | 2.91743 |
|  |  | A:ARG125:HH21 | Conventional Hydrogen Bond | 2.27414 |
|  |  | N:UNK1:H | Conventional Hydrogen Bond | 2.20176 |
|  |  | N:UNK1:H | Conventional Hydrogen Bond | 2.38501 |
|  |  | N:UNK1:H | Conventional Hydrogen Bond | 1.77787 |
|  |  | A:ILE109:CD | Pi-Sigma | 3.90862 |
|  |  | N:UNK1 | Pi-Alkyl | 5.13238 |
|  |  | N:UNK1 | Pi-Alkyl | 4.34708 |
|  |  | N:UNK1 | Pi-Alkyl | 4.1216 |
|  |  | N:UNK1 | Pi-Alkyl | 3.96556 |
|  |  | N:UNK1 | Pi-Alkyl | 4.54338 |
| **73431+**  **3U4K** | **-6.7** | N:UNK1:H | Conventional Hydrogen Bond | 1.98821 |
|  |  | N:UNK1:H | Conventional Hydrogen Bond | 2.39213 |
|  |  | N:UNK1:H | Conventional Hydrogen Bond | 1.74957 |
|  |  | N:UNK1:H | Conventional Hydrogen Bond | 2.54287 |
|  |  | N:UNK1:H | Conventional Hydrogen Bond | 2.29994 |
|  |  | N:UNK1:H | Conventional Hydrogen Bond | 1.94834 |
|  |  | A:ASP115:CA | Carbon Hydrogen Bond | 3.48661 |
|  |  | N:UNK1:C | Alkyl | 4.3516 |
|  |  | N:UNK1 | Pi-Alkyl | 4.50527 |
|  |  | N:UNK1 | Pi-Alkyl | 4.50881 |
| **71680656+**  **3U4K** | **-6.7** | A:GLY36:HN3 | Conventional Hydrogen Bond | 2.97075 |
|  |  | N:UNK1:H | Conventional Hydrogen Bond | 2.80403 |
|  |  | A:ALA51 | Alkyl | 4.46222 |
|  |  | N:UNK1 | Pi-Alkyl | 5.48353 |
|  |  | N:UNK1 | Pi-Alkyl | 4.49538 |
|  |  | N:UNK1 | Pi-Alkyl | 4.35554 |
|  |  | N:UNK1 | Pi-Alkyl | 5.38613 |
| **54718903+**  **3U4K** | **-6.7** | A:ARG125:HE | Conventional Hydrogen Bond | 2.30356 |
|  |  | N:UNK1:H | Conventional Hydrogen Bond | 2.65072 |
|  |  | N:UNK1:H | Conventional Hydrogen Bond | 2.5183 |
|  |  | A:SER123:CB | Carbon Hydrogen Bond | 3.44358 |
|  |  | A:ALA108 | Alkyl | 4.41742 |
|  |  | A:ALA127 | Alkyl | 3.73522 |
|  |  | A:TYR117 | Pi-Alkyl | 5.36647 |
|  |  | A:PHE124 | Pi-Alkyl | 5.42651 |
|  |  | A:TYR155 | Pi-Alkyl | 5.28496 |
| **54691340+**  **3U4K** | **-6.7** | A:GLY121:CA | Carbon Hydrogen Bond | 3.69326 |
|  |  | A:ARG125 | Alkyl | 4.90232 |
|  |  | N:UNK1:C | Alkyl | 5.30541 |
|  |  | N:UNK1:C | Alkyl | 4.56516 |
|  |  | N:UNK1:C | Alkyl | 4.7725 |
|  |  | N:UNK1:C | Alkyl | 4.77732 |
|  |  | N:UNK1:C | Alkyl | 4.62711 |
|  |  | A:TYR117 | Pi-Alkyl | 5.07697 |
|  |  | A:TYR117 | Pi-Alkyl | 5.48456 |
|  |  | A:PHE124 | Pi-Alkyl | 5.0983 |
|  |  | A:PHE124 | Pi-Alkyl | 4.50795 |
|  |  | A:PHE124 | Pi-Alkyl | 4.51443 |
|  |  | A:TYR155 | Pi-Alkyl | 5.4321 |
| **54680181+**  **3U4K** | **-6.7** | N:UNK1:H | Conventional Hydrogen Bond | 2.43839 |
|  |  | A:ILE109:CD | Pi-Sigma | 3.96185 |
|  |  | A:ALA108 | Alkyl | 3.83758 |
|  |  | A:ALA108 | Alkyl | 3.67766 |
|  |  | A:ALA127 | Alkyl | 4.13751 |
|  |  | N:UNK1 | Pi-Alkyl | 5.45912 |
| **5280389+**  **3U4K** | **-6.7** | N:UNK1:H | Conventional Hydrogen Bond | 1.94128 |
|  |  | A:ASP134:OD1 | Pi-Anion | 4.63181 |
|  |  | A:ASP134:OD1 | Pi-Anion | 3.97962 |
|  |  | A:THR132:CG2 | Pi-Sigma | 3.90819 |
|  |  | A:THR132:CG2 | Pi-Sigma | 3.75491 |
|  |  | N:UNK1:C | Alkyl | 4.57673 |
|  |  | N:UNK1 | Pi-Alkyl | 5.02119 |
|  |  | N:UNK1 | Pi-Alkyl | 5.41425 |
| **44445553+**  **3U4K** | **-6.7** | N:UNK1:C | Alkyl | 4.62261 |
|  |  | N:UNK1:C | Alkyl | 4.4817 |
|  |  | N:UNK1:C | Alkyl | 5.01149 |
|  |  | N:UNK1:C | Alkyl | 4.92119 |
|  |  | N:UNK1:C | Alkyl | 5.19404 |
|  |  | A:PHE124 | Pi-Alkyl | 4.86301 |
|  |  | A:TYR155 | Pi-Alkyl | 5.04119 |
| **23582930+**  **3U4K** | **-6.7** | A:TYR117:HH | Conventional Hydrogen Bond | 2.36158 |
|  |  | A:THR119:HG1 | Conventional Hydrogen Bond | 2.27655 |
|  |  | A:ARG125:HH21 | Conventional Hydrogen Bond | 2.30584 |
|  |  | N:UNK1:H | Conventional Hydrogen Bond | 2.15681 |
|  |  | N:UNK1:H | Conventional Hydrogen Bond | 2.74027 |
|  |  | A:ALA127:CB | Pi-Sigma | 3.88666 |
|  |  | A:ALA127 | Alkyl | 3.72558 |
|  |  | N:UNK1:C | Alkyl | 4.30458 |
|  |  | N:UNK1:C | Alkyl | 5.1877 |
|  |  | N:UNK1 | Pi-Alkyl | 4.11122 |
| **21578704+**  **3U4K** | **-6.7** | A:THR119:HG1 | Conventional Hydrogen Bond | 2.35058 |
|  |  | A:ARG125:HE | Conventional Hydrogen Bond | 3.09694 |
|  |  | N:UNK1:H | Conventional Hydrogen Bond | 2.1715 |
|  |  | A:PHE124 | Pi-Pi T-shaped | 5.43461 |
| **169680+**  **3U4K** | **-6.7** | N:UNK1:H | Conventional Hydrogen Bond | 2.86111 |
|  |  | N:UNK1:H | Conventional Hydrogen Bond | 2.59082 |
|  |  | N:UNK1:H | Conventional Hydrogen Bond | 2.15281 |
|  |  | N:UNK1:H | Conventional Hydrogen Bond | 2.29396 |
|  |  | A:ALA82 | Alkyl | 3.82727 |
|  |  | N:UNK1 | Pi-Alkyl | 5.06761 |
|  |  | N:UNK1 | Pi-Alkyl | 4.55489 |
| **12082078+**  **3U4K** | **-6.7** | A:ARG125:HH21 | Conventional Hydrogen Bond | 2.62037 |
|  |  | A:ARG125:HH22 | Conventional Hydrogen Bond | 2.65551 |
|  |  | A:ALA108 | Alkyl | 3.7787 |
|  |  | N:UNK1:C | Alkyl | 4.4807 |
| **10286+**  **3U4K** | **-6.7** | A:TYR155:HH | Pi-Donor Hydrogen Bond | 3.07926 |
|  |  | A:PHE124 | Pi-Pi Stacked | 4.39401 |
|  |  | A:PHE124 | Pi-Pi Stacked | 5.9222 |
|  |  | A:TYR155 | Pi-Pi T-shaped | 5.71815 |
|  |  | N:UNK1 | Pi-Alkyl | 5.13654 |
|  |  | N:UNK1 | Pi-Alkyl | 5.48255 |
|  |  | N:UNK1 | Pi-Alkyl | 4.60241 |
| **72945800+**  **3U4K** | **-6.6** | A:ARG125:HN | Conventional Hydrogen Bond | 2.26122 |
|  |  | A:ARG125:HH11 | Conventional Hydrogen Bond | 3.01899 |
|  |  | A:ARG125:HH21 | Conventional Hydrogen Bond | 2.95597 |
|  |  | A:ALA127:HN | Conventional Hydrogen Bond | 2.77273 |
|  |  | N:UNK1:H | Conventional Hydrogen Bond | 2.17572 |
|  |  | A:PHE124:CA | Carbon Hydrogen Bond | 3.3076 |
|  |  | N:UNK1:C | Pi-Sigma | 3.85147 |
|  |  | N:UNK1:C | Alkyl | 4.01861 |
|  |  | N:UNK1:C | Alkyl | 4.44761 |
|  |  | N:UNK1:C | Alkyl | 3.83018 |
| **54691338+**  **3U4K** | **-6.6** | A:GLY121:CA | Carbon Hydrogen Bond | 3.34857 |
|  |  | N:UNK1:C | Alkyl | 4.97403 |
|  |  | N:UNK1:C | Alkyl | 5.33014 |
|  |  | N:UNK1:C | Alkyl | 4.53651 |
|  |  | N:UNK1:C | Alkyl | 4.44317 |
|  |  | N:UNK1:C | Alkyl | 4.10761 |
|  |  | A:TYR117 | Pi-Alkyl | 5.04744 |
|  |  | A:PHE124 | Pi-Alkyl | 4.82652 |
|  |  | A:PHE124 | Pi-Alkyl | 4.48625 |
|  |  | A:PHE124 | Pi-Alkyl | 4.35839 |
|  |  | A:TYR155 | Pi-Alkyl | 5.31657 |
| **53483974+**  **3U4K** | **-6.6** | A:ARG125:HH22 | Conventional Hydrogen Bond | 2.85364 |
|  |  | N:UNK1:C | Carbon Hydrogen Bond | 3.63591 |
|  |  | N:UNK1:C | Pi-Sigma | 3.89379 |
|  |  | N:UNK1:C | Alkyl | 4.07419 |
|  |  | N:UNK1:C | Alkyl | 4.00475 |
|  |  | N:UNK1:C | Alkyl | 5.09628 |
|  |  | A:PHE124 | Pi-Alkyl | 5.28599 |
|  |  | A:PHE124 | Pi-Alkyl | 4.55346 |
|  |  | N:UNK1 | Pi-Alkyl | 4.78002 |
| **44564016+**  **3U4K** | **-6.6** | A:THR119:HG1 | Conventional Hydrogen Bond | 2.06606 |
|  |  | A:SER122:HN | Conventional Hydrogen Bond | 2.29482 |
|  |  | A:SER123:HN | Conventional Hydrogen Bond | 2.75721 |
|  |  | A:ALA127 | Alkyl | 4.19627 |
|  |  | N:UNK1:C | Alkyl | 3.61973 |
|  |  | N:UNK1:C | Alkyl | 4.83768 |
|  |  | N:UNK1:C | Alkyl | 4.12244 |
|  |  | N:UNK1:C | Alkyl | 4.47717 |
|  |  | N:UNK1:C | Alkyl | 4.86484 |
|  |  | N:UNK1:C | Alkyl | 4.85441 |
|  |  | A:TYR117 | Pi-Alkyl | 4.87135 |
| **3043792+**  **3U4K** | **-6.6** | A:SER75:HG | Conventional Hydrogen Bond | 1.90283 |
|  |  | A:ARG125:HE | Conventional Hydrogen Bond | 2.38281 |
|  |  | A:ARG125:HH22 | Conventional Hydrogen Bond | 2.59366 |
|  |  | N:UNK1:HN | Conventional Hydrogen Bond | 2.88794 |
|  |  | A:TYR155:HH | Pi-Donor Hydrogen Bond | 3.16235 |
|  |  | A:TYR117 | Pi-Pi T-shaped | 5.5829 |
| **25022541+**  **3U4K** | **-6.6** | A:ARG105:HH21 | Conventional Hydrogen Bond | 2.34424 |
|  |  | A:ALA127:HN | Conventional Hydrogen Bond | 2.06451 |
|  |  | A:TYR117 | Pi-Alkyl | 4.99204 |
| **139588514+**  **3U4K** | **-6.6** | A:TYR117:HH | Conventional Hydrogen Bond | 2.32422 |
|  |  | A:THR119:HG1 | Conventional Hydrogen Bond | 2.3917 |
|  |  | A:THR120:HN | Conventional Hydrogen Bond | 2.22565 |
|  |  | A:THR120:HG1 | Conventional Hydrogen Bond | 2.60829 |
|  |  | A:ARG125:HN | Conventional Hydrogen Bond | 2.39909 |
|  |  | A:ARG125:HE | Conventional Hydrogen Bond | 2.85515 |
|  |  | N:UNK1:H | Conventional Hydrogen Bond | 2.23534 |
|  |  | N:UNK1:H | Conventional Hydrogen Bond | 3.04205 |
|  |  | N:UNK1:H | Conventional Hydrogen Bond | 2.15688 |
|  |  | N:UNK1:H | Conventional Hydrogen Bond | 2.64384 |
|  |  | N:UNK1:H | Conventional Hydrogen Bond | 3.07386 |
|  |  | N:UNK1:H | Conventional Hydrogen Bond | 2.12249 |
|  |  | A:GLY121:CA | Carbon Hydrogen Bond | 3.42736 |
|  |  | N:UNK1:C | Carbon Hydrogen Bond | 3.14098 |
|  |  | A:PHE124 | Pi-Alkyl | 4.93405 |
| **12082078+**  **3U4K** | **-6.6** | A:ARG125:HH21 | Conventional Hydrogen Bond | 2.62037 |
|  |  | A:ARG125:HH22 | Conventional Hydrogen Bond | 2.65551 |
|  |  | A:ALA108 | Alkyl | 3.7787 |
|  |  | N:UNK1:C | Alkyl | 4.4807 |
| **54707854+**  **3U4K** | **-6.5** | N:UNK1:H | Conventional Hydrogen Bond | 1.81335 |
|  |  | N:UNK1:H | Conventional Hydrogen Bond | 2.02215 |
|  |  | A:ILE109:CD | Pi-Sigma | 3.66817 |
|  |  | A:ALA108 | Alkyl | 3.87003 |
|  |  | A:ALA108 | Alkyl | 4.05916 |
|  |  | A:ALA127 | Alkyl | 4.01336 |
| **42609854+**  **3U4K** | **-6.5** | A:TYR117:HH | Conventional Hydrogen Bond | 2.80529 |
|  |  | A:PHE124:CA | Carbon Hydrogen Bond | 3.35293 |
|  |  | A:ALA108 | Alkyl | 3.74586 |
|  |  | A:ALA127 | Alkyl | 3.75775 |
|  |  | N:UNK1:C | Alkyl | 5.11469 |
|  |  | N:UNK1:C | Alkyl | 4.92105 |
|  |  | N:UNK1:C | Alkyl | 4.97174 |
|  |  | A:PHE124 | Pi-Alkyl | 5.28989 |
|  |  | A:TYR155 | Pi-Alkyl | 5.11677 |
| **20997+**  **3U4K** | **-6.5** | N:UNK1:H | Conventional Hydrogen Bond | 2.51845 |
|  |  | N:UNK1:C | Carbon Hydrogen Bond | 3.43334 |
|  |  | N:UNK1 | Pi-Alkyl | 5.37783 |
|  |  | N:UNK1 | Pi-Alkyl | 4.47349 |
|  |  | N:UNK1 | Pi-Alkyl | 5.14479 |
| **186907+**  **3U4K** | **-6.5** | N:UNK1:C | Carbon Hydrogen Bond | 3.72034 |
|  |  | A:THR132:CG2 | Pi-Sigma | 3.73978 |
|  |  | A:LYS106 | Alkyl | 4.528 |
|  |  | A:ILE112 | Alkyl | 5.43791 |
|  |  | N:UNK1 | Pi-Alkyl | 5.06044 |
| **179521+**  **3U4K** | **-6.5** | A:GLN86:HE21 | Conventional Hydrogen Bond | 2.25355 |
|  |  | N:UNK1:H | Conventional Hydrogen Bond | 1.88095 |
|  |  | N:UNK1:C | Alkyl | 3.79923 |
|  |  | N:UNK1:C | Alkyl | 4.56029 |
|  |  | N:UNK1 | Pi-Alkyl | 4.43618 |
|  |  | N:UNK1 | Pi-Alkyl | 3.95254 |
| **139589963+**  **3U4K** | **-6.5** | A:ARG125:HN | Conventional Hydrogen Bond | 2.59394 |
|  |  | A:ARG125:HE | Conventional Hydrogen Bond | 2.57229 |
|  |  | N:UNK1 | Alkyl | 5.18585 |
|  |  | A:TYR117 | Pi-Alkyl | 5.28456 |
|  |  | A:PHE124 | Pi-Alkyl | 4.74271 |
|  |  | A:PHE124 | Pi-Alkyl | 4.82031 |
| **139589759+**  **3U4K** | **-6.5** | A:ALA88:HN | Pi-Donor Hydrogen Bond | 3.19749 |
|  |  | A:GLY87:CA | Pi-Sigma | 3.87618 |
|  |  | A:THR138:CG2 | Pi-Sigma | 3.98711 |
|  |  | A:ALA88 | Alkyl | 5.20715 |
|  |  | N:UNK1 | Pi-Alkyl | 5.15024 |
|  |  | N:UNK1 | Pi-Alkyl | 5.21796 |
| **115252+**  **3U4K** | **-6.5** | A:THR118:HG1 | Conventional Hydrogen Bond | 2.61251 |
|  |  | N:UNK1:H | Conventional Hydrogen Bond | 1.82041 |
|  |  | A:TYR117 | Pi-Pi T-shaped | 4.71093 |
|  |  | N:UNK1 | Pi-Pi T-shaped | 4.88955 |
|  |  | A:ALA77 | Alkyl | 4.01225 |
|  |  | N:UNK1:C | Alkyl | 4.75322 |
|  |  | A:TYR117 | Pi-Alkyl | 5.04926 |
|  |  | A:TYR117 | Pi-Alkyl | 5.07393 |
|  |  | A:TYR155 | Pi-Alkyl | 4.89362 |
| **10369606+**  **3U4K** | **-6.5** | N:UNK1:C | Carbon Hydrogen Bond | 3.72034 |
|  |  | A:THR132:CG2 | Pi-Sigma | 3.73978 |
|  |  | A:LYS106 | Alkyl | 4.528 |
|  |  | A:ILE112 | Alkyl | 5.43791 |
|  |  | N:UNK1 | Pi-Alkyl | 5.06044 |
| **10286097+**  **3U4K** | **-6.5** | A:GLY36:HN3 | Conventional Hydrogen Bond | 2.83895 |
|  |  | A:GLY36:HN2 | Conventional Hydrogen Bond | 2.9217 |
|  |  | N:UNK1:H | Conventional Hydrogen Bond | 2.6497 |
|  |  | N:UNK1:H | Conventional Hydrogen Bond | 2.11765 |
|  |  | N:UNK1:H | Conventional Hydrogen Bond | 2.30632 |
|  |  | N:UNK1:H | Conventional Hydrogen Bond | 2.4382 |
|  |  | N:UNK1:C | Alkyl | 4.51141 |
|  |  | N:UNK1:C | Alkyl | 4.09781 |
|  |  | N:UNK1:C | Alkyl | 4.7016 |
|  |  | N:UNK1 | Pi-Alkyl | 5.30596 |
|  |  | N:UNK1 | Pi-Alkyl | 4.28408 |
|  |  | N:UNK1 | Pi-Alkyl | 4.54527 |
|  |  | N:UNK1 | Pi-Alkyl | 5.31831 |
|  |  | N:UNK1 | Pi-Alkyl | 4.31704 |
| **10033008+**  **3U4K** | **-6.5** | A:ALA88:HN | Conventional Hydrogen Bond | 2.40233 |
|  |  | A:SER93:HG | Conventional Hydrogen Bond | 2.16367 |
|  |  | A:ILE99:HN | Conventional Hydrogen Bond | 2.63872 |
|  |  | A:THR138:HG1 | Conventional Hydrogen Bond | 2.86068 |
|  |  | A:SER139:HG | Conventional Hydrogen Bond | 2.32013 |
|  |  | N:UNK1:H | Conventional Hydrogen Bond | 1.80241 |
|  |  | N:UNK1:H | Conventional Hydrogen Bond | 2.61454 |
|  |  | N:UNK1:H | Conventional Hydrogen Bond | 2.08774 |
|  |  | A:ALA88 | Alkyl | 3.82915 |
|  |  | N:UNK1:C | Alkyl | 4.30551 |
|  |  | N:UNK1:C | Alkyl | 4.67299 |
| **10033008+**  **3U4K** | **-6.4** | N:UNK1:H | Conventional Hydrogen Bond | 1.92017 |
|  |  | N:UNK1 | Pi-Alkyl | 5.39231 |
|  |  | N:UNK1 | Pi-Alkyl | 3.90735 |
|  |  | N:UNK1 | Pi-Alkyl | 4.79324 |
|  |  | N:UNK1 | Pi-Alkyl | 3.84653 |
|  |  | N:UNK1 | Pi-Alkyl | 5.33442 |
| **9888256+**  **3U4K** | **-6.4** | A:VAL79:HN | Conventional Hydrogen Bond | 2.59341 |
|  |  | N:UNK1:H | Conventional Hydrogen Bond | 2.55247 |
|  |  | N:UNK1:H | Conventional Hydrogen Bond | 2.34794 |
|  |  | N:UNK1:H | Conventional Hydrogen Bond | 2.43224 |
|  |  | A:TYR92 | Pi-Pi T-shaped | 5.1966 |
|  |  | N:UNK1 | Pi-Alkyl | 4.10053 |
|  |  | N:UNK1 | Pi-Alkyl | 4.56441 |
|  |  | N:UNK1 | Pi-Alkyl | 4.95184 |
| **98050020+**  **3U4K** | **-6.4** | A:ARG125:HN | Conventional Hydrogen Bond | 2.60919 |
|  |  | A:ARG125:HE | Conventional Hydrogen Bond | 2.55236 |
|  |  | N:UNK1:H | Conventional Hydrogen Bond | 2.94706 |
|  |  | N:UNK1:H | Conventional Hydrogen Bond | 2.35806 |
|  |  | N:UNK1 | Alkyl | 5.20515 |
|  |  | A:TYR117 | Pi-Alkyl | 5.26428 |
|  |  | A:PHE124 | Pi-Alkyl | 4.6532 |
|  |  | A:PHE124 | Pi-Alkyl | 4.7781 |
| **638297+**  **3U4K** | **-6.4** | A:THR119:HG1 | Conventional Hydrogen Bond | 2.36672 |
|  |  | A:ARG125:HN | Conventional Hydrogen Bond | 2.4926 |
|  |  | A:ARG125:HE | Conventional Hydrogen Bond | 2.83088 |
|  |  | N:UNK1:H | Conventional Hydrogen Bond | 1.92802 |
|  |  | N:UNK1:H | Conventional Hydrogen Bond | 2.37308 |
|  |  | N:UNK1:H | Conventional Hydrogen Bond | 1.94718 |
|  |  | N:UNK1:C | Carbon Hydrogen Bond | 3.5685 |
| **54680453+**  **3U4K** | **-6.4** | A:ARG125:HN | Conventional Hydrogen Bond | 2.59394 |
|  |  | A:ARG125:HE | Conventional Hydrogen Bond | 2.57229 |
|  |  | N:UNK1 | Alkyl | 5.18585 |
|  |  | A:TYR117 | Pi-Alkyl | 5.28456 |
|  |  | A:PHE124 | Pi-Alkyl | 4.74271 |
|  |  | A:PHE124 | Pi-Alkyl | 4.82031 |
| **53483974+**  **3U4K** | **-6.4** | A:ARG125:HH22 | Conventional Hydrogen Bond | 2.85364 |
|  |  | N:UNK1:C | Carbon Hydrogen Bond | 3.63591 |
|  |  | N:UNK1:C | Pi-Sigma | 3.89379 |
|  |  | N:UNK1:C | Alkyl | 4.07419 |
|  |  | N:UNK1:C | Alkyl | 4.00475 |
|  |  | N:UNK1:C | Alkyl | 5.09628 |
|  |  | A:PHE124 | Pi-Alkyl | 5.28599 |
|  |  | A:PHE124 | Pi-Alkyl | 4.55346 |
|  |  | N:UNK1 | Pi-Alkyl | 4.78002 |
| **443440+**  **3U4K** | **-6.4** | A:TYR117:HH | Conventional Hydrogen Bond | 2.77827 |
|  |  | N:UNK1:H | Conventional Hydrogen Bond | 1.98897 |
|  |  | A:ALA108 | Alkyl | 4.53115 |
|  |  | A:ALA127 | Alkyl | 4.41747 |
| **441556+**  **3U4K** | **-6.4** | N:UNK1:H | Conventional Hydrogen Bond | 2.09792 |
|  |  | N:UNK1:H | Conventional Hydrogen Bond | 2.73752 |
|  |  | N:UNK1:C | Carbon Hydrogen Bond | 3.31602 |
|  |  | N:UNK1:C | Carbon Hydrogen Bond | 3.16216 |
|  |  | A:PHE124 | Pi-Pi Stacked | 4.22465 |
|  |  | N:UNK1 | Pi-Pi Stacked | 5.28947 |
|  |  | N:UNK1:C | Alkyl | 3.97024 |
|  |  | A:TYR117 | Pi-Alkyl | 5.25307 |
|  |  | N:UNK1 | Pi-Alkyl | 5.16113 |
|  |  | N:UNK1 | Pi-Alkyl | 5.1719 |
|  |  | N:UNK1 | Pi-Alkyl | 4.55284 |
| **38353601+**  **3U4K** | **-6.4** | A:SER122:HN | Conventional Hydrogen Bond | 2.23373 |
|  |  | A:SER123:HN | Conventional Hydrogen Bond | 2.35678 |
|  |  | A:GLY121:CA | Carbon Hydrogen Bond | 3.56522 |
|  |  | A:GLY121:CA | Carbon Hydrogen Bond | 3.41974 |
|  |  | N:UNK1:C | Carbon Hydrogen Bond | 3.43156 |
|  |  | A:ALA127 | Alkyl | 4.47932 |
| **21578704+**  **3U4K** | **-6.4** | A:TYR117:HH | Conventional Hydrogen Bond | 1.9946 |
|  |  | A:ARG125:HN | Conventional Hydrogen Bond | 2.34861 |
|  |  | N:UNK1:H | Conventional Hydrogen Bond | 2.60715 |
|  |  | A:PHE124:CA | Carbon Hydrogen Bond | 3.03166 |
|  |  | A:THR119:CG2 | Pi-Sigma | 3.96671 |
| **189063+**  **3U4K** | **-6.4** | N:UNK1:H | Conventional Hydrogen Bond | 2.63674 |
|  |  | A:SER110:OXT | Pi-Anion | 3.72947 |
|  |  | N:UNK1:C | Alkyl | 4.80817 |
|  |  | A:TYR117 | Pi-Alkyl | 4.67782 |
|  |  | A:TYR117 | Pi-Alkyl | 5.28532 |
|  |  | N:UNK1 | Pi-Alkyl | 4.2282 |
|  |  | N:UNK1 | Pi-Alkyl | 4.91159 |
| **139590671+**  **3U4K** | **-6.4** | A:ARG105:HH11 | Conventional Hydrogen Bond | 2.1252 |
|  |  | A:ARG105:HH21 | Conventional Hydrogen Bond | 2.58669 |
|  |  | A:THR119:HG1 | Conventional Hydrogen Bond | 1.99733 |
|  |  | A:ALA127 | Alkyl | 4.00004 |
|  |  | A:PHE124 | Pi-Alkyl | 4.45027 |
|  |  | A:PHE124 | Pi-Alkyl | 4.94282 |
| **139590667+**  **3U4K** | **-6.4** | A:THR119:HG1 | Conventional Hydrogen Bond | 2.77038 |
|  |  | A:ARG125:HH22 | Conventional Hydrogen Bond | 2.20432 |
|  |  | N:UNK1:H | Conventional Hydrogen Bond | 2.21839 |
|  |  | N:UNK1:H | Conventional Hydrogen Bond | 1.99012 |
|  |  | A:ALA108 | Alkyl | 4.33058 |
|  |  | A:ALA108 | Alkyl | 3.94677 |
|  |  | N:UNK1:C | Alkyl | 4.12565 |
|  |  | N:UNK1:C | Alkyl | 4.89063 |
|  |  | N:UNK1 | Alkyl | 5.22853 |
| **139590666+**  **3U4K** | **-6.4** | A:ARG125:HE | Conventional Hydrogen Bond | 1.98673 |
|  |  | A:ARG125:HH22 | Conventional Hydrogen Bond | 2.63117 |
|  |  | A:ARG125:HH22 | Conventional Hydrogen Bond | 2.83844 |
|  |  | N:UNK1:H | Conventional Hydrogen Bond | 2.55992 |
|  |  | N:UNK1:H | Conventional Hydrogen Bond | 2.47688 |
|  |  | A:PHE124 | Pi-Alkyl | 5.26456 |
| **139588442+**  **3U4K** | **-6.4** | A:THR119:HG1 | Pi-Donor Hydrogen Bond | 2.51755 |
|  |  | N:UNK1:C | Alkyl | 4.56667 |
|  |  | A:PHE124 | Pi-Alkyl | 5.14779 |
| **139583471+**  **3U4K** | **-6.4** | A:ALA88:HN | Conventional Hydrogen Bond | 2.30434 |
|  |  | A:THR94:HN | Conventional Hydrogen Bond | 2.73034 |
|  |  | N:UNK1:H | Conventional Hydrogen Bond | 2.97784 |
|  |  | N:UNK1:C | Carbon Hydrogen Bond | 3.59863 |
|  |  | A:ALA88 | Alkyl | 4.42621 |
|  |  | N:UNK1:C | Alkyl | 4.8036 |
| **132565337+**  **3U4K** | **-6.4** | A:SER75:HG | Conventional Hydrogen Bond | 2.08104 |
|  |  | A:ARG125:HN | Conventional Hydrogen Bond | 1.98379 |
|  |  | N:UNK1:C | Alkyl | 4.87299 |
|  |  | N:UNK1:C | Alkyl | 4.39859 |
|  |  | A:TYR117 | Pi-Alkyl | 5.44159 |
|  |  | A:TYR117 | Pi-Alkyl | 4.82875 |
| **132502750+**  **3U4K** | **-6.4** | A:SER75:HG | Conventional Hydrogen Bond | 2.08104 |
|  |  | A:ARG125:HN | Conventional Hydrogen Bond | 1.98379 |
|  |  | N:UNK1:C | Alkyl | 4.87299 |
|  |  | N:UNK1:C | Alkyl | 4.39859 |
|  |  | A:TYR117 | Pi-Alkyl | 5.44159 |
|  |  | A:TYR117 | Pi-Alkyl | 4.82875 |
| **131350+**  **3U4K** | **-6.4** | N:UNK1:H | Conventional Hydrogen Bond | 2.53684 |
|  |  | N:UNK1:H | Conventional Hydrogen Bond | 2.77506 |
|  |  | N:UNK1:H | Conventional Hydrogen Bond | 2.36028 |
|  |  | N:UNK1:H | Conventional Hydrogen Bond | 2.74969 |
|  |  | N:UNK1:H | Conventional Hydrogen Bond | 2.21384 |
|  |  | A:THR132:CG2 | Pi-Sigma | 3.98317 |
|  |  | A:THR132:CG2 | Pi-Sigma | 3.88912 |
|  |  | N:UNK1 | Pi-Alkyl | 5.40243 |
|  |  | N:UNK1 | Pi-Alkyl | 5.41934 |
| **11471114+**  **3U4K** | **-6.4** | A:ARG125:HN | Conventional Hydrogen Bond | 2.41623 |
|  |  | A:GLY121:CA | Carbon Hydrogen Bond | 3.71188 |
|  |  | A:PHE124 | Pi-Alkyl | 5.39552 |
| **11336960+**  **3U4K** | **-6.4** | A:ARG105:HH12 | Conventional Hydrogen Bond | 2.24902 |
|  |  | N:UNK1:H | Conventional Hydrogen Bond | 2.27309 |
|  |  | N:UNK1:H | Conventional Hydrogen Bond | 2.84788 |
|  |  | N:UNK1:H | Conventional Hydrogen Bond | 2.10351 |
|  |  | A:TYR117 | Pi-Pi T-shaped | 5.06649 |
|  |  | A:ALA77 | Alkyl | 3.47037 |
|  |  | A:ALA77 | Alkyl | 4.37269 |
|  |  | N:UNK1:C | Alkyl | 4.68736 |
|  |  | A:TYR113 | Pi-Alkyl | 4.20554 |
|  |  | A:TYR155 | Pi-Alkyl | 4.5399 |
|  |  | N:UNK1 | Pi-Alkyl | 5.3947 |
| **11152290+**  **3U4K** | **-6.4** | A:TYR117:HH | Conventional Hydrogen Bond | 1.98294 |
|  |  | A:ARG125:HH21 | Conventional Hydrogen Bond | 2.34336 |
|  |  | A:ALA108 | Alkyl | 4.18938 |
|  |  | N:UNK1 | Alkyl | 4.93977 |
|  |  | A:TYR117 | Pi-Alkyl | 5.32003 |
|  |  | A:PHE124 | Pi-Alkyl | 4.52353 |
| **10033009+**  **3U4K** | **-6.4** | A:GLY47:HN | Conventional Hydrogen Bond | 2.89599 |
|  |  | A:SER93:HG | Conventional Hydrogen Bond | 2.3122 |
|  |  | N:UNK1:H | Conventional Hydrogen Bond | 1.92598 |
|  |  | N:UNK1:C | Alkyl | 3.74647 |
|  |  | N:UNK1:C | Alkyl | 4.22977 |
|  |  | N:UNK1 | Pi-Alkyl | 5.47253 |
| **93306+**  **3U4K** | **-6.3** | A:TYR155:HH | Conventional Hydrogen Bond | 2.19597 |
|  |  | A:ALA127 | Alkyl | 4.7737 |
|  |  | A:TYR117 | Pi-Alkyl | 5.44854 |
|  |  | A:TYR117 | Pi-Alkyl | 4.61056 |
|  |  | A:PHE124 | Pi-Alkyl | 4.66756 |
| **7074739+**  **3U4K** | **-6.3** | A:ARG125:HN | Conventional Hydrogen Bond | 2.1982 |
|  |  | N:UNK1:H | Conventional Hydrogen Bond | 2.03207 |
|  |  | A:PHE124 | Pi-Alkyl | 5.13985 |
| **6475274+**  **3U4K** | **-6.3** | N:UNK1:C | Alkyl | 4.12876 |
|  |  | A:TYR29 | Pi-Alkyl | 4.41227 |
|  |  | A:TYR64 | Pi-Alkyl | 5.25103 |
| **6450508+**  **3U4K** | **-6.3** | A:THR119:HG1 | Conventional Hydrogen Bond | 2.05317 |
|  |  | A:ARG125:HE | Conventional Hydrogen Bond | 2.16594 |
|  |  | A:ARG125:HH21 | Conventional Hydrogen Bond | 2.59458 |
|  |  | A:ARG125:HH22 | Conventional Hydrogen Bond | 1.99475 |
|  |  | N:UNK1:H | Conventional Hydrogen Bond | 2.51303 |
|  |  | N:UNK1:H | Conventional Hydrogen Bond | 2.27583 |
|  |  | N:UNK1:H | Conventional Hydrogen Bond | 2.04764 |
|  |  | N:UNK1:H | Conventional Hydrogen Bond | 2.36492 |
|  |  | N:UNK1:C | Carbon Hydrogen Bond | 3.59131 |
|  |  | N:UNK1:C | Carbon Hydrogen Bond | 3.53103 |
|  |  | N:UNK1:C | Alkyl | 4.47138 |
|  |  | A:TYR117 | Pi-Alkyl | 5.23386 |
|  |  | A:PHE124 | Pi-Alkyl | 5.05966 |
|  |  | A:TYR155 | Pi-Alkyl | 5.3224 |
| **54754212+**  **3U4K** | **-6.3** | A:GLY83:HN | Conventional Hydrogen Bond | 2.42282 |
|  |  | N:UNK1:H | Conventional Hydrogen Bond | 2.17858 |
|  |  | N:UNK1:H | Conventional Hydrogen Bond | 2.43603 |
|  |  | N:UNK1:H | Conventional Hydrogen Bond | 2.04177 |
|  |  | N:UNK1:C | Pi-Sigma | 3.7648 |
|  |  | A:LYS78 | Alkyl | 4.30952 |
|  |  | A:VAL80 | Alkyl | 5.45226 |
|  |  | N:UNK1:C | Alkyl | 4.02329 |
|  |  | N:UNK1:C | Alkyl | 4.98804 |
| **5351516+**  **3U4K** | **-6.3** | A:LYS78 | Alkyl | 5.17102 |
|  |  | A:LYS78 | Alkyl | 5.34639 |
|  |  | A:LYS78 | Alkyl | 4.46169 |
|  |  | A:PRO114 | Alkyl | 5.24341 |
|  |  | A:TYR92 | Pi-Alkyl | 5.13857 |
|  |  | A:TYR92 | Pi-Alkyl | 5.11429 |
| **44231742+**  **3U4K** | **-6.3** | A:THR119:HG1 | Conventional Hydrogen Bond | 2.32677 |
|  |  | N:UNK1:H | Conventional Hydrogen Bond | 2.22059 |
|  |  | N:UNK1:H | Conventional Hydrogen Bond | 2.85869 |
|  |  | A:THR119:CG2 | Pi-Sigma | 3.80583 |
|  |  | A:TYR117 | Pi-Alkyl | 5.12193 |
| **44178844+**  **3U4K** | **-6.3** | A:ASN70:HD22 | Conventional Hydrogen Bond | 2.59526 |
|  |  | A:SER122:HN | Conventional Hydrogen Bond | 1.90208 |
|  |  | A:SER122:HG | Conventional Hydrogen Bond | 1.99706 |
|  |  | A:SER122:HG | Conventional Hydrogen Bond | 2.74075 |
|  |  | A:SER123:HN | Conventional Hydrogen Bond | 1.85123 |
|  |  | A:ARG125:HE | Conventional Hydrogen Bond | 2.89361 |
|  |  | A:ARG125:HH11 | Conventional Hydrogen Bond | 2.51145 |
|  |  | A:ARG125:HH22 | Conventional Hydrogen Bond | 2.74142 |
|  |  | N:UNK1:H | Conventional Hydrogen Bond | 2.21835 |
|  |  | N:UNK1:H | Conventional Hydrogen Bond | 2.41215 |
|  |  | N:UNK1:C | Carbon Hydrogen Bond | 3.47485 |
|  |  | A:PHE124 | Pi-Alkyl | 4.84539 |
| **16181015+**  **3U4K** | **-6.3** | A:THR119:HG1 | Conventional Hydrogen Bond | 2.32677 |
|  |  | N:UNK1:H | Conventional Hydrogen Bond | 2.22059 |
|  |  | N:UNK1:H | Conventional Hydrogen Bond | 2.85869 |
|  |  | A:THR119:CG2 | Pi-Sigma | 3.80583 |
|  |  | A:TYR117 | Pi-Alkyl | 5.12193 |
| **156582117+**  **3U4K** | **-6.3** | A:ARG125:HH21 | Conventional Hydrogen Bond | 2.88437 |
|  |  | A:ALA108 | Alkyl | 3.8026 |
|  |  | A:ALA127 | Alkyl | 4.44839 |
| **14421+**  **3U4K** | **-6.3** | A:ARG102:HH21 | Conventional Hydrogen Bond | 2.75484 |
|  |  | N:UNK1:C | Carbon Hydrogen Bond | 3.33781 |
|  |  | N:UNK1:C | Carbon Hydrogen Bond | 3.46077 |
|  |  | A:ASP134:OD2 | Pi-Anion | 4.01674 |
|  |  | A:LYS106 | Alkyl | 5.03118 |
|  |  | A:ILE112 | Alkyl | 4.80864 |
|  |  | N:UNK1 | Pi-Alkyl | 4.78275 |
| **139589964+**  **3U4K** | **-6.3** | A:THR138:HG1 | Conventional Hydrogen Bond | 2.68874 |
|  |  | A:THR138:CG2 | Pi-Sigma | 3.97802 |
|  |  | N:UNK1:C | Alkyl | 4.39119 |
|  |  | N:UNK1 | Pi-Alkyl | 4.19175 |
| **139587751+**  **3U4K** | **-6.3** | A:TYR117:HH | Conventional Hydrogen Bond | 2.11883 |
|  |  | A:THR119:HG1 | Conventional Hydrogen Bond | 2.32022 |
|  |  | N:UNK1:H | Conventional Hydrogen Bond | 2.14674 |
|  |  | A:ALA127 | Alkyl | 4.28189 |
|  |  | N:UNK1:C | Alkyl | 3.81073 |
|  |  | N:UNK1:C | Alkyl | 5.44786 |
|  |  | A:TYR155 | Pi-Alkyl | 4.82322 |
| **115252+**  **3U4K** | **-6.3** | A:THR120:HN | Conventional Hydrogen Bond | 2.15814 |
|  |  | A:TYR117:HH | Pi-Donor Hydrogen Bond | 2.97335 |
|  |  | A:THR119:HG1 | Pi-Donor Hydrogen Bond | 3.03658 |
|  |  | A:THR119:HG1 | Pi-Donor Hydrogen Bond | 2.41567 |
|  |  | A:ALA108 | Alkyl | 3.74603 |
|  |  | N:UNK1:C | Alkyl | 5.00693 |
|  |  | N:UNK1:C | Alkyl | 4.05368 |
|  |  | A:TYR117 | Pi-Alkyl | 5.06945 |
|  |  | A:TYR117 | Pi-Alkyl | 5.4963 |
|  |  | A:TYR155 | Pi-Alkyl | 5.38426 |
| **114855+**  **3U4K** | **-6.3** | N:UNK1:C | Carbon Hydrogen Bond | 3.40983 |
|  |  | A:ASP134:OD1 | Pi-Anion | 4.93593 |
|  |  | A:ASP134:OD2 | Pi-Anion | 4.6121 |
|  |  | A:ASP134:OD2 | Pi-Anion | 4.83608 |
|  |  | A:THR132:CG2 | Pi-Sigma | 3.77193 |
|  |  | A:VAL49 | Alkyl | 4.82272 |
|  |  | N:UNK1:C | Alkyl | 3.55669 |
| **10687292+**  **3U4K** | **-6.3** | A:TYR117:HH | Conventional Hydrogen Bond | 2.65901 |
|  |  | A:THR119:HG1 | Conventional Hydrogen Bond | 2.67402 |
|  |  | A:ARG125:HH21 | Conventional Hydrogen Bond | 2.8207 |
|  |  | N:UNK1:H | Conventional Hydrogen Bond | 2.43452 |
|  |  | A:ARG125:HN | Pi-Donor Hydrogen Bond | 3.16957 |
|  |  | N:UNK1:C | Pi-Sigma | 3.97431 |
|  |  | A:ALA127 | Alkyl | 4.1713 |
|  |  | N:UNK1:C | Alkyl | 3.94619 |
|  |  | N:UNK1:C | Alkyl | 5.03169 |
|  |  | N:UNK1:C | Alkyl | 3.94701 |
|  |  | A:PHE124 | Pi-Alkyl | 5.19373 |
|  |  | A:PHE124 | Pi-Alkyl | 4.10688 |
|  |  | N:UNK1 | Pi-Alkyl | 5.35116 |
| **105113+**  **3U4K** | **-6.3** | N:UNK1:H | Conventional Hydrogen Bond | 1.88743 |
|  |  | N:UNK1:C | Alkyl | 3.91998 |
|  |  | N:UNK1:C | Alkyl | 4.53471 |
| **101201+**  **3U4K** | **-6.3** | A:TYR117:HH | Conventional Hydrogen Bond | 2.81205 |
|  |  | A:THR119:HG1 | Conventional Hydrogen Bond | 1.76426 |
|  |  | A:ARG125:HH21 | Conventional Hydrogen Bond | 2.98163 |
|  |  | A:TYR155:HH | Conventional Hydrogen Bond | 2.23044 |
|  |  | N:UNK1 | Pi-Pi Stacked | 5.3305 |
|  |  | N:UNK1 | Pi-Alkyl | 5.44139 |
| **101031197+**  **3U4K** | **-6.3** | A:ARG125:HN | Conventional Hydrogen Bond | 2.42312 |
|  |  | A:ARG125:HE | Conventional Hydrogen Bond | 2.78336 |
|  |  | A:TYR117 | Pi-Alkyl | 4.87844 |
| **9863860+**  **3U4K** | **-6.2** | A:ARG125:HE | Conventional Hydrogen Bond | 2.82393 |
|  |  | A:ARG125:HH11 | Conventional Hydrogen Bond | 2.3187 |
|  |  | A:ARG125:HH22 | Conventional Hydrogen Bond | 2.57768 |
|  |  | N:UNK1:H | Conventional Hydrogen Bond | 2.47688 |
|  |  | A:PHE124:CA | Carbon Hydrogen Bond | 3.24099 |
|  |  | A:ALA127 | Alkyl | 5.37799 |
|  |  | N:UNK1:C | Alkyl | 4.61206 |
|  |  | A:TYR117 | Pi-Alkyl | 4.82244 |
| **54691340+**  **3U4K** | **-6.2** | A:GLY121:CA | Carbon Hydrogen Bond | 3.69326 |
|  |  | A:ARG125 | Alkyl | 4.90232 |
|  |  | N:UNK1:C | Alkyl | 5.30541 |
|  |  | N:UNK1:C | Alkyl | 4.56516 |
|  |  | N:UNK1:C | Alkyl | 4.7725 |
|  |  | N:UNK1:C | Alkyl | 4.77732 |
|  |  | N:UNK1:C | Alkyl | 4.62711 |
|  |  | A:TYR117 | Pi-Alkyl | 5.07697 |
|  |  | A:TYR117 | Pi-Alkyl | 5.48456 |
|  |  | A:PHE124 | Pi-Alkyl | 5.0983 |
|  |  | A:PHE124 | Pi-Alkyl | 4.50795 |
|  |  | A:PHE124 | Pi-Alkyl | 4.51443 |
|  |  | A:TYR155 | Pi-Alkyl | 5.4321 |
| **54685487+**  **3U4K** | **-6.2** | A:THR119:HG1 | Conventional Hydrogen Bond | 2.46225 |
|  |  | A:ARG125:HN | Conventional Hydrogen Bond | 2.73224 |
|  |  | N:UNK1:C | Carbon Hydrogen Bond | 3.50396 |
|  |  | N:UNK1:C | Alkyl | 4.64913 |
|  |  | N:UNK1 | Alkyl | 5.30143 |
|  |  | A:TYR117 | Pi-Alkyl | 5.05992 |
|  |  | A:PHE124 | Pi-Alkyl | 4.63062 |
| **477707+**  **3U4K** | **-6.2** | A:GLY36:HN2 | Conventional Hydrogen Bond | 2.23082 |
|  |  | N:UNK1:H | Conventional Hydrogen Bond | 1.80167 |
|  |  | A:ALA51:CB | Pi-Sigma | 3.74088 |
|  |  | N:UNK1 | Pi-Alkyl | 5.4608 |
|  |  | N:UNK1 | Pi-Alkyl | 4.23786 |
|  |  | N:UNK1 | Pi-Alkyl | 4.54211 |
|  |  | N:UNK1 | Pi-Alkyl | 3.91296 |
|  |  | N:UNK1 | Pi-Alkyl | 5.45609 |
| **44587537+**  **3U4K** | **-6.2** | A:SER75:HG | Conventional Hydrogen Bond | 2.22537 |
|  |  | A:ARG105:HH11 | Conventional Hydrogen Bond | 2.23314 |
|  |  | A:ARG105:HH21 | Conventional Hydrogen Bond | 2.33136 |
|  |  | A:TYR117:HH | Conventional Hydrogen Bond | 1.95266 |
|  |  | A:TYR155:HH | Pi-Donor Hydrogen Bond | 3.18694 |
|  |  | A:PHE124 | Pi-Alkyl | 4.8799 |
| **44127634+**  **3U4K** | **-6.2** | A:THR119:HG1 | Conventional Hydrogen Bond | 1.97205 |
|  |  | A:TYR155:HH | Conventional Hydrogen Bond | 2.58767 |
|  |  | N:UNK1:H | Conventional Hydrogen Bond | 3.02621 |
|  |  | A:GLY121:CA | Carbon Hydrogen Bond | 3.14853 |
|  |  | N:UNK1 | Alkyl | 5.28585 |
|  |  | N:UNK1:C | Alkyl | 5.34087 |
|  |  | N:UNK1:C | Alkyl | 4.78067 |
|  |  | A:TYR117 | Pi-Alkyl | 5.1062 |
|  |  | A:PHE124 | Pi-Alkyl | 4.44702 |
| **2724362+**  **3U4K** | **-6.2** | A:ARG102:HH21 | Conventional Hydrogen Bond | 2.87402 |
|  |  | A:ARG102:HH22 | Conventional Hydrogen Bond | 2.72083 |
|  |  | N:UNK1:C | Carbon Hydrogen Bond | 3.34255 |
|  |  | N:UNK1:C | Carbon Hydrogen Bond | 3.66667 |
|  |  | A:ASP134:OD2 | Pi-Anion | 3.93089 |
|  |  | A:ILE112 | Alkyl | 4.77991 |
|  |  | N:UNK1 | Pi-Alkyl | 4.87653 |
| **2724360+**  **3U4K** | **-6.2** | A:GLY36:HN3 | Conventional Hydrogen Bond | 2.61539 |
|  |  | A:GLY36:HN2 | Conventional Hydrogen Bond | 3.09106 |
|  |  | A:THR52:HN | Conventional Hydrogen Bond | 2.3427 |
|  |  | A:ALA51:CB | Pi-Sigma | 3.93976 |
|  |  | A:ALA51 | Alkyl | 4.3768 |
|  |  | A:LYS53 | Alkyl | 4.2977 |
|  |  | A:ILE135 | Alkyl | 5.17393 |
|  |  | A:ILE171 | Alkyl | 5.24682 |
|  |  | N:UNK1 | Pi-Alkyl | 4.76052 |
|  |  | N:UNK1 | Pi-Alkyl | 4.63075 |
|  |  | N:UNK1 | Pi-Alkyl | 5.49857 |
| **1794427+**  **3U4K** | **-6.2** | A:SER75:HG | Conventional Hydrogen Bond | 1.95321 |
|  |  | A:TYR117:HH | Conventional Hydrogen Bond | 2.25759 |
|  |  | A:ARG125:HN | Conventional Hydrogen Bond | 2.97034 |
|  |  | A:ARG125:HE | Conventional Hydrogen Bond | 2.7938 |
|  |  | A:ARG125:HH22 | Conventional Hydrogen Bond | 2.83107 |
|  |  | N:UNK1:H | Conventional Hydrogen Bond | 2.73164 |
|  |  | N:UNK1:H | Conventional Hydrogen Bond | 1.91581 |
|  |  | A:GLY121:CA | Carbon Hydrogen Bond | 3.55781 |
|  |  | A:PHE124:CA | Carbon Hydrogen Bond | 3.23411 |
|  |  | A:TYR155:HH | Pi-Donor Hydrogen Bond | 3.1733 |
|  |  | A:TYR117 | Pi-Pi T-shaped | 5.60212 |
| **16681748+**  **3U4K** | **-6.2** | A:THR119:HG1 | Conventional Hydrogen Bond | 2.40511 |
|  |  | N:UNK1:H | Conventional Hydrogen Bond | 2.22461 |
|  |  | N:UNK1:H | Conventional Hydrogen Bond | 3.03622 |
|  |  | A:THR119:HG1 | Pi-Donor Hydrogen Bond | 2.74397 |
|  |  | A:THR119:CG2 | Pi-Sigma | 3.79649 |
|  |  | N:UNK1:C | Alkyl | 5.05494 |
|  |  | A:PHE124 | Pi-Alkyl | 5.24048 |
|  |  | A:TYR155 | Pi-Alkyl | 4.80884 |
| **14841097+**  **3U4K** | **-6.2** | A:ARG105:HH11 | Conventional Hydrogen Bond | 2.02025 |
|  |  | A:ARG105:HH21 | Conventional Hydrogen Bond | 2.83039 |
|  |  | A:TYR117 | Pi-Pi T-shaped | 5.4654 |
|  |  | N:UNK1:C | Alkyl | 3.89393 |
|  |  | N:UNK1:C | Alkyl | 4.4469 |
|  |  | N:UNK1:C | Alkyl | 3.59218 |
|  |  | A:PHE124 | Pi-Alkyl | 5.12648 |
|  |  | A:PHE124 | Pi-Alkyl | 4.0424 |
|  |  | N:UNK1 | Pi-Alkyl | 5.06553 |
| **147992277+**  **3U4K** | **-6.2** | A:ARG125:HN | Conventional Hydrogen Bond | 2.21571 |
|  |  | A:TYR155:HH | Conventional Hydrogen Bond | 2.12021 |
|  |  | A:ALA127 | Alkyl | 3.85352 |
|  |  | N:UNK1:C | Alkyl | 5.05243 |
|  |  | A:PHE124 | Pi-Alkyl | 5.30183 |
|  |  | A:PHE124 | Pi-Alkyl | 4.78208 |
|  |  | A:TYR155 | Pi-Alkyl | 4.89604 |
| **14309394+**  **3U4K** | **-6.2** | A:ALA127:HN | Conventional Hydrogen Bond | 2.86632 |
|  |  | A:ALA127:HN | Conventional Hydrogen Bond | 1.9487 |
|  |  | A:ALA108 | Alkyl | 3.77392 |
|  |  | A:ALA127 | Alkyl | 3.79491 |
|  |  | N:UNK1 | Pi-Alkyl | 5.36091 |
| **139590670+**  **3U4K** | **-6.2** | A:ARG125:HN | Conventional Hydrogen Bond | 2.28513 |
|  |  | N:UNK1:H | Conventional Hydrogen Bond | 2.71508 |
|  |  | N:UNK1:C | Alkyl | 3.85576 |
|  |  | A:PHE124 | Pi-Alkyl | 5.43996 |
| **139590666+**  **3U4K** | **-6.2** | A:ARG125:HE | Conventional Hydrogen Bond | 1.98673 |
|  |  | A:ARG125:HH22 | Conventional Hydrogen Bond | 2.63117 |
|  |  | A:ARG125:HH22 | Conventional Hydrogen Bond | 2.83844 |
|  |  | N:UNK1:H | Conventional Hydrogen Bond | 2.55992 |
|  |  | N:UNK1:H | Conventional Hydrogen Bond | 2.47688 |
|  |  | A:PHE124 | Pi-Alkyl | 5.26456 |
| **139589965+**  **3U4K** | **-6.2** | N:UNK1:H | Conventional Hydrogen Bond | 2.86794 |
|  |  | A:ALA88:HN | Pi-Donor Hydrogen Bond | 2.77649 |
|  |  | A:ALA88 | Alkyl | 3.85212 |
|  |  | N:UNK1:C | Alkyl | 4.4136 |
|  |  | N:UNK1 | Pi-Alkyl | 4.15329 |
| **139589964+**  **3U4K** | **-6.2** | A:THR138:HG1 | Conventional Hydrogen Bond | 2.68874 |
|  |  | A:THR138:CG2 | Pi-Sigma | 3.97802 |
|  |  | N:UNK1:C | Alkyl | 4.39119 |
|  |  | N:UNK1 | Pi-Alkyl | 4.19175 |
| **139585195+**  **3U4K** | **-6.2** | N:UNK1:H | Conventional Hydrogen Bond | 2.2498 |
|  |  | A:ALA51:CB | Pi-Sigma | 3.79878 |
|  |  | N:UNK1:C | Alkyl | 4.03187 |
|  |  | N:UNK1:C | Alkyl | 4.5373 |
|  |  | N:UNK1:C | Alkyl | 4.20956 |
|  |  | N:UNK1:C | Alkyl | 4.64492 |
|  |  | N:UNK1:C | Alkyl | 5.46154 |
|  |  | N:UNK1 | Pi-Alkyl | 5.07737 |
|  |  | N:UNK1 | Pi-Alkyl | 5.37001 |
|  |  | N:UNK1 | Pi-Alkyl | 4.43599 |
|  |  | N:UNK1 | Pi-Alkyl | 4.49534 |
|  |  | N:UNK1 | Pi-Alkyl | 5.27437 |
| **139086489+**  **3U4K** | **-6.2** | A:TYR117:HH | Conventional Hydrogen Bond | 2.20088 |
|  |  | A:ALA108 | Alkyl | 4.2536 |
|  |  | A:ARG125 | Alkyl | 5.41543 |
|  |  | A:ALA127 | Alkyl | 4.18684 |
|  |  | A:ALA127 | Alkyl | 3.86777 |
|  |  | N:UNK1:C | Alkyl | 5.19462 |
|  |  | N:UNK1:C | Alkyl | 3.84758 |
|  |  | A:PHE124 | Pi-Alkyl | 4.84673 |
| **135426831+**  **3U4K** | **-6.2** | A:CYS178:HN | Conventional Hydrogen Bond | 1.86181 |
|  |  | N:UNK1:H | Conventional Hydrogen Bond | 2.44715 |
|  |  | N:UNK1:H | Conventional Hydrogen Bond | 2.12458 |
|  |  | A:SER175:CB | Carbon Hydrogen Bond | 3.54864 |
|  |  | A:THR142:HN | Pi-Donor Hydrogen Bond | 3.08504 |
|  |  | N:UNK1:C | Alkyl | 4.27003 |
| **132565337+**  **3U4K** | **-6.2** | A:SER75:HG | Conventional Hydrogen Bond | 2.08104 |
|  |  | A:ARG125:HN | Conventional Hydrogen Bond | 1.98379 |
|  |  | N:UNK1:C | Alkyl | 4.87299 |
|  |  | N:UNK1:C | Alkyl | 4.39859 |
|  |  | A:TYR117 | Pi-Alkyl | 5.44159 |
|  |  | A:TYR117 | Pi-Alkyl | 4.82875 |
| **127041692+**  **3U4K** | **-6.2** | A:TYR117:HH | Conventional Hydrogen Bond | 2.63209 |
|  |  | A:THR119:HG1 | Conventional Hydrogen Bond | 2.11033 |
|  |  | A:ARG125:HH22 | Conventional Hydrogen Bond | 2.58358 |
|  |  | A:ARG125:NH2 | Pi-Cation | 4.67796 |
|  |  | A:ARG125:HE | Pi-Donor Hydrogen Bond | 2.96523 |
|  |  | N:UNK1:C | Pi-Sigma | 3.72756 |
|  |  | N:UNK1:C | Alkyl | 4.30443 |
|  |  | N:UNK1:C | Alkyl | 4.12314 |
|  |  | N:UNK1:C | Alkyl | 3.91605 |
|  |  | A:PHE124 | Pi-Alkyl | 5.30372 |
|  |  | N:UNK1 | Pi-Alkyl | 4.82009 |
|  |  | N:UNK1 | Pi-Alkyl | 4.70598 |
| **11767849+**  **3U4K** | **-6.2** | A:TYR117:HH | Conventional Hydrogen Bond | 2.31303 |
|  |  | N:UNK1:H | Conventional Hydrogen Bond | 2.85938 |
|  |  | N:UNK1:C | Pi-Sigma | 3.94183 |
|  |  | A:ALA77 | Alkyl | 5.05366 |
|  |  | A:ALA77 | Alkyl | 4.26627 |
|  |  | A:ALA108 | Alkyl | 3.79404 |
|  |  | A:ALA127 | Alkyl | 4.80457 |
|  |  | A:ALA127 | Alkyl | 4.66266 |
|  |  | A:ALA127 | Alkyl | 3.73542 |
|  |  | N:UNK1:C | Alkyl | 4.25144 |
|  |  | N:UNK1:C | Alkyl | 4.63395 |
|  |  | N:UNK1 | Alkyl | 4.99832 |
|  |  | A:TYR113 | Pi-Alkyl | 4.62187 |
|  |  | A:TYR117 | Pi-Alkyl | 5.42706 |
|  |  | A:TYR117 | Pi-Alkyl | 5.22781 |
|  |  | A:TYR117 | Pi-Alkyl | 5.10261 |
|  |  | A:PHE124 | Pi-Alkyl | 4.55433 |
| **10639+**  **3U4K** | **-6.2** | N:UNK1:H | Conventional Hydrogen Bond | 2.22064 |
|  |  | N:UNK1:H | Conventional Hydrogen Bond | 2.64075 |
|  |  | A:ASP134:OD1 | Pi-Anion | 3.85665 |
|  |  | A:THR132:CG2 | Pi-Sigma | 3.66491 |
|  |  | N:UNK1:C | Alkyl | 3.75007 |
|  |  | N:UNK1 | Pi-Alkyl | 5.42857 |
|  |  | N:UNK1 | Pi-Alkyl | 5.08494 |
| **10325700+**  **3U4K** | **-6.2** | A:THR160:HG1 | Conventional Hydrogen Bond | 2.6496 |
|  |  | N:UNK1:H | Conventional Hydrogen Bond | 1.93729 |
|  |  | A:THR160:CB | Carbon Hydrogen Bond | 3.45705 |
|  |  | N:UNK1:C | Carbon Hydrogen Bond | 3.40452 |
|  |  | N:UNK1:C | Carbon Hydrogen Bond | 3.52876 |
|  |  | A:GLU154:OE1 | Pi-Anion | 3.73292 |
|  |  | A:GLU154:OE1 | Pi-Anion | 3.46959 |
|  |  | A:ALA28 | Alkyl | 3.72016 |
|  |  | N:UNK1:C | Alkyl | 4.21234 |
|  |  | N:UNK1:C | Alkyl | 4.9708 |
|  |  | N:UNK1 | Pi-Alkyl | 4.91741 |
| **9863860+**  **3U4K** | **-6.1** | A:ARG125:HE | Conventional Hydrogen Bond | 2.82393 |
|  |  | A:ARG125:HH11 | Conventional Hydrogen Bond | 2.3187 |
|  |  | A:ARG125:HH22 | Conventional Hydrogen Bond | 2.57768 |
|  |  | N:UNK1:H | Conventional Hydrogen Bond | 2.47688 |
|  |  | A:PHE124:CA | Carbon Hydrogen Bond | 3.24099 |
|  |  | A:ALA127 | Alkyl | 5.37799 |
|  |  | N:UNK1:C | Alkyl | 4.61206 |
|  |  | A:TYR117 | Pi-Alkyl | 4.82244 |
| **78210124+**  **3U4K** | **-6.1** | A:ARG125:HN | Conventional Hydrogen Bond | 1.88203 |
|  |  | A:ARG125:HE | Conventional Hydrogen Bond | 2.21025 |
|  |  | A:ARG125:HH22 | Conventional Hydrogen Bond | 2.29712 |
|  |  | N:UNK1:H | Conventional Hydrogen Bond | 2.87207 |
|  |  | N:UNK1:C | Pi-Sigma | 3.88526 |
|  |  | N:UNK1:C | Alkyl | 4.12688 |
|  |  | N:UNK1:C | Alkyl | 4.12057 |
|  |  | N:UNK1:C | Alkyl | 5.11784 |
|  |  | A:PHE124 | Pi-Alkyl | 4.52695 |
| **6475274+**  **3U4K** | **-6.1** | N:UNK1:C | Alkyl | 4.12876 |
|  |  | A:TYR29 | Pi-Alkyl | 4.41227 |
|  |  | A:TYR64 | Pi-Alkyl | 5.25103 |
| **6450521+**  **3U4K** | **-6.1** | A:SER75:HN | Conventional Hydrogen Bond | 2.77444 |
|  |  | A:SER75:HG | Conventional Hydrogen Bond | 2.6932 |
|  |  | A:THR119:HG1 | Conventional Hydrogen Bond | 2.12925 |
|  |  | A:ARG125:HE | Conventional Hydrogen Bond | 3.00397 |
|  |  | A:ARG125:HH22 | Conventional Hydrogen Bond | 2.43229 |
|  |  | N:UNK1:H | Conventional Hydrogen Bond | 2.56591 |
|  |  | N:UNK1:H | Conventional Hydrogen Bond | 2.32079 |
|  |  | N:UNK1:H | Conventional Hydrogen Bond | 3.05836 |
|  |  | N:UNK1:H | Conventional Hydrogen Bond | 2.7624 |
|  |  | N:UNK1:H | Conventional Hydrogen Bond | 2.82771 |
|  |  | N:UNK1:H | Conventional Hydrogen Bond | 2.65848 |
|  |  | N:UNK1:H | Conventional Hydrogen Bond | 2.88969 |
|  |  | N:UNK1:C | Carbon Hydrogen Bond | 3.38766 |
|  |  | N:UNK1:C | Alkyl | 4.03463 |
|  |  | A:TYR117 | Pi-Alkyl | 5.28212 |
|  |  | A:PHE124 | Pi-Alkyl | 5.15739 |
|  |  | A:TYR155 | Pi-Alkyl | 5.36677 |
| **54707854+**  **3U4K** | **-6.1** | N:UNK1:H | Conventional Hydrogen Bond | 1.81335 |
|  |  | N:UNK1:H | Conventional Hydrogen Bond | 2.02215 |
|  |  | A:ILE109:CD | Pi-Sigma | 3.66817 |
|  |  | A:ALA108 | Alkyl | 3.87003 |
|  |  | A:ALA108 | Alkyl | 4.05916 |
|  |  | A:ALA127 | Alkyl | 4.01336 |
| **54685489+**  **3U4K** | **-6.1** | A:THR119:HG1 | Conventional Hydrogen Bond | 1.98861 |
|  |  | N:UNK1:C | Pi-Sigma | 3.9457 |
|  |  | N:UNK1 | Alkyl | 5.21469 |
|  |  | N:UNK1:C | Alkyl | 5.17168 |
|  |  | A:TYR117 | Pi-Alkyl | 4.97296 |
|  |  | A:PHE124 | Pi-Alkyl | 4.26142 |
| **54685487+**  **3U4K** | **-6.1** | A:THR119:HG1 | Conventional Hydrogen Bond | 2.46225 |
|  |  | A:ARG125:HN | Conventional Hydrogen Bond | 2.73224 |
|  |  | N:UNK1:C | Carbon Hydrogen Bond | 3.50396 |
|  |  | N:UNK1:C | Alkyl | 4.64913 |
|  |  | N:UNK1 | Alkyl | 5.30143 |
|  |  | A:TYR117 | Pi-Alkyl | 5.05992 |
|  |  | A:PHE124 | Pi-Alkyl | 4.63062 |
| **53483973+**  **3U4K** | **-6.1** | A:THR119:HG1 | Conventional Hydrogen Bond | 2.02341 |
|  |  | N:UNK1:H | Conventional Hydrogen Bond | 2.65021 |
|  |  | N:UNK1:C | Pi-Sigma | 3.87861 |
|  |  | N:UNK1 | Alkyl | 4.79237 |
|  |  | A:TYR117 | Pi-Alkyl | 5.23839 |
|  |  | A:PHE124 | Pi-Alkyl | 4.39052 |
| **44127634+**  **3U4K** | **-6.1** | A:THR119:HG1 | Conventional Hydrogen Bond | 1.97205 |
|  |  | A:TYR155:HH | Conventional Hydrogen Bond | 2.58767 |
|  |  | N:UNK1:H | Conventional Hydrogen Bond | 3.02621 |
|  |  | A:GLY121:CA | Carbon Hydrogen Bond | 3.14853 |
|  |  | N:UNK1 | Alkyl | 5.28585 |
|  |  | N:UNK1:C | Alkyl | 5.34087 |
|  |  | N:UNK1:C | Alkyl | 4.78067 |
|  |  | A:TYR117 | Pi-Alkyl | 5.1062 |
|  |  | A:PHE124 | Pi-Alkyl | 4.44702 |
| **42609855+**  **3U4K** | **-6.1** | A:LYS74:HZ1 | Conventional Hydrogen Bond | 2.09255 |
|  |  | A:LYS90:HZ1 | Conventional Hydrogen Bond | 2.57019 |
|  |  | A:SER116:HG | Conventional Hydrogen Bond | 2.60393 |
|  |  | N:UNK1:H | Conventional Hydrogen Bond | 2.40153 |
|  |  | N:UNK1:H | Conventional Hydrogen Bond | 2.16016 |
|  |  | N:UNK1:H | Conventional Hydrogen Bond | 2.21721 |
|  |  | N:UNK1:C | Carbon Hydrogen Bond | 3.22429 |
|  |  | A:GLN86:HE21 | Pi-Donor Hydrogen Bond | 3.15894 |
|  |  | N:UNK1:C | Alkyl | 4.66699 |
|  |  | A:TYR92 | Pi-Alkyl | 5.45517 |
|  |  | N:UNK1 | Pi-Alkyl | 5.10267 |
|  |  | N:UNK1 | Pi-Alkyl | 3.94039 |
|  |  | N:UNK1 | Pi-Alkyl | 4.41188 |
| **33960+**  **3U4K** | **-6.1** | A:PHE124 | Pi-Pi Stacked | 4.59049 |
|  |  | N:UNK1:C | Alkyl | 5.1587 |
|  |  | A:TYR117 | Pi-Alkyl | 5.26215 |
|  |  | N:UNK1 | Pi-Alkyl | 5.28836 |
|  |  | N:UNK1 | Pi-Alkyl | 4.54665 |
| **3220+**  **3U4K** | **-6.1** | A:ARG102:HH22 | Conventional Hydrogen Bond | 2.88736 |
|  |  | A:ASP134:OD1 | Pi-Anion | 4.22655 |
|  |  | A:THR132:CG2 | Pi-Sigma | 3.5616 |
|  |  | N:UNK1 | Pi-Alkyl | 4.73706 |
| **21119330+**  **3U4K** | **-6.1** | A:SER93:HN | Conventional Hydrogen Bond | 2.98926 |
|  |  | A:SER93:HG | Conventional Hydrogen Bond | 1.96146 |
|  |  | A:THR138:CG2 | Pi-Sigma | 3.32495 |
|  |  | A:VAL85 | Alkyl | 5.315 |
|  |  | N:UNK1 | Pi-Alkyl | 5.21957 |
|  |  | N:UNK1 | Pi-Alkyl | 5.24937 |
| **15558498+**  **3U4K** | **-6.1** | A:TYR117:HH | Conventional Hydrogen Bond | 2.81148 |
|  |  | A:THR119:HG1 | Conventional Hydrogen Bond | 2.84812 |
|  |  | A:ARG125:HH11 | Conventional Hydrogen Bond | 2.54687 |
|  |  | A:ARG125:HH21 | Conventional Hydrogen Bond | 2.3175 |
|  |  | N:UNK1:H | Conventional Hydrogen Bond | 1.91796 |
|  |  | A:ILE109:CD | Pi-Sigma | 3.72878 |
|  |  | A:ALA127:CB | Pi-Sigma | 3.96166 |
|  |  | N:UNK1 | Pi-Alkyl | 5.18716 |
|  |  | N:UNK1 | Pi-Alkyl | 5.013 |
|  |  | N:UNK1 | Pi-Alkyl | 4.29024 |
| **146682746+**  **3U4K** | **-6.1** | N:UNK1:H | Conventional Hydrogen Bond | 1.77163 |
|  |  | A:GLY87:CA | Pi-Sigma | 3.91465 |
|  |  | A:THR138:CG2 | Pi-Sigma | 3.81083 |
|  |  | N:UNK1 | Pi-Alkyl | 5.47242 |
| **139590672+**  **3U4K** | **-6.1** | A:PRO58 | Alkyl | 5.4067 |
|  |  | N:UNK1 | Alkyl | 5.16287 |
|  |  | N:UNK1:C | Alkyl | 4.54896 |
|  |  | N:UNK1:C | Alkyl | 4.43846 |
|  |  | A:TYR29 | Pi-Alkyl | 3.64097 |
|  |  | A:PHE131 | Pi-Alkyl | 5.26817 |
|  |  | N:UNK1 | Pi-Alkyl | 5.40512 |
|  |  | N:UNK1 | Pi-Alkyl | 5.14877 |
| **139590670+**  **3U4K** | **-6.1** | A:ARG125:HN | Conventional Hydrogen Bond | 2.28513 |
|  |  | N:UNK1:H | Conventional Hydrogen Bond | 2.71508 |
|  |  | N:UNK1:C | Alkyl | 3.85576 |
|  |  | A:PHE124 | Pi-Alkyl | 5.43996 |
| **139583471+**  **3U4K** | **-6.1** | A:ALA88:HN | Conventional Hydrogen Bond | 2.30434 |
|  |  | A:THR94:HN | Conventional Hydrogen Bond | 2.73034 |
|  |  | N:UNK1:H | Conventional Hydrogen Bond | 2.97784 |
|  |  | N:UNK1:C | Carbon Hydrogen Bond | 3.59863 |
|  |  | A:ALA88 | Alkyl | 4.42621 |
|  |  | N:UNK1:C | Alkyl | 4.8036 |
| **132556715+**  **3U4K** | **-6.1** | A:SER75:HG | Conventional Hydrogen Bond | 2.58694 |
|  |  | A:ILE109 | Alkyl | 5.48716 |
|  |  | A:ALA127 | Alkyl | 4.79737 |
|  |  | A:TYR117 | Pi-Alkyl | 5.33755 |
|  |  | A:PHE124 | Pi-Alkyl | 4.58237 |
| **127041692+**  **3U4K** | **-6.1** | A:TYR117:HH | Conventional Hydrogen Bond | 2.63209 |
|  |  | A:THR119:HG1 | Conventional Hydrogen Bond | 2.11033 |
|  |  | A:ARG125:HH22 | Conventional Hydrogen Bond | 2.58358 |
|  |  | A:ARG125:NH2 | Pi-Cation | 4.67796 |
|  |  | A:ARG125:HE | Pi-Donor Hydrogen Bond | 2.96523 |
|  |  | N:UNK1:C | Pi-Sigma | 3.72756 |
|  |  | N:UNK1:C | Alkyl | 4.30443 |
|  |  | N:UNK1:C | Alkyl | 4.12314 |
|  |  | N:UNK1:C | Alkyl | 3.91605 |
|  |  | A:PHE124 | Pi-Alkyl | 5.30372 |
|  |  | N:UNK1 | Pi-Alkyl | 4.82009 |
|  |  | N:UNK1 | Pi-Alkyl | 4.70598 |
| **11779662+**  **3U4K** | **-6.1** | A:ARG102:HH22 | Conventional Hydrogen Bond | 2.91957 |
|  |  | N:UNK1:H | Conventional Hydrogen Bond | 2.54982 |
|  |  | N:UNK1:H | Conventional Hydrogen Bond | 2.50795 |
|  |  | N:UNK1:H | Conventional Hydrogen Bond | 2.10819 |
|  |  | N:UNK1:H | Conventional Hydrogen Bond | 2.21909 |
|  |  | A:ASP134:OD1 | Pi-Anion | 4.07156 |
|  |  | A:THR132:CG2 | Pi-Sigma | 3.7624 |
|  |  | N:UNK1 | Pi-Alkyl | 4.78541 |
| **10639+**  **3U4K** | **-6.1** | N:UNK1:H | Conventional Hydrogen Bond | 2.22064 |
|  |  | N:UNK1:H | Conventional Hydrogen Bond | 2.64075 |
|  |  | A:ASP134:OD1 | Pi-Anion | 3.85665 |
|  |  | A:THR132:CG2 | Pi-Sigma | 3.66491 |
|  |  | N:UNK1:C | Alkyl | 3.75007 |
|  |  | N:UNK1 | Pi-Alkyl | 5.42857 |
|  |  | N:UNK1 | Pi-Alkyl | 5.08494 |
| **10369606+**  **3U4K** | **-6.1** | N:UNK1:H | Conventional Hydrogen Bond | 2.17401 |
|  |  | N:UNK1:H | Conventional Hydrogen Bond | 2.58217 |
|  |  | A:VAL80:CA | Carbon Hydrogen Bond | 3.78712 |
|  |  | N:UNK1:C | Carbon Hydrogen Bond | 3.61955 |
|  |  | A:GLY83:HN | Pi-Donor Hydrogen Bond | 2.64209 |
|  |  | A:GLY83:C,O;LEU84:N | Amide-Pi Stacked | 4.62907 |
|  |  | A:ALA82 | Alkyl | 3.73296 |
|  |  | A:PRO114 | Alkyl | 5.43289 |
|  |  | N:UNK1:C | Alkyl | 3.81386 |
|  |  | N:UNK1:C | Alkyl | 4.80355 |
|  |  | A:TYR92 | Pi-Alkyl | 4.90838 |
| **10286+**  **3U4K** | **-6.1** | A:TYR155:HH | Pi-Donor Hydrogen Bond | 3.07926 |
|  |  | A:PHE124 | Pi-Pi Stacked | 4.39401 |
|  |  | A:PHE124 | Pi-Pi Stacked | 5.9222 |
|  |  | A:TYR155 | Pi-Pi T-shaped | 5.71815 |
|  |  | N:UNK1 | Pi-Alkyl | 5.13654 |
|  |  | N:UNK1 | Pi-Alkyl | 5.48255 |
|  |  | N:UNK1 | Pi-Alkyl | 4.60241 |
| **101899579+**  **3U4K** | **-6.1** | N:UNK1:H | Conventional Hydrogen Bond | 2.46011 |
|  |  | N:UNK1:H | Conventional Hydrogen Bond | 2.11713 |
|  |  | N:UNK1:H | Conventional Hydrogen Bond | 2.22709 |
|  |  | A:THR132:CG2 | Pi-Sigma | 3.6701 |
|  |  | A:THR132:CG2 | Pi-Sigma | 3.62493 |
|  |  | A:THR132:CG2 | Pi-Sigma | 3.92934 |
|  |  | N:UNK1 | Pi-Alkyl | 4.96207 |
| **71473525+**  **3U4K** | **-6** | A:SER75:HG | Conventional Hydrogen Bond | 2.40573 |
|  |  | A:TYR155:HH | Conventional Hydrogen Bond | 1.83728 |
|  |  | A:TYR155:HH | Conventional Hydrogen Bond | 2.37111 |
|  |  | N:UNK1:H | Conventional Hydrogen Bond | 2.76293 |
|  |  | N:UNK1:H | Conventional Hydrogen Bond | 2.82509 |
|  |  | A:THR119:HG1 | Pi-Donor Hydrogen Bond | 2.99902 |
|  |  | N:UNK1:C | Alkyl | 5.28421 |
| **68108+**  **3U4K** | **-6** | A:TYR29 | Pi-Pi Stacked | 5.00042 |
|  |  | A:TYR29 | Pi-Pi Stacked | 4.10454 |
|  |  | N:UNK1 | Pi-Alkyl | 5.00249 |
|  |  | N:UNK1 | Pi-Alkyl | 4.88292 |
| **5275906+**  **3U4K** | **-6** | A:THR138:CG2 | Pi-Sigma | 3.47697 |
|  |  | N:UNK1:C | Alkyl | 3.83139 |
|  |  | N:UNK1 | Pi-Alkyl | 5.24434 |
| **44178749+**  **3U4K** | **-6** | A:ILE99:HN | Conventional Hydrogen Bond | 2.87759 |
|  |  | N:UNK1:H | Conventional Hydrogen Bond | 2.28064 |
|  |  | N:UNK1:H | Conventional Hydrogen Bond | 2.37605 |
|  |  | N:UNK1:H | Conventional Hydrogen Bond | 2.35908 |
|  |  | N:UNK1:H | Conventional Hydrogen Bond | 2.34509 |
|  |  | A:SER93:CB | Carbon Hydrogen Bond | 3.3097 |
|  |  | N:UNK1:C | Alkyl | 4.4216 |
|  |  | N:UNK1:C | Alkyl | 4.39425 |
| **24859514+**  **3U4K** | **-6** | A:TYR117:HH | Conventional Hydrogen Bond | 2.29273 |
|  |  | N:UNK1:H | Conventional Hydrogen Bond | 2.12691 |
|  |  | N:UNK1:H | Conventional Hydrogen Bond | 2.88311 |
|  |  | A:SER123:CB | Pi-Sigma | 3.99863 |
|  |  | N:UNK1:C | Pi-Sigma | 3.78483 |
|  |  | A:PHE124 | Pi-Alkyl | 5.00879 |
| **21596370+**  **3U4K** | **-6** | A:ARG125:HN | Conventional Hydrogen Bond | 2.37914 |
|  |  | N:UNK1:H | Conventional Hydrogen Bond | 2.65309 |
|  |  | N:UNK1:H | Conventional Hydrogen Bond | 1.98209 |
|  |  | N:UNK1:C | Alkyl | 4.41312 |
|  |  | N:UNK1 | Alkyl | 5.0249 |
|  |  | A:TYR117 | Pi-Alkyl | 5.42894 |
|  |  | A:PHE124 | Pi-Alkyl | 5.44305 |
|  |  | A:PHE124 | Pi-Alkyl | 4.56902 |
| **16132327+**  **3U4K** | **-6** | A:SER75:HG | Conventional Hydrogen Bond | 2.40573 |
|  |  | A:TYR155:HH | Conventional Hydrogen Bond | 1.83728 |
|  |  | A:TYR155:HH | Conventional Hydrogen Bond | 2.37111 |
|  |  | N:UNK1:H | Conventional Hydrogen Bond | 2.76293 |
|  |  | N:UNK1:H | Conventional Hydrogen Bond | 2.82509 |
|  |  | A:THR119:HG1 | Pi-Donor Hydrogen Bond | 2.99902 |
|  |  | N:UNK1:C | Alkyl | 5.28421 |
| **11336960+**  **3U4K** | **-6** | A:ARG105:HH12 | Conventional Hydrogen Bond | 2.24902 |
|  |  | N:UNK1:H | Conventional Hydrogen Bond | 2.27309 |
|  |  | N:UNK1:H | Conventional Hydrogen Bond | 2.84788 |
|  |  | N:UNK1:H | Conventional Hydrogen Bond | 2.10351 |
|  |  | A:TYR117 | Pi-Pi T-shaped | 5.06649 |
|  |  | A:ALA77 | Alkyl | 3.47037 |
|  |  | A:ALA77 | Alkyl | 4.37269 |
|  |  | N:UNK1:C | Alkyl | 4.68736 |
|  |  | A:TYR113 | Pi-Alkyl | 4.20554 |
|  |  | A:TYR155 | Pi-Alkyl | 4.5399 |
|  |  | N:UNK1 | Pi-Alkyl | 5.3947 |
| **11161848+**  **3U4K** | **-6** | A:ARG125:HN | Conventional Hydrogen Bond | 2.47559 |
|  |  | A:GLY121:CA | Carbon Hydrogen Bond | 3.72642 |
| **10393+**  **3U4K** | **-6** | A:SER75:HG | Pi-Donor Hydrogen Bond | 3.00063 |
|  |  | A:PHE124 | Pi-Pi Stacked | 5.23202 |
| **72945800+**  **3U4K** | **-5.9** | A:ARG125:HN | Conventional Hydrogen Bond | 2.26122 |
|  |  | A:ARG125:HH11 | Conventional Hydrogen Bond | 3.01899 |
|  |  | A:ARG125:HH21 | Conventional Hydrogen Bond | 2.95597 |
|  |  | A:ALA127:HN | Conventional Hydrogen Bond | 2.77273 |
|  |  | N:UNK1:H | Conventional Hydrogen Bond | 2.17572 |
|  |  | A:PHE124:CA | Carbon Hydrogen Bond | 3.3076 |
|  |  | N:UNK1:C | Pi-Sigma | 3.85147 |
|  |  | N:UNK1:C | Alkyl | 4.01861 |
|  |  | N:UNK1:C | Alkyl | 4.44761 |
|  |  | N:UNK1:C | Alkyl | 3.83018 |
| **6450521+**  **3U4K** | **-5.9** | A:SER75:HN | Conventional Hydrogen Bond | 2.77444 |
|  |  | A:SER75:HG | Conventional Hydrogen Bond | 2.6932 |
|  |  | A:THR119:HG1 | Conventional Hydrogen Bond | 2.12925 |
|  |  | A:ARG125:HE | Conventional Hydrogen Bond | 3.00397 |
|  |  | A:ARG125:HH22 | Conventional Hydrogen Bond | 2.43229 |
|  |  | N:UNK1:H | Conventional Hydrogen Bond | 2.56591 |
|  |  | N:UNK1:H | Conventional Hydrogen Bond | 2.32079 |
|  |  | N:UNK1:H | Conventional Hydrogen Bond | 3.05836 |
|  |  | N:UNK1:H | Conventional Hydrogen Bond | 2.7624 |
|  |  | N:UNK1:H | Conventional Hydrogen Bond | 2.82771 |
|  |  | N:UNK1:H | Conventional Hydrogen Bond | 2.65848 |
|  |  | N:UNK1:H | Conventional Hydrogen Bond | 2.88969 |
|  |  | N:UNK1:C | Carbon Hydrogen Bond | 3.38766 |
|  |  | N:UNK1:C | Alkyl | 4.03463 |
|  |  | A:TYR117 | Pi-Alkyl | 5.28212 |
|  |  | A:PHE124 | Pi-Alkyl | 5.15739 |
|  |  | A:TYR155 | Pi-Alkyl | 5.36677 |
| **6436023+**  **3U4K** | **-5.9** | A:THR132:CG2 | Pi-Sigma | 3.66947 |
|  |  | A:LYS106 | Alkyl | 4.55447 |
|  |  | A:LYS106 | Alkyl | 4.12072 |
|  |  | N:UNK1:C | Alkyl | 3.79501 |
| **6436023+**  **3U4K** | **-5.9** | A:THR132:CG2 | Pi-Sigma | 3.66947 |
|  |  | A:LYS106 | Alkyl | 4.55447 |
|  |  | A:LYS106 | Alkyl | 4.12072 |
|  |  | N:UNK1:C | Alkyl | 3.79501 |
| **52953388+**  **3U4K** | **-5.9** | A:THR119:HG1 | Conventional Hydrogen Bond | 2.88045 |
|  |  | A:TYR155:HH | Conventional Hydrogen Bond | 2.31024 |
|  |  | A:ALA127 | Alkyl | 3.63367 |
|  |  | A:ALA127 | Alkyl | 4.24551 |
|  |  | N:UNK1:C | Alkyl | 4.23323 |
|  |  | N:UNK1:C | Alkyl | 4.93904 |
|  |  | A:PHE124 | Pi-Alkyl | 4.64665 |
| **44564030+**  **3U4K** | **-5.9** | A:SER25:HG | Conventional Hydrogen Bond | 2.56932 |
|  |  | A:SER25:HG | Conventional Hydrogen Bond | 2.02607 |
|  |  | N:UNK1:C | Alkyl | 5.09534 |
|  |  | N:UNK1:C | Alkyl | 4.84539 |
|  |  | A:TRP23 | Pi-Alkyl | 5.2718 |
|  |  | A:TRP23 | Pi-Alkyl | 5.05667 |
| **44254167+**  **3U4K** | **-5.9** | A:THR138:HG1 | Conventional Hydrogen Bond | 2.68022 |
|  |  | A:GLY87:CA | Pi-Sigma | 3.78009 |
|  |  | A:THR138:CG2 | Pi-Sigma | 3.85811 |
|  |  | A:VAL91:C,O;TYR92:N | Amide-Pi Stacked | 4.88993 |
|  |  | A:ALA88 | Alkyl | 3.96271 |
|  |  | N:UNK1:C | Alkyl | 3.93416 |
|  |  | N:UNK1:C | Alkyl | 4.58015 |
|  |  | N:UNK1 | Pi-Alkyl | 4.9745 |
| **25229651+**  **3U4K** | **-5.9** | N:UNK1:H | Conventional Hydrogen Bond | 2.07853 |
|  |  | N:UNK1:H | Conventional Hydrogen Bond | 2.62262 |
|  |  | N:UNK1:H | Conventional Hydrogen Bond | 2.12885 |
|  |  | N:UNK1:C | Carbon Hydrogen Bond | 3.22126 |
|  |  | A:TYR29 | Pi-Alkyl | 5.3691 |
| **156581633+**  **3U4K** | **-5.9** | N:UNK1:H | Conventional Hydrogen Bond | 2.46225 |
|  |  | A:VAL38 | Alkyl | 5.36374 |
|  |  | A:ALA51 | Alkyl | 4.1158 |
|  |  | A:ALA51 | Alkyl | 4.10725 |
|  |  | A:LYS53 | Alkyl | 4.38417 |
|  |  | N:UNK1:C | Alkyl | 4.22241 |
|  |  | N:UNK1:C | Alkyl | 4.03621 |
|  |  | N:UNK1:C | Alkyl | 3.91205 |
|  |  | N:UNK1:C | Alkyl | 5.43463 |
|  |  | N:UNK1:C | Alkyl | 5.33839 |
| **147140349+**  **3U4K** | **-5.9** | A:SER93:HN | Conventional Hydrogen Bond | 2.42001 |
|  |  | A:THR138:HG1 | Conventional Hydrogen Bond | 2.74303 |
|  |  | A:ALA88 | Alkyl | 3.92369 |
|  |  | A:ALA88 | Alkyl | 3.81252 |
|  |  | N:UNK1:C | Alkyl | 3.87261 |
| **146682745+**  **3U4K** | **-5.9** | A:TYR117:HH | Conventional Hydrogen Bond | 2.52876 |
|  |  | A:THR119:HG1 | Conventional Hydrogen Bond | 2.45681 |
|  |  | N:UNK1:H | Conventional Hydrogen Bond | 2.46039 |
|  |  | A:THR119:CG2 | Pi-Sigma | 3.90977 |
| **139588514+**  **3U4K** | **-5.9** | A:TYR117:HH | Conventional Hydrogen Bond | 2.12249 |
|  |  | A:THR119:HG1 | Conventional Hydrogen Bond | 2.15688 |
|  |  | A:THR120:HN | Conventional Hydrogen Bond | 2.22565 |
|  |  | A:THR120:HG1 | Conventional Hydrogen Bond | 2.23534 |
|  |  | A:ARG125:HN | Conventional Hydrogen Bond | 2.32422 |
|  |  | A:ARG125:HE | Conventional Hydrogen Bond | 2.3917 |
|  |  | N:UNK1:H | Conventional Hydrogen Bond | 2.39909 |
|  |  | N:UNK1:H | Conventional Hydrogen Bond | 2.60829 |
|  |  | N:UNK1:H | Conventional Hydrogen Bond | 2.64384 |
|  |  | N:UNK1:H | Conventional Hydrogen Bond | 2.85515 |
|  |  | N:UNK1:H | Conventional Hydrogen Bond | 3.04205 |
|  |  | N:UNK1:H | Conventional Hydrogen Bond | 3.07386 |
|  |  | A:GLY121:CA | Carbon Hydrogen Bond | 3.14098 |
|  |  | N:UNK1:C | Carbon Hydrogen Bond | 3.42736 |
|  |  | A:PHE124 | Pi-Alkyl | 4.93405 |
| **13592175+**  **3U4K** | **-5.9** | A:ARG102:HH22 | Conventional Hydrogen Bond | 2.48604 |
|  |  | N:UNK1:H | Conventional Hydrogen Bond | 2.20279 |
|  |  | A:THR132:CG2 | Pi-Sigma | 3.59856 |
|  |  | A:THR132:CG2 | Pi-Sigma | 3.90985 |
|  |  | A:THR132:CG2 | Pi-Sigma | 3.65474 |
|  |  | N:UNK1 | Pi-Alkyl | 4.96172 |
| **132502750+**  **3U4K** | **-5.9** | A:ARG125:HN | Conventional Hydrogen Bond | 2.31348 |
|  |  | N:UNK1:C | Carbon Hydrogen Bond | 3.57716 |
|  |  | A:THR119:CG2 | Pi-Sigma | 3.75956 |
|  |  | N:UNK1:C | Alkyl | 4.08925 |
| **10938259+**  **3U4K** | **-5.9** | A:ARG105:HH11 | Hydrogen Bond | 2.11857 |
|  |  | A:ARG105:HH21 | Hydrogen Bond | 2.82577 |
|  |  | A:ARG125:HH22 | Hydrogen Bond | 2.4038 |
|  |  | A:GLY121:CA | Hydrogen Bond | 3.5487 |
|  |  | N:UNK1:C | Hydrophobic | 3.94701 |
|  |  | A:ARG125 | Hydrophobic | 4.49637 |
|  |  | N:UNK1:C | Hydrophobic | 3.90153 |
|  |  | N:UNK1:C | Hydrophobic | 4.51616 |
|  |  | N:UNK1:C | Hydrophobic | 3.69535 |
|  |  | A:PHE124 | Hydrophobic | 3.98403 |
| **101899578+**  **3U4K** | **-5.9** | N:UNK1:H | Conventional Hydrogen Bond | 2.20949 |
|  |  | N:UNK1:H | Conventional Hydrogen Bond | 2.79222 |
|  |  | A:THR132:CG2 | Pi-Sigma | 3.93634 |
|  |  | A:THR132:CG2 | Pi-Sigma | 3.69794 |
|  |  | A:THR132:CG2 | Pi-Sigma | 3.54458 |
|  |  | N:UNK1:C | Alkyl | 3.66773 |
|  |  | N:UNK1:C | Alkyl | 4.59321 |
|  |  | N:UNK1 | Pi-Alkyl | 4.8278 |
| **9984290+**  **3U4K** | **-5.8** | N:UNK1:O | Conventional Hydrogen Bond | 2.81328 |
|  |  | N:UNK1:O | Conventional Hydrogen Bond | 2.29392 |
|  |  | N:UNK1:O | Conventional Hydrogen Bond | 2.71989 |
|  |  | A:ASP59:OD1 | Carbon Hydrogen Bond | 3.70175 |
|  |  | A:ASP59:OD2 | Carbon Hydrogen Bond | 3.74702 |
|  |  | N:UNK1 | Pi-Sigma | 3.76743 |
|  |  | N:UNK1 | Pi-Sigma | 3.78655 |
|  |  | N:UNK1:C | Alkyl | 4.45034 |
|  |  | N:UNK1:C | Alkyl | 3.80213 |
|  |  | N:UNK1:C | Alkyl | 3.66956 |
|  |  | A:ARG125 | Alkyl | 4.24746 |
|  |  | N:UNK1:C | Pi-Alkyl | 4.61315 |
|  |  | A:ALA127 | Pi-Alkyl | 4.56226 |
|  |  | A:ARG125 | Pi-Alkyl | 5.18306 |
| **78210124+**  **3U4K** | **-5.8** | A:ARG125:HN | Hydrogen Bond | 1.88203 |
|  |  | A:ARG125:HE | Hydrogen Bond | 2.21025 |
|  |  | A:ARG125:HH22 | Hydrogen Bond | 2.29712 |
|  |  | N:UNK1:H | Hydrogen Bond | 2.87207 |
|  |  | N:UNK1:C | Hydrophobic | 3.88526 |
|  |  | N:UNK1:C | Hydrophobic | 4.12688 |
|  |  | N:UNK1:C | Hydrophobic | 4.12057 |
|  |  | N:UNK1:C | Hydrophobic | 5.11784 |
|  |  | A:PHE124 | Hydrophobic | 4.52695 |
| **6478915+**  **3U4K** | **-5.8** | A:TYR117:HH | Conventional Hydrogen Bond | 2.09893 |
|  |  | A:ARG125:HN | Conventional Hydrogen Bond | 2.01444 |
|  |  | N:UNK1:H | Conventional Hydrogen Bond | 1.8873 |
|  |  | A:THR119:CG2 | Pi-Sigma | 3.68896 |
| **54685489+**  **3U4K** | **-5.8** | A:THR119:HG1 | Conventional Hydrogen Bond | 1.98861 |
|  |  | N:UNK1:C | Pi-Sigma | 3.9457 |
|  |  | N:UNK1 | Alkyl | 5.21469 |
|  |  | N:UNK1:C | Alkyl | 5.17168 |
|  |  | A:TYR117 | Pi-Alkyl | 4.97296 |
|  |  | A:PHE124 | Pi-Alkyl | 4.26142 |
| **44178930+**  **3U4K** | **-5.8** | A:TYR117:HH | Conventional Hydrogen Bond | 1.84836 |
|  |  | A:THR119:HG1 | Conventional Hydrogen Bond | 2.35743 |
|  |  | A:THR119:HG1 | Conventional Hydrogen Bond | 2.53215 |
|  |  | A:SER122:HN | Conventional Hydrogen Bond | 2.20361 |
|  |  | A:SER123:HN | Conventional Hydrogen Bond | 1.94452 |
|  |  | N:UNK1:H | Conventional Hydrogen Bond | 2.57567 |
|  |  | N:UNK1:H | Conventional Hydrogen Bond | 1.92188 |
|  |  | A:GLY121:CA | Carbon Hydrogen Bond | 3.3335 |
|  |  | N:UNK1:C | Carbon Hydrogen Bond | 3.52928 |
|  |  | N:UNK1:C | Carbon Hydrogen Bond | 3.57538 |
|  |  | N:UNK1:C | Alkyl | 4.56952 |
|  |  | A:PHE124 | Pi-Alkyl | 4.72592 |
| **44178846+**  **3U4K** | **-5.8** | A:VAL79:HN | Conventional Hydrogen Bond | 2.06419 |
|  |  | A:GLN86:HE21 | Conventional Hydrogen Bond | 2.1225 |
|  |  | N:UNK1:H | Conventional Hydrogen Bond | 2.9449 |
|  |  | N:UNK1:H | Conventional Hydrogen Bond | 2.1798 |
|  |  | N:UNK1:H | Conventional Hydrogen Bond | 2.17649 |
|  |  | N:UNK1:H | Conventional Hydrogen Bond | 2.36662 |
|  |  | N:UNK1:H | Conventional Hydrogen Bond | 2.73886 |
|  |  | N:UNK1:H | Conventional Hydrogen Bond | 2.95452 |
|  |  | N:UNK1:H | Conventional Hydrogen Bond | 2.39504 |
|  |  | A:LYS78 | Alkyl | 5.22874 |
|  |  | A:VAL80 | Alkyl | 5.21468 |
|  |  | N:UNK1:C | Alkyl | 4.72467 |
| **156581634+**  **3U4K** | **-5.8** | A:GLY87:HN | Conventional Hydrogen Bond | 2.86268 |
|  |  | A:THR138:HG1 | Conventional Hydrogen Bond | 2.52123 |
|  |  | N:UNK1:H | Conventional Hydrogen Bond | 2.46389 |
|  |  | A:ALA88 | Alkyl | 4.92776 |
|  |  | A:VAL91 | Alkyl | 5.16636 |
|  |  | N:UNK1:C | Alkyl | 4.71312 |
| **156581251+**  **3U4K** | **-5.8** | A:GLY36:HN3 | Conventional Hydrogen Bond | 2.63849 |
|  |  | A:GLY36:HN2 | Conventional Hydrogen Bond | 2.69249 |
|  |  | N:UNK1:C | Carbon Hydrogen Bond | 3.27578 |
|  |  | A:ALA51 | Alkyl | 4.13968 |
|  |  | N:UNK1:C | Alkyl | 4.96544 |
|  |  | N:UNK1:C | Alkyl | 5.42376 |
|  |  | N:UNK1:C | Alkyl | 4.62184 |
| **139587751+**  **3U4K** | **-5.8** | A:TYR117:HH | Conventional Hydrogen Bond | 2.11883 |
|  |  | A:THR119:HG1 | Conventional Hydrogen Bond | 2.32022 |
|  |  | N:UNK1:H | Conventional Hydrogen Bond | 2.14674 |
|  |  | A:ALA127 | Alkyl | 4.28189 |
|  |  | N:UNK1:C | Alkyl | 3.81073 |
|  |  | N:UNK1:C | Alkyl | 5.44786 |
|  |  | A:TYR155 | Pi-Alkyl | 4.82322 |
| **139584543+**  **3U4K** | **-5.8** | A:THR119:HG1 | Hydrogen Bond | 2.10672 |
|  |  | N:UNK1:C | Hydrophobic | 3.98669 |
|  |  | N:UNK1:C | Hydrophobic | 4.28452 |
|  |  | N:UNK1:C | Hydrophobic | 4.44046 |
|  |  | N:UNK1:C | Hydrophobic | 5.05141 |
|  |  | A:PHE124 | Hydrophobic | 4.59152 |
|  |  | N:UNK1 | Hydrophobic | 4.46603 |
| **132556712+**  **3U4K** | **-5.8** | A:THR119:HG1 | Conventional Hydrogen Bond | 2.06239 |
|  |  | A:SER122:HN | Conventional Hydrogen Bond | 1.98083 |
|  |  | A:ARG125:HN | Conventional Hydrogen Bond | 2.7599 |
|  |  | N:UNK1:H | Conventional Hydrogen Bond | 2.26169 |
|  |  | N:UNK1:H | Conventional Hydrogen Bond | 2.57369 |
| **1268111+**  **3U4K** | **-5.8** | A:ARG105:HH11 | Conventional Hydrogen Bond | 2.04147 |
|  |  | A:ARG105:HH21 | Conventional Hydrogen Bond | 2.67916 |
|  |  | N:UNK1:C | Carbon Hydrogen Bond | 3.57351 |
| **11471114+**  **3U4K** | **-5.8** | A:ARG125:HN | Conventional Hydrogen Bond | 2.41623 |
|  |  | A:GLY121:CA | Carbon Hydrogen Bond | 3.71188 |
|  |  | A:PHE124 | Pi-Alkyl | 5.39552 |
| **10573163+**  **3U4K** | **-5.8** | A:SER93:HG | Conventional Hydrogen Bond | 2.05808 |
|  |  | N:UNK1:H | Conventional Hydrogen Bond | 2.52497 |
|  |  | N:UNK1:H | Conventional Hydrogen Bond | 2.88485 |
|  |  | A:GLY87:CA | Carbon Hydrogen Bond | 3.54984 |
|  |  | A:SER93:CB | Carbon Hydrogen Bond | 3.71078 |
|  |  | A:ALA88:HN | Pi-Donor Hydrogen Bond | 3.19721 |
|  |  | N:UNK1 | Pi-Alkyl | 4.67046 |
|  |  | N:UNK1 | Pi-Alkyl | 5.21444 |
| **102527976+**  **3U4K** | **-5.8** | N:UNK1:C | Alkyl | 3.81388 |
|  |  | N:UNK1:C | Alkyl | 5.30476 |
|  |  | A:PHE124 | Pi-Alkyl | 4.27932 |
| **101899578+**  **3U4K** | **-5.8** | N:UNK1:H | Conventional Hydrogen Bond | 2.20949 |
|  |  | N:UNK1:H | Conventional Hydrogen Bond | 2.79222 |
|  |  | A:THR132:CG2 | Pi-Sigma | 3.69794 |
|  |  | A:THR132:CG2 | Pi-Sigma | 3.93634 |
|  |  | A:THR132:CG2 | Pi-Sigma | 3.54458 |
|  |  | N:UNK1:C | Alkyl | 3.66773 |
|  |  | N:UNK1:C | Alkyl | 4.59321 |
|  |  | N:UNK1 | Pi-Alkyl | 4.8278 |
| **9965029+**  **3U4K** | **-5.7** | A:TYR117:HH | Conventional Hydrogen Bond | 2.25355 |
|  |  | A:ALA127:HN | Conventional Hydrogen Bond | 3.00441 |
|  |  | N:UNK1 | Pi-Alkyl | 5.36128 |
| **7074739+**  **3U4K** | **-5.7** | A:ARG125:HN | Conventional Hydrogen Bond | 2.1982 |
|  |  | N:UNK1:H | Conventional Hydrogen Bond | 2.03207 |
|  |  | A:PHE124 | Pi-Alkyl | 5.13985 |
| **6478915+**  **3U4K** | **-5.7** | A:TYR117:HH | Conventional Hydrogen Bond | 2.09893 |
|  |  | A:ARG125:HN | Conventional Hydrogen Bond | 2.01444 |
|  |  | N:UNK1:H | Conventional Hydrogen Bond | 1.8873 |
|  |  | A:THR119:CG2 | Pi-Sigma | 3.68896 |
| **6450521+**  **3U4K** | **-5.7** | A:SER75:HN | Conventional Hydrogen Bond | 2.77444 |
|  |  | A:SER75:HG | Conventional Hydrogen Bond | 2.6932 |
|  |  | A:THR119:HG1 | Conventional Hydrogen Bond | 2.12925 |
|  |  | A:ARG125:HE | Conventional Hydrogen Bond | 3.00397 |
|  |  | A:ARG125:HH22 | Conventional Hydrogen Bond | 2.43229 |
|  |  | N:UNK1:H | Conventional Hydrogen Bond | 2.56591 |
|  |  | N:UNK1:H | Conventional Hydrogen Bond | 2.32079 |
|  |  | N:UNK1:H | Conventional Hydrogen Bond | 3.05836 |
|  |  | N:UNK1:H | Conventional Hydrogen Bond | 2.7624 |
|  |  | N:UNK1:H | Conventional Hydrogen Bond | 2.82771 |
|  |  | N:UNK1:H | Conventional Hydrogen Bond | 2.65848 |
|  |  | N:UNK1:H | Conventional Hydrogen Bond | 2.88969 |
|  |  | N:UNK1:C | Carbon Hydrogen Bond | 3.38766 |
|  |  | N:UNK1:C | Alkyl | 4.03463 |
|  |  | A:TYR117 | Pi-Alkyl | 5.28212 |
|  |  | A:PHE124 | Pi-Alkyl | 5.15739 |
|  |  | A:TYR155 | Pi-Alkyl | 5.36677 |
| **6450520+**  **3U4K** | **-5.7** | A:SER75:HG | Conventional Hydrogen Bond | 2.0918 |
|  |  | A:ARG105:HH21 | Conventional Hydrogen Bond | 2.70104 |
|  |  | A:THR119:HG1 | Conventional Hydrogen Bond | 2.79083 |
|  |  | A:THR120:HN | Conventional Hydrogen Bond | 2.05877 |
|  |  | A:TYR155:HH | Conventional Hydrogen Bond | 2.31713 |
|  |  | N:UNK1:H | Conventional Hydrogen Bond | 2.51747 |
|  |  | N:UNK1:H | Conventional Hydrogen Bond | 2.41619 |
|  |  | N:UNK1:H | Conventional Hydrogen Bond | 2.06642 |
|  |  | N:UNK1:H | Conventional Hydrogen Bond | 2.76865 |
|  |  | N:UNK1:H | Conventional Hydrogen Bond | 2.25246 |
|  |  | N:UNK1:H | Conventional Hydrogen Bond | 2.72325 |
|  |  | A:ARG125 | Alkyl | 4.95359 |
|  |  | A:ALA127 | Alkyl | 4.49981 |
|  |  | N:UNK1:C | Alkyl | 4.43124 |
|  |  | N:UNK1 | Alkyl | 4.88667 |
|  |  | N:UNK1:C | Alkyl | 4.37505 |
|  |  | N:UNK1:C | Alkyl | 4.29028 |
|  |  | N:UNK1:C | Alkyl | 5.11855 |
|  |  | A:TYR117 | Pi-Alkyl | 4.8748 |
|  |  | A:PHE124 | Pi-Alkyl | 4.63844 |
| **6437365+**  **3U4K** | **-5.7** | A:GLN86:HN | Conventional Hydrogen Bond | 2.33184 |
|  |  | N:UNK1:C | Alkyl | 4.5045 |
|  |  | N:UNK1:C | Alkyl | 4.41487 |
|  |  | N:UNK1:C | Alkyl | 4.38124 |
|  |  | N:UNK1:C | Alkyl | 4.91886 |
| **57339223+**  **3U4K** | **-5.7** | N:UNK1:C | Carbon Hydrogen Bond | 3.01545 |
|  |  | A:THR132:CG2 | Pi-Sigma | 3.80725 |
|  |  | A:THR132:CG2 | Pi-Sigma | 3.44429 |
|  |  | N:UNK1:C | Alkyl | 4.94216 |
|  |  | N:UNK1:C | Alkyl | 4.52214 |
|  |  | N:UNK1:C | Alkyl | 3.88154 |
|  |  | N:UNK1:C | Alkyl | 5.31818 |
| **46871990+**  **3U4K** | **-5.7** | A:TYR117:HH | Conventional Hydrogen Bond | 2.15261 |
|  |  | A:TYR155:HH | Conventional Hydrogen Bond | 2.27624 |
|  |  | A:THR119:CG2 | Pi-Sigma | 3.77184 |
|  |  | N:UNK1:C | Alkyl | 3.94811 |
|  |  | A:PHE124 | Pi-Alkyl | 4.6669 |
| **44231743+**  **3U4K** | **-5.7** | N:UNK1:H | Conventional Hydrogen Bond | 2.52914 |
|  |  | A:THR132:CG2 | Pi-Sigma | 3.80625 |
|  |  | N:UNK1:C | Alkyl | 4.25812 |
|  |  | N:UNK1 | Pi-Alkyl | 5.47598 |
| **44178844+**  **3U4K** | **-5.7** | A:ASN70:HD22 | Conventional Hydrogen Bond | 2.59526 |
|  |  | A:SER122:HN | Conventional Hydrogen Bond | 1.90208 |
|  |  | A:SER122:HG | Conventional Hydrogen Bond | 1.99706 |
|  |  | A:SER122:HG | Conventional Hydrogen Bond | 2.74075 |
|  |  | A:SER123:HN | Conventional Hydrogen Bond | 1.85123 |
|  |  | A:ARG125:HE | Conventional Hydrogen Bond | 2.89361 |
|  |  | A:ARG125:HH11 | Conventional Hydrogen Bond | 2.51145 |
|  |  | A:ARG125:HH22 | Conventional Hydrogen Bond | 2.74142 |
|  |  | N:UNK1:H | Conventional Hydrogen Bond | 2.21835 |
|  |  | N:UNK1:H | Conventional Hydrogen Bond | 2.41215 |
|  |  | N:UNK1:C | Carbon Hydrogen Bond | 3.47485 |
|  |  | A:PHE124 | Pi-Alkyl | 4.84539 |
| **25022542+**  **3U4K** | **-5.7** | A:TYR117:HH | Conventional Hydrogen Bond | 2.84705 |
|  |  | N:UNK1:H | Conventional Hydrogen Bond | 2.27838 |
|  |  | N:UNK1:H | Conventional Hydrogen Bond | 1.82269 |
|  |  | N:UNK1:H | Conventional Hydrogen Bond | 2.40143 |
|  |  | A:ALA127 | Alkyl | 4.25723 |
|  |  | A:ALA127 | Alkyl | 3.70255 |
|  |  | N:UNK1:C | Alkyl | 4.24413 |
|  |  | N:UNK1:C | Alkyl | 4.82747 |
|  |  | N:UNK1:C | Alkyl | 3.7986 |
| **156581631+**  **3U4K** | **-5.7** | A:ARG125:HN | Conventional Hydrogen Bond | 2.93414 |
|  |  | N:UNK1:H | Conventional Hydrogen Bond | 2.42259 |
|  |  | N:UNK1:H | Conventional Hydrogen Bond | 1.79744 |
|  |  | A:ALA108 | Alkyl | 4.2063 |
|  |  | A:ALA127 | Alkyl | 3.88901 |
|  |  | N:UNK1:C | Alkyl | 5.17311 |
|  |  | A:PHE124 | Pi-Alkyl | 4.54047 |
| **139590672+**  **3U4K** | **-5.7** | A:PRO58 | Alkyl | 5.4067 |
|  |  | N:UNK1 | Alkyl | 5.16287 |
|  |  | N:UNK1:C | Alkyl | 4.54896 |
|  |  | N:UNK1:C | Alkyl | 4.43846 |
|  |  | A:TYR29 | Pi-Alkyl | 3.64097 |
|  |  | A:PHE131 | Pi-Alkyl | 5.26817 |
|  |  | N:UNK1 | Pi-Alkyl | 5.40512 |
|  |  | N:UNK1 | Pi-Alkyl | 5.14877 |
| **139590671+**  **3U4K** | **-5.7** | A:TYR117:HH | Conventional Hydrogen Bond | 2.79062 |
|  |  | A:ALA127:HN | Conventional Hydrogen Bond | 2.67851 |
|  |  | A:TYR155:HH | Conventional Hydrogen Bond | 2.16371 |
|  |  | A:ALA108 | Alkyl | 3.89981 |
|  |  | A:ALA127 | Alkyl | 3.90937 |
|  |  | N:UNK1:C | Alkyl | 4.51612 |
|  |  | N:UNK1:C | Alkyl | 4.7489 |
|  |  | A:PHE124 | Pi-Alkyl | 4.97054 |
|  |  | A:TYR155 | Pi-Alkyl | 5.15139 |
| **139589569+**  **3U4K** | **-5.7** | A:SER75:HG | Conventional Hydrogen Bond | 2.46454 |
|  |  | A:THR119:HG1 | Pi-Donor Hydrogen Bond | 3.08534 |
|  |  | A:THR119:CG2 | Pi-Sigma | 3.74879 |
|  |  | A:PHE124 | Pi-Alkyl | 5.03815 |
| **139588514+**  **3U4K** | **-5.7** | A:TYR117:HH | Conventional Hydrogen Bond | 2.32422 |
|  |  | A:THR119:HG1 | Conventional Hydrogen Bond | 2.3917 |
|  |  | A:THR120:HN | Conventional Hydrogen Bond | 2.22565 |
|  |  | A:THR120:HG1 | Conventional Hydrogen Bond | 2.60829 |
|  |  | A:ARG125:HN | Conventional Hydrogen Bond | 2.39909 |
|  |  | A:ARG125:HE | Conventional Hydrogen Bond | 2.85515 |
|  |  | N:UNK1:H | Conventional Hydrogen Bond | 2.23534 |
|  |  | N:UNK1:H | Conventional Hydrogen Bond | 3.04205 |
|  |  | N:UNK1:H | Conventional Hydrogen Bond | 2.15688 |
|  |  | N:UNK1:H | Conventional Hydrogen Bond | 2.64384 |
|  |  | N:UNK1:H | Conventional Hydrogen Bond | 3.07386 |
|  |  | N:UNK1:H | Conventional Hydrogen Bond | 2.12249 |
|  |  | A:GLY121:CA | Carbon Hydrogen Bond | 3.42736 |
|  |  | N:UNK1:C | Carbon Hydrogen Bond | 3.14098 |
|  |  | A:PHE124 | Pi-Alkyl | 4.93405 |
| **139583971+**  **3U4K** | **-5.7** | A:ARG125:HN | Conventional Hydrogen Bond | 2.46014 |
|  |  | A:ARG125:HE | Conventional Hydrogen Bond | 2.95703 |
|  |  | A:ARG125:HH22 | Conventional Hydrogen Bond | 2.99728 |
|  |  | A:PHE124 | Pi-Alkyl | 5.26283 |
| **10938259+**  **3U4K** | **-5.7** | A:ARG105:HH11 | Conventional Hydrogen Bond | 2.11857 |
|  |  | A:ARG105:HH21 | Conventional Hydrogen Bond | 2.82577 |
|  |  | A:ARG125:HH22 | Conventional Hydrogen Bond | 2.4038 |
|  |  | A:GLY121:CA | Carbon Hydrogen Bond | 3.5487 |
|  |  | N:UNK1:C | Pi-Sigma | 3.94701 |
|  |  | A:ARG125 | Alkyl | 4.49637 |
|  |  | N:UNK1:C | Alkyl | 3.90153 |
|  |  | N:UNK1:C | Alkyl | 4.51616 |
|  |  | N:UNK1:C | Alkyl | 3.69535 |
|  |  | A:PHE124 | Pi-Alkyl | 3.98403 |
| **10515747+**  **3U4K** | **-5.7** | A:ALA127:HN | Conventional Hydrogen Bond | 2.71198 |
|  |  | N:UNK1:H | Conventional Hydrogen Bond | 2.40395 |
|  |  | A:ALA108 | Alkyl | 3.76179 |
|  |  | A:ALA127 | Alkyl | 4.06963 |
|  |  | N:UNK1:C | Alkyl | 5.13746 |
|  |  | A:PHE124 | Pi-Alkyl | 4.60757 |
|  |  | A:TYR155 | Pi-Alkyl | 4.60796 |
| **10376935+**  **3U4K** | **-5.7** | A:ARG125:HN | Conventional Hydrogen Bond | 2.49554 |
|  |  | N:UNK1:C | Alkyl | 4.0505 |
| **10038050+**  **3U4K** | **-5.7** | A:ARG125:HE | Conventional Hydrogen Bond | 2.64406 |
|  |  | A:ARG125:HH22 | Conventional Hydrogen Bond | 2.97229 |
|  |  | N:UNK1:C | Alkyl | 4.94689 |
|  |  | A:TYR117 | Pi-Alkyl | 5.18359 |
| **6917655+**  **3U4K** | **-5.6** | A:SER123:HN | Conventional Hydrogen Bond | 1.96474 |
|  |  | A:ALA127 | Alkyl | 4.60323 |
|  |  | A:TYR117 | Pi-Alkyl | 5.1768 |
|  |  | A:PHE124 | Pi-Alkyl | 5.07108 |
|  |  | A:TYR155 | Pi-Alkyl | 5.27476 |
| **6223+**  **3U4K** | **-5.6** | A:THR119:HG1 | Conventional Hydrogen Bond | 2.04078 |
|  |  | A:ARG125:HN | Conventional Hydrogen Bond | 2.00032 |
|  |  | A:ARG125:HN | Conventional Hydrogen Bond | 2.32445 |
|  |  | N:UNK1:C | Alkyl | 5.09546 |
|  |  | A:TYR117 | Pi-Alkyl | 4.94183 |
| **44178930+**  **3U4K** | **-5.6** | A:TYR117:HH | Conventional Hydrogen Bond | 1.84836 |
|  |  | A:THR119:HG1 | Conventional Hydrogen Bond | 2.35743 |
|  |  | A:THR119:HG1 | Conventional Hydrogen Bond | 2.53215 |
|  |  | A:SER122:HN | Conventional Hydrogen Bond | 2.20361 |
|  |  | A:SER123:HN | Conventional Hydrogen Bond | 1.94452 |
|  |  | N:UNK1:H | Conventional Hydrogen Bond | 2.57567 |
|  |  | N:UNK1:H | Conventional Hydrogen Bond | 1.92188 |
|  |  | A:GLY121:CA | Carbon Hydrogen Bond | 3.3335 |
|  |  | N:UNK1:C | Carbon Hydrogen Bond | 3.52928 |
|  |  | N:UNK1:C | Carbon Hydrogen Bond | 3.57538 |
|  |  | N:UNK1:C | Alkyl | 4.56952 |
|  |  | A:PHE124 | Pi-Alkyl | 4.72592 |
| **21596370+**  **3U4K** | **-5.6** | A:ARG125:HN | Conventional Hydrogen Bond | 2.37914 |
|  |  | N:UNK1:H | Conventional Hydrogen Bond | 2.65309 |
|  |  | N:UNK1:H | Conventional Hydrogen Bond | 1.98209 |
|  |  | N:UNK1:C | Alkyl | 4.41312 |
|  |  | N:UNK1 | Alkyl | 5.0249 |
|  |  | A:TYR117 | Pi-Alkyl | 5.42894 |
|  |  | A:PHE124 | Pi-Alkyl | 5.44305 |
|  |  | A:PHE124 | Pi-Alkyl | 4.56902 |
| **156581632+**  **3U4K** | **-5.6** | N:UNK1:H | Conventional Hydrogen Bond | 2.04663 |
|  |  | A:GLY121:CA | Carbon Hydrogen Bond | 3.65971 |
|  |  | A:ALA127 | Alkyl | 3.86491 |
|  |  | A:PHE124 | Pi-Alkyl | 4.96981 |
| **15265874+**  **3U4K** | **-5.6** | A:TYR117:HH | Conventional Hydrogen Bond | 2.27372 |
|  |  | A:THR119:HG1 | Conventional Hydrogen Bond | 2.18253 |
|  |  | A:ALA127:HN | Conventional Hydrogen Bond | 2.61468 |
|  |  | A:ALA127 | Alkyl | 3.61701 |
|  |  | N:UNK1:C | Alkyl | 4.43869 |
|  |  | N:UNK1:C | Alkyl | 4.91615 |
|  |  | A:TYR155 | Pi-Alkyl | 5.06561 |
|  |  | A:TYR155 | Pi-Alkyl | 5.11026 |
| **148772264+**  **3U4K** | **-5.6** | A:VAL38 | Hydrophobic | 5.21964 |
|  |  | A:ALA51 | Hydrophobic | 4.37738 |
|  |  | A:ALA51 | Hydrophobic | 3.63198 |
|  |  | A:ALA51 | Hydrophobic | 3.90254 |
|  |  | N:UNK1:C | Hydrophobic | 4.13955 |
|  |  | N:UNK1:C | Hydrophobic | 5.39225 |
|  |  | N:UNK1:C | Hydrophobic | 4.78991 |
|  |  | N:UNK1:C | Hydrophobic | 3.99103 |
| **139590665+**  **3U4K** | **-5.6** | A:TYR117:HH | Conventional Hydrogen Bond | 1.94241 |
|  |  | A:THR119:HG1 | Conventional Hydrogen Bond | 2.27349 |
|  |  | A:ILE109:CD | Pi-Sigma | 3.72722 |
|  |  | A:TYR117 | Pi-Alkyl | 5.43463 |
|  |  | N:UNK1 | Pi-Alkyl | 5.45312 |
| **11750429+**  **3U4K** | **-5.6** | A:THR119:HG1 | Conventional Hydrogen Bond | 2.37812 |
|  |  | A:SER122:HN | Conventional Hydrogen Bond | 2.07331 |
|  |  | A:SER123:HN | Conventional Hydrogen Bond | 2.0298 |
|  |  | N:UNK1:H | Conventional Hydrogen Bond | 2.0633 |
|  |  | N:UNK1:H | Conventional Hydrogen Bond | 2.16017 |
|  |  | N:UNK1:H | Conventional Hydrogen Bond | 2.38578 |
|  |  | A:ARG125 | Alkyl | 4.49891 |
|  |  | A:ALA127 | Alkyl | 5.26472 |
|  |  | A:ALA127 | Alkyl | 4.81847 |
|  |  | N:UNK1 | Alkyl | 5.24201 |
|  |  | N:UNK1:C | Alkyl | 4.04644 |
|  |  | N:UNK1:C | Alkyl | 4.48956 |
|  |  | N:UNK1:C | Alkyl | 3.88673 |
|  |  | A:PHE124 | Pi-Alkyl | 3.82934 |
| **10376935+**  **3U4K** | **-5.6** | A:ARG125:HN | Conventional Hydrogen Bond | 2.49554 |
|  |  | N:UNK1:C | Alkyl | 4.0505 |
| **6428988+**  **3U4K** | **-5.5** | A:GLY36:HN3 | Conventional Hydrogen Bond | 2.35116 |
|  |  | N:UNK1:H | Conventional Hydrogen Bond | 2.58927 |
|  |  | A:ALA51 | Alkyl | 3.93304 |
|  |  | N:UNK1:C | Alkyl | 4.85453 |
|  |  | N:UNK1:C | Alkyl | 4.60557 |
|  |  | N:UNK1:C | Alkyl | 4.76648 |
|  |  | A:TRP55 | Pi-Alkyl | 5.41144 |
| **637398+**  **3U4K** | **-5.5** | A:THR142:HN | Conventional Hydrogen Bond | 2.14668 |
|  |  | A:THR142:HN | Conventional Hydrogen Bond | 2.27544 |
|  |  | N:UNK1:H | Conventional Hydrogen Bond | 1.94779 |
|  |  | N:UNK1:H | Conventional Hydrogen Bond | 2.41365 |
|  |  | N:UNK1:C | Alkyl | 3.79124 |
|  |  | N:UNK1:C | Alkyl | 4.43544 |
| **6223+**  **3U4K** | **-5.5** | A:ARG125:HN | Conventional Hydrogen Bond | 2.21052 |
|  |  | A:ARG125:HN | Conventional Hydrogen Bond | 2.23299 |
|  |  | N:UNK1:C | Carbon Hydrogen Bond | 3.42572 |
| **53483973+**  **3U4K** | **-5.5** | A:THR119:HG1 | Conventional Hydrogen Bond | 2.02341 |
|  |  | N:UNK1:H | Conventional Hydrogen Bond | 2.65021 |
|  |  | N:UNK1:C | Pi-Sigma | 3.87861 |
|  |  | N:UNK1 | Alkyl | 4.79237 |
|  |  | A:TYR117 | Pi-Alkyl | 5.23839 |
|  |  | A:PHE124 | Pi-Alkyl | 4.39052 |
| **52953426+**  **3U4K** | **-5.5** | A:SER75:HG | Conventional Hydrogen Bond | 2.45021 |
|  |  | A:TYR155:HH | Conventional Hydrogen Bond | 2.41009 |
|  |  | A:ALA127 | Alkyl | 3.41535 |
|  |  | A:PHE124 | Pi-Alkyl | 5.11146 |
|  |  | A:PHE124 | Pi-Alkyl | 4.37889 |
|  |  | N:UNK1 | Pi-Alkyl | 5.28525 |
| **44254252+**  **3U4K** | **-5.5** | N:UNK1:H | Conventional Hydrogen Bond | 2.88199 |
|  |  | A:THR119:HG1 | Pi-Donor Hydrogen Bond | 2.96566 |
|  |  | A:ALA108 | Alkyl | 4.30178 |
|  |  | A:ALA127 | Alkyl | 3.44226 |
|  |  | A:TYR117 | Pi-Alkyl | 4.98111 |
|  |  | A:PHE124 | Pi-Alkyl | 4.83046 |
| **40604+**  **3U4K** | **-5.5** | A:SER104:HG | Conventional Hydrogen Bond | 2.89669 |
|  |  | N:UNK1:O | Conventional Hydrogen Bond | 3.05131 |
|  |  | N:UNK1:H | Conventional Hydrogen Bond | 2.4865 |
|  |  | N:UNK1:C | Alkyl | 4.69379 |
|  |  | N:UNK1 | Pi-Alkyl | 4.65102 |
|  |  | N:UNK1 | Pi-Alkyl | 5.15268 |
| **21606643+**  **3U4K** | **-5.5** | A:TYR155:HH | Conventional Hydrogen Bond | 2.56695 |
|  |  | A:ALA127 | Alkyl | 5.03557 |
|  |  | A:TYR117 | Pi-Alkyl | 5.02078 |
|  |  | A:PHE124 | Pi-Alkyl | 5.43253 |
| **162642186+**  **3U4K** | **-5.5** | A:GLY98:HN | Conventional Hydrogen Bond | 2.32807 |
|  |  | N:UNK1:H | Conventional Hydrogen Bond | 2.34758 |
|  |  | A:VAL85 | Alkyl | 4.56534 |
|  |  | A:ALA88 | Alkyl | 4.00732 |
|  |  | A:ALA88 | Alkyl | 3.90343 |
|  |  | A:VAL91 | Alkyl | 4.35412 |
|  |  | A:VAL91 | Alkyl | 4.26846 |
|  |  | A:VAL91 | Alkyl | 5.29533 |
|  |  | N:UNK1 | Alkyl | 4.85907 |
|  |  | N:UNK1:C | Alkyl | 4.30001 |
| **148772264+**  **3U4K** | **-5.5** | A:VAL38 | Alkyl | 5.21964 |
|  |  | A:ALA51 | Alkyl | 4.37738 |
|  |  | A:ALA51 | Alkyl | 3.63198 |
|  |  | A:ALA51 | Alkyl | 3.90254 |
|  |  | N:UNK1:C | Alkyl | 4.13955 |
|  |  | N:UNK1:C | Alkyl | 5.39225 |
|  |  | N:UNK1:C | Alkyl | 4.78991 |
|  |  | N:UNK1:C | Alkyl | 3.99103 |
| **139590665+**  **3U4K** | **-5.5** | A:TYR117:HH | Conventional Hydrogen Bond | 1.94241 |
|  |  | A:THR119:HG1 | Conventional Hydrogen Bond | 2.27349 |
|  |  | A:ILE109:CD | Pi-Sigma | 3.72722 |
|  |  | A:TYR117 | Pi-Alkyl | 5.43463 |
|  |  | N:UNK1 | Pi-Alkyl | 5.45312 |
| **10573163+**  **3U4K** | **-5.5** | A:THR119:HG1 | Conventional Hydrogen Bond | 2.85217 |
|  |  | A:SER122:HN | Conventional Hydrogen Bond | 2.04839 |
|  |  | A:ARG125:HN | Conventional Hydrogen Bond | 3.04958 |
|  |  | A:ARG125:HE | Conventional Hydrogen Bond | 2.31688 |
|  |  | A:ARG125:HH22 | Conventional Hydrogen Bond | 2.13041 |
|  |  | N:UNK1:H | Conventional Hydrogen Bond | 2.33173 |
|  |  | N:UNK1:C | Alkyl | 4.58375 |
|  |  | A:TYR117 | Pi-Alkyl | 5.21949 |
| **10393+**  **3U4K** | **-5.5** | A:SER75:HG | Pi-Donor Hydrogen Bond | 3.00063 |
|  |  | A:PHE124 | Pi-Pi Stacked | 5.23202 |
| **49780759+**  **3U4K** | **-5.4** | A:THR119:HG1 | Conventional Hydrogen Bond | 2.56557 |
|  |  | A:ARG125:HH11 | Conventional Hydrogen Bond | 2.98807 |
|  |  | N:UNK1:H | Conventional Hydrogen Bond | 2.53471 |
|  |  | N:UNK1:C | Alkyl | 3.83695 |
|  |  | N:UNK1 | Pi-Alkyl | 4.48045 |
|  |  | N:UNK1 | Pi-Alkyl | 5.02977 |
| **24829258+**  **3U4K** | **-5.4** | A:TYR117:HH | Conventional Hydrogen Bond | 2.20273 |
|  |  | A:THR119:HG1 | Conventional Hydrogen Bond | 2.68316 |
|  |  | A:ARG125:HN | Conventional Hydrogen Bond | 2.0841 |
|  |  | A:ARG125:HH22 | Conventional Hydrogen Bond | 2.44292 |
|  |  | N:UNK1:H | Conventional Hydrogen Bond | 2.20067 |
|  |  | A:PHE124:CA | Carbon Hydrogen Bond | 3.36369 |
|  |  | N:UNK1:C | Alkyl | 4.4558 |
|  |  | N:UNK1:C | Alkyl | 5.0414 |
|  |  | A:TYR117 | Pi-Alkyl | 4.93934 |
| **21606643+**  **3U4K** | **-5.4** | A:TYR155:HH | Conventional Hydrogen Bond | 2.56695 |
|  |  | A:ALA127 | Alkyl | 5.03557 |
|  |  | A:TYR117 | Pi-Alkyl | 5.02078 |
|  |  | A:PHE124 | Pi-Alkyl | 5.43253 |
| **155735+**  **3U4K** | **-5.4** | A:THR142:HN | Conventional Hydrogen Bond | 2.04267 |
|  |  | A:CYS178:HN | Conventional Hydrogen Bond | 2.36069 |
|  |  | A:CYS178:HN | Conventional Hydrogen Bond | 1.91916 |
|  |  | N:UNK1:H | Conventional Hydrogen Bond | 2.41021 |
|  |  | N:UNK1 | Pi-Alkyl | 5.18175 |
| **148214470+**  **3U4K** | **-5.4** | N:UNK1:C | Alkyl | 4.23147 |
|  |  | A:TYR117 | Pi-Alkyl | 4.95612 |
|  |  | A:PHE124 | Pi-Alkyl | 4.83504 |
| **147992277+**  **3U4K** | **-5.4** | A:ARG125:HN | Conventional Hydrogen Bond | 2.21571 |
|  |  | A:TYR155:HH | Conventional Hydrogen Bond | 2.12021 |
|  |  | A:ALA127 | Alkyl | 3.85352 |
|  |  | N:UNK1:C | Alkyl | 5.05243 |
|  |  | A:PHE124 | Pi-Alkyl | 5.30183 |
|  |  | A:PHE124 | Pi-Alkyl | 4.78208 |
|  |  | A:TYR155 | Pi-Alkyl | 4.89604 |
| **11750429+**  **3U4K** | **-5.4** | A:THR119:HG1 | Conventional Hydrogen Bond | 2.37812 |
|  |  | A:SER122:HN | Conventional Hydrogen Bond | 2.07331 |
|  |  | A:SER123:HN | Conventional Hydrogen Bond | 2.0298 |
|  |  | N:UNK1:H | Conventional Hydrogen Bond | 2.0633 |
|  |  | N:UNK1:H | Conventional Hydrogen Bond | 2.16017 |
|  |  | N:UNK1:H | Conventional Hydrogen Bond | 2.38578 |
|  |  | A:ARG125 | Alkyl | 4.49891 |
|  |  | A:ALA127 | Alkyl | 5.26472 |
|  |  | A:ALA127 | Alkyl | 4.81847 |
|  |  | N:UNK1 | Alkyl | 5.24201 |
|  |  | N:UNK1:C | Alkyl | 4.04644 |
|  |  | N:UNK1:C | Alkyl | 4.48956 |
|  |  | N:UNK1:C | Alkyl | 3.88673 |
|  |  | A:PHE124 | Pi-Alkyl | 3.82934 |
| **10445511+**  **3U4K** | **-5.4** | A:CYS178 | Alkyl | 4.60887 |
|  |  | N:UNK1:C | Alkyl | 5.2807 |
|  |  | N:UNK1:C | Alkyl | 3.78863 |
|  |  | N:UNK1:C | Alkyl | 4.45631 |
| **44178748+**  **3U4K** | **-5.3** | A:THR119:HG1 | Conventional Hydrogen Bond | 2.22281 |
|  |  | A:THR119:HG1 | Conventional Hydrogen Bond | 2.50379 |
|  |  | A:ARG125:HN | Conventional Hydrogen Bond | 2.39427 |
|  |  | A:ARG125:HE | Conventional Hydrogen Bond | 2.81441 |
|  |  | A:ARG125:HH22 | Conventional Hydrogen Bond | 2.32687 |
|  |  | N:UNK1:H | Conventional Hydrogen Bond | 2.6605 |
|  |  | N:UNK1:H | Conventional Hydrogen Bond | 2.27309 |
|  |  | N:UNK1:H | Conventional Hydrogen Bond | 3.02366 |
|  |  | N:UNK1:H | Conventional Hydrogen Bond | 2.79259 |
|  |  | N:UNK1:H | Conventional Hydrogen Bond | 2.39173 |
|  |  | N:UNK1:H | Conventional Hydrogen Bond | 3.03338 |
|  |  | N:UNK1:H | Conventional Hydrogen Bond | 2.31218 |
|  |  | A:ILE109:CA | Carbon Hydrogen Bond | 3.38463 |
|  |  | N:UNK1:C | Carbon Hydrogen Bond | 3.49837 |
|  |  | A:PHE124 | Pi-Alkyl | 4.49047 |
| **156581632+**  **3U4K** | **-5.3** | N:UNK1:H | Conventional Hydrogen Bond | 2.04663 |
|  |  | A:GLY121:CA | Carbon Hydrogen Bond | 3.65971 |
|  |  | A:ALA127 | Alkyl | 3.86491 |
|  |  | A:PHE124 | Pi-Alkyl | 4.96981 |
| **139591336+**  **3U4K** | **-5.3** | A:THR142:HN | Conventional Hydrogen Bond | 2.0852 |
|  |  | N:UNK1:H | Conventional Hydrogen Bond | 2.1824 |
|  |  | N:UNK1:H | Conventional Hydrogen Bond | 2.41765 |
|  |  | N:UNK1:C | Carbon Hydrogen Bond | 3.55204 |
|  |  | N:UNK1:C | Alkyl | 4.2819 |
| **132556714+**  **3U4K** | **-5.3** | A:ALA88:HN | Conventional Hydrogen Bond | 2.59522 |
|  |  | N:UNK1:H | Conventional Hydrogen Bond | 1.95677 |
|  |  | N:UNK1:H | Conventional Hydrogen Bond | 2.33658 |
|  |  | A:GLY87:CA | Carbon Hydrogen Bond | 2.82069 |
|  |  | A:ALA88 | Alkyl | 4.46906 |
|  |  | A:ALA88 | Alkyl | 3.70894 |
|  |  | A:VAL91 | Alkyl | 4.7833 |
| **11498616+**  **3U4K** | **-5.3** | A:SER122:HN | Conventional Hydrogen Bond | 2.21508 |
|  |  | A:ARG125:HE | Conventional Hydrogen Bond | 2.32236 |
|  |  | A:ARG125:HH22 | Conventional Hydrogen Bond | 2.11488 |
|  |  | N:UNK1:H | Conventional Hydrogen Bond | 2.27615 |
|  |  | N:UNK1:H | Conventional Hydrogen Bond | 2.19462 |
|  |  | N:UNK1:C | Pi-Sigma | 3.86498 |
|  |  | A:ALA108 | Alkyl | 5.1581 |
|  |  | A:ILE109 | Alkyl | 5.14807 |
|  |  | A:ARG125 | Alkyl | 3.95181 |
|  |  | A:ALA127 | Alkyl | 4.27363 |
|  |  | N:UNK1 | Alkyl | 5.33185 |
|  |  | N:UNK1 | Alkyl | 5.06145 |
|  |  | N:UNK1:C | Alkyl | 4.23991 |
|  |  | N:UNK1:C | Alkyl | 4.14997 |
|  |  | N:UNK1:C | Alkyl | 5.11237 |
|  |  | A:TYR117 | Pi-Alkyl | 5.25702 |
|  |  | A:PHE124 | Pi-Alkyl | 4.59508 |
|  |  | A:TYR155 | Pi-Alkyl | 4.88907 |
| **9007+**  **3U4K** | **-5.2** | N:UNK1:H | Conventional Hydrogen Bond | 2.73817 |
|  |  | A:PHE124 | Pi-Pi Stacked | 4.53182 |
|  |  | N:UNK1:C | Alkyl | 4.80431 |
|  |  | A:TYR117 | Pi-Alkyl | 5.20807 |
|  |  | N:UNK1 | Pi-Alkyl | 5.32813 |
|  |  | N:UNK1 | Pi-Alkyl | 4.6468 |
| **72713582+**  **3U4K** | **-5.2** | N:UNK1:H | Conventional Hydrogen Bond | 2.79308 |
|  |  | A:VAL38 | Alkyl | 5.36138 |
|  |  | A:ALA51 | Alkyl | 4.83718 |
|  |  | A:ALA51 | Alkyl | 4.23076 |
|  |  | N:UNK1:C | Alkyl | 4.61617 |
|  |  | N:UNK1:C | Alkyl | 4.74254 |
|  |  | A:TRP55 | Pi-Alkyl | 5.17205 |
| **6312351+**  **3U4K** | **-5.2** | A:ARG125:HE | Conventional Hydrogen Bond | 2.37692 |
|  |  | N:UNK1:C | Carbon Hydrogen Bond | 3.69726 |
|  |  | A:ARG125:HN | Pi-Donor Hydrogen Bond | 3.0097 |
|  |  | A:ALA127 | Alkyl | 3.88779 |
|  |  | N:UNK1:C | Alkyl | 4.42721 |
|  |  | A:PHE124 | Pi-Alkyl | 5.09507 |
|  |  | N:UNK1 | Pi-Alkyl | 4.54338 |
| **4476324+**  **3U4K** | **-5.2** | N:UNK1:C | Carbon Hydrogen Bond | 3.40001 |
|  |  | A:ARG125:HN | Pi-Donor Hydrogen Bond | 3.02153 |
|  |  | A:PHE124 | Pi-Pi Stacked | 4.43203 |
|  |  | N:UNK1 | Pi-Alkyl | 5.03307 |
|  |  | N:UNK1 | Pi-Alkyl | 5.15683 |
|  |  | N:UNK1 | Pi-Alkyl | 4.6117 |
| **156581630+**  **3U4K** | **-5.2** | A:VAL38 | Alkyl | 4.811 |
|  |  | A:ALA51 | Alkyl | 5.12057 |
|  |  | A:ALA51 | Alkyl | 3.67494 |
|  |  | A:ALA51 | Alkyl | 4.32387 |
|  |  | N:UNK1:C | Alkyl | 4.43326 |
|  |  | N:UNK1:C | Alkyl | 4.87904 |
| **146683985+**  **3U4K** | **-5.2** | A:TYR117:HH | Conventional Hydrogen Bond | 1.99287 |
|  |  | A:THR119:HG1 | Conventional Hydrogen Bond | 2.21068 |
|  |  | N:UNK1:H | Conventional Hydrogen Bond | 1.91634 |
|  |  | N:UNK1:C | Alkyl | 3.94971 |
| **72713582+**  **3U4K** | **-5.1** | N:UNK1:H | Conventional Hydrogen Bond | 2.79308 |
|  |  | A:VAL38 | Alkyl | 5.36138 |
|  |  | A:ALA51 | Alkyl | 4.83718 |
|  |  | A:ALA51 | Alkyl | 4.23076 |
|  |  | N:UNK1:C | Alkyl | 4.61617 |
|  |  | N:UNK1:C | Alkyl | 4.74254 |
|  |  | A:TRP55 | Pi-Alkyl | 5.17205 |
| **5282606+**  **3U4K** | **-5.1** | A:SER75:HN | Conventional Hydrogen Bond | 2.70329 |
|  |  | A:TYR155:HH | Conventional Hydrogen Bond | 2.67721 |
|  |  | N:UNK1:H | Conventional Hydrogen Bond | 2.461 |
|  |  | N:UNK1:C | Alkyl | 4.21822 |
|  |  | N:UNK1:C | Alkyl | 5.04813 |
|  |  | N:UNK1:C | Alkyl | 4.62061 |
|  |  | N:UNK1 | Alkyl | 4.61834 |
|  |  | N:UNK1 | Alkyl | 5.2994 |
|  |  | N:UNK1 | Alkyl | 5.33877 |
|  |  | N:UNK1:C | Alkyl | 3.73412 |
|  |  | N:UNK1:C | Alkyl | 4.73682 |
|  |  | N:UNK1:C | Alkyl | 3.70506 |
|  |  | A:TYR117 | Pi-Alkyl | 5.20742 |
|  |  | A:PHE124 | Pi-Alkyl | 4.06528 |
| **4696+**  **3U4K** | **-5.1** | A:ARG105:HH12 | Conventional Hydrogen Bond | 2.12728 |
|  |  | A:TYR113:HH | Conventional Hydrogen Bond | 2.59412 |
|  |  | A:ARG105:CD | Carbon Hydrogen Bond | 3.02557 |
|  |  | N:UNK1 | Pi-Pi Stacked | 4.44139 |
|  |  | A:ALA77 | Alkyl | 4.05896 |
|  |  | A:ARG105 | Alkyl | 5.2319 |
|  |  | A:TYR113 | Pi-Alkyl | 5.08278 |
|  |  | N:UNK1 | Pi-Alkyl | 4.02275 |
|  |  | N:UNK1 | Pi-Alkyl | 4.42373 |
| **44254797+**  **3U4K** | **-5.1** | A:TYR117:HH | Conventional Hydrogen Bond | 2.3099 |
|  |  | A:ARG125:HN | Conventional Hydrogen Bond | 2.59092 |
|  |  | A:ARG125:HH22 | Conventional Hydrogen Bond | 2.42199 |
|  |  | N:UNK1:H | Conventional Hydrogen Bond | 2.54566 |
|  |  | A:THR119:CG2 | Pi-Sigma | 3.78714 |
|  |  | A:PHE124 | Pi-Alkyl | 5.38784 |
| **156581631+**  **3U4K** | **-5.1** | A:ARG125:HN | Conventional Hydrogen Bond | 2.93414 |
|  |  | N:UNK1:H | Conventional Hydrogen Bond | 2.42259 |
|  |  | N:UNK1:H | Conventional Hydrogen Bond | 1.79744 |
|  |  | A:ALA108 | Alkyl | 4.2063 |
|  |  | A:ALA127 | Alkyl | 3.88901 |
|  |  | N:UNK1:C | Alkyl | 5.17311 |
|  |  | A:PHE124 | Pi-Alkyl | 4.54047 |
| **156581251+**  **3U4K** | **-5.1** | A:GLY36:HN3 | Conventional Hydrogen Bond | 2.63849 |
|  |  | A:GLY36:HN2 | Conventional Hydrogen Bond | 2.69249 |
|  |  | N:UNK1:C | Carbon Hydrogen Bond | 3.27578 |
|  |  | A:ALA51 | Alkyl | 4.13968 |
|  |  | N:UNK1:C | Alkyl | 4.96544 |
|  |  | N:UNK1:C | Alkyl | 5.42376 |
|  |  | N:UNK1:C | Alkyl | 4.62184 |
| **139591336+**  **3U4K** | **-5.1** | A:THR142:HN | Conventional Hydrogen Bond | 2.0852 |
|  |  | N:UNK1:H | Conventional Hydrogen Bond | 2.1824 |
|  |  | N:UNK1:H | Conventional Hydrogen Bond | 2.41765 |
|  |  | N:UNK1:C | Carbon Hydrogen Bond | 3.55204 |
|  |  | N:UNK1:C | Alkyl | 4.2819 |
| **13942399+**  **3U4K** | **-5.1** | A:THR52:HN | Conventional Hydrogen Bond | 2.63925 |
|  |  | N:UNK1:H | Conventional Hydrogen Bond | 2.53336 |
|  |  | A:VAL38:CA | Carbon Hydrogen Bond | 3.60701 |
|  |  | A:ILE135:CD | Pi-Sigma | 3.97531 |
|  |  | N:UNK1 | Pi-Alkyl | 4.73369 |
|  |  | N:UNK1 | Pi-Alkyl | 5.12713 |
| **10147101+**  **3U4K** | **-5.1** | A:SER123:HN | Conventional Hydrogen Bond | 2.26074 |
|  |  | A:ARG125:HE | Conventional Hydrogen Bond | 2.88814 |
|  |  | A:ARG125:HH22 | Conventional Hydrogen Bond | 2.53707 |
|  |  | N:UNK1:H | Conventional Hydrogen Bond | 2.2976 |
|  |  | N:UNK1:H | Conventional Hydrogen Bond | 2.49352 |
|  |  | N:UNK1:H | Conventional Hydrogen Bond | 1.9407 |
|  |  | A:GLY121:CA | Carbon Hydrogen Bond | 3.30538 |
|  |  | A:ALA108 | Alkyl | 4.77776 |
|  |  | A:ALA108 | Alkyl | 5.274 |
|  |  | A:ARG125 | Alkyl | 5.06556 |
|  |  | A:ALA127 | Alkyl | 4.21064 |
|  |  | A:ALA127 | Alkyl | 5.33903 |
|  |  | N:UNK1:C | Alkyl | 4.04037 |
|  |  | N:UNK1:C | Alkyl | 4.6348 |
|  |  | N:UNK1:C | Alkyl | 3.76076 |
|  |  | A:PHE124 | Pi-Alkyl | 4.04308 |
| **6312351+**  **3U4K** | **-5** | A:ARG125:HE | Conventional Hydrogen Bond | 2.37692 |
|  |  | N:UNK1:C | Carbon Hydrogen Bond | 3.69726 |
|  |  | A:ARG125:HN | Pi-Donor Hydrogen Bond | 3.0097 |
|  |  | A:ALA127 | Alkyl | 3.88779 |
|  |  | N:UNK1:C | Alkyl | 4.42721 |
|  |  | A:PHE124 | Pi-Alkyl | 5.09507 |
|  |  | N:UNK1 | Pi-Alkyl | 4.54338 |
| **31284+**  **3U4K** | **-5** | N:UNK1:C | Pi-Sigma | 3.92705 |
|  |  | A:ARG125 | Alkyl | 4.78331 |
|  |  | A:ALA127 | Alkyl | 3.95383 |
|  |  | N:UNK1:C | Alkyl | 4.29831 |
|  |  | N:UNK1:C | Alkyl | 4.06907 |
|  |  | N:UNK1:C | Alkyl | 4.78113 |
|  |  | A:PHE124 | Pi-Alkyl | 4.94007 |
| **31284+**  **3U4K** | **-5** | N:UNK1:C | Pi-Sigma | 3.92705 |
|  |  | A:ARG125 | Alkyl | 4.78331 |
|  |  | A:ALA127 | Alkyl | 3.95383 |
|  |  | N:UNK1:C | Alkyl | 4.29831 |
|  |  | N:UNK1:C | Alkyl | 4.06907 |
|  |  | N:UNK1:C | Alkyl | 4.78113 |
|  |  | A:PHE124 | Pi-Alkyl | 4.94007 |
| **156581630+**  **3U4K** | **-5** | A:VAL38 | Alkyl | 4.811 |
|  |  | A:ALA51 | Alkyl | 5.12057 |
|  |  | A:ALA51 | Alkyl | 3.67494 |
|  |  | A:ALA51 | Alkyl | 4.32387 |
|  |  | N:UNK1:C | Alkyl | 4.43326 |
|  |  | N:UNK1:C | Alkyl | 4.87904 |
| **148214470+**  **3U4K** | **-5** | N:UNK1:C | Alkyl | 4.23147 |
|  |  | A:TYR117 | Pi-Alkyl | 4.95612 |
|  |  | A:PHE124 | Pi-Alkyl | 4.83504 |
| **11767849+**  **3U4K** | **-5** | A:TYR117:HH | Conventional Hydrogen Bond | 2.31303 |
|  |  | N:UNK1:H | Conventional Hydrogen Bond | 2.85938 |
|  |  | N:UNK1:C | Pi-Sigma | 3.94183 |
|  |  | A:ALA77 | Alkyl | 5.05366 |
|  |  | A:ALA77 | Alkyl | 4.26627 |
|  |  | A:ALA108 | Alkyl | 3.79404 |
|  |  | A:ALA127 | Alkyl | 4.80457 |
|  |  | A:ALA127 | Alkyl | 4.66266 |
|  |  | A:ALA127 | Alkyl | 3.73542 |
|  |  | N:UNK1:C | Alkyl | 4.25144 |
|  |  | N:UNK1:C | Alkyl | 4.63395 |
|  |  | N:UNK1 | Alkyl | 4.99832 |
|  |  | A:TYR113 | Pi-Alkyl | 4.62187 |
|  |  | A:TYR117 | Pi-Alkyl | 5.42706 |
|  |  | A:TYR117 | Pi-Alkyl | 5.22781 |
|  |  | A:TYR117 | Pi-Alkyl | 5.10261 |
|  |  | A:PHE124 | Pi-Alkyl | 4.55433 |
| **10038050+**  **3U4K** | **-5** | A:ARG125:HE | Conventional Hydrogen Bond | 2.64406 |
|  |  | A:ARG125:HH22 | Conventional Hydrogen Bond | 2.97229 |
|  |  | N:UNK1:C | Alkyl | 4.94689 |
|  |  | A:TYR117 | Pi-Alkyl | 5.18359 |
| **779+**  **3U4K** | **-4.9** | A:SER129:HG | Conventional Hydrogen Bond | 2.21217 |
|  |  | A:SER129:HG | Conventional Hydrogen Bond | 2.42562 |
|  |  | A:PHE131:HN | Conventional Hydrogen Bond | 2.82257 |
|  |  | N:UNK1:H | Conventional Hydrogen Bond | 2.35701 |
|  |  | N:UNK1:H | Conventional Hydrogen Bond | 2.35506 |
|  |  | N:UNK1:H | Conventional Hydrogen Bond | 2.30565 |
|  |  | N:UNK1:H | Conventional Hydrogen Bond | 2.33211 |
|  |  | N:UNK1:H | Conventional Hydrogen Bond | 2.66635 |
| **6450508+**  **3U4K** | **-4.9** | A:THR119:HG1 | Conventional Hydrogen Bond | 2.05317 |
|  |  | A:ARG125:HE | Conventional Hydrogen Bond | 2.16594 |
|  |  | A:ARG125:HH21 | Conventional Hydrogen Bond | 2.59458 |
|  |  | A:ARG125:HH22 | Conventional Hydrogen Bond | 1.99475 |
|  |  | N:UNK1:H | Conventional Hydrogen Bond | 2.51303 |
|  |  | N:UNK1:H | Conventional Hydrogen Bond | 2.27583 |
|  |  | N:UNK1:H | Conventional Hydrogen Bond | 2.04764 |
|  |  | N:UNK1:H | Conventional Hydrogen Bond | 2.36492 |
|  |  | N:UNK1:C | Carbon Hydrogen Bond | 3.59131 |
|  |  | N:UNK1:C | Carbon Hydrogen Bond | 3.53103 |
|  |  | N:UNK1:C | Alkyl | 4.47138 |
|  |  | A:TYR117 | Pi-Alkyl | 5.23386 |
|  |  | A:PHE124 | Pi-Alkyl | 5.05966 |
|  |  | A:TYR155 | Pi-Alkyl | 5.3224 |
| **155735+**  **3U4K** | **-4.9** | A:THR142:HN | Conventional Hydrogen Bond | 2.04267 |
|  |  | A:CYS178:HN | Conventional Hydrogen Bond | 2.36069 |
|  |  | A:CYS178:HN | Conventional Hydrogen Bond | 1.91916 |
|  |  | N:UNK1:H | Conventional Hydrogen Bond | 2.41021 |
|  |  | N:UNK1 | Pi-Alkyl | 5.18175 |
| **779+**  **3U4K** | **-4.7** | A:SER129:HG | Conventional Hydrogen Bond | 2.21217 |
|  |  | A:SER129:HG | Conventional Hydrogen Bond | 2.42562 |
|  |  | A:PHE131:HN | Conventional Hydrogen Bond | 2.82257 |
|  |  | N:UNK1:H | Conventional Hydrogen Bond | 2.35701 |
|  |  | N:UNK1:H | Conventional Hydrogen Bond | 2.35506 |
|  |  | N:UNK1:H | Conventional Hydrogen Bond | 2.30565 |
|  |  | N:UNK1:H | Conventional Hydrogen Bond | 2.33211 |
|  |  | N:UNK1:H | Conventional Hydrogen Bond | 2.66635 |
| **49780759+**  **3U4K** | **-4.7** | A:THR119:HG1 | Conventional Hydrogen Bond | 2.56557 |
|  |  | A:ARG125:HH11 | Conventional Hydrogen Bond | 2.98807 |
|  |  | N:UNK1:H | Conventional Hydrogen Bond | 2.53471 |
|  |  | N:UNK1:C | Alkyl | 3.83695 |
|  |  | N:UNK1 | Pi-Alkyl | 4.48045 |
|  |  | N:UNK1 | Pi-Alkyl | 5.02977 |
| **1678+**  **3U4K** | **-4.7** | A:ARG105:HH11 | Conventional Hydrogen Bond | 2.17039 |
|  |  | A:ARG105:HH21 | Conventional Hydrogen Bond | 1.88951 |
|  |  | A:ALA127:HN | Conventional Hydrogen Bond | 2.02599 |
|  |  | A:TYR155:HH | Conventional Hydrogen Bond | 2.24865 |
|  |  | A:TYR155:HH | Conventional Hydrogen Bond | 2.42006 |
| **162642186+**  **3U4K** | **-4.6** | A:GLY98:HN | Conventional Hydrogen Bond | 2.32807 |
|  |  | N:UNK1:H | Conventional Hydrogen Bond | 2.34758 |
|  |  | A:VAL85 | Alkyl | 4.56534 |
|  |  | A:ALA88 | Alkyl | 4.00732 |
|  |  | A:ALA88 | Alkyl | 3.90343 |
|  |  | A:VAL91 | Alkyl | 4.35412 |
|  |  | A:VAL91 | Alkyl | 4.26846 |
|  |  | A:VAL91 | Alkyl | 5.29533 |
|  |  | N:UNK1 | Alkyl | 4.85907 |
|  |  | N:UNK1:C | Alkyl | 4.30001 |
| **10147101+**  **3U4K** | **-4.5** | A:SER123:HN | Conventional Hydrogen Bond | 2.26074 |
|  |  | A:ARG125:HE | Conventional Hydrogen Bond | 2.88814 |
|  |  | A:ARG125:HH22 | Conventional Hydrogen Bond | 2.53707 |
|  |  | N:UNK1:H | Conventional Hydrogen Bond | 2.2976 |
|  |  | N:UNK1:H | Conventional Hydrogen Bond | 2.49352 |
|  |  | N:UNK1:H | Conventional Hydrogen Bond | 1.9407 |
|  |  | A:GLY121:CA | Carbon Hydrogen Bond | 3.30538 |
|  |  | A:ALA108 | Alkyl | 4.77776 |
|  |  | A:ALA108 | Alkyl | 5.274 |
|  |  | A:ARG125 | Alkyl | 5.06556 |
|  |  | A:ALA127 | Alkyl | 4.21064 |
|  |  | A:ALA127 | Alkyl | 5.33903 |
|  |  | N:UNK1:C | Alkyl | 4.04037 |
|  |  | N:UNK1:C | Alkyl | 4.6348 |
|  |  | N:UNK1:C | Alkyl | 3.76076 |
|  |  | A:PHE124 | Pi-Alkyl | 4.04308 |
| **6450521+**  **3U4K** | **-4.2** | A:SER177:HG | Conventional Hydrogen Bond | 2.24219 |
|  |  | A:CYS178:HN | Conventional Hydrogen Bond | 2.47224 |
|  |  | N:UNK1:H | Conventional Hydrogen Bond | 2.89685 |
|  |  | N:UNK1:H | Conventional Hydrogen Bond | 2.28263 |
|  |  | N:UNK1:H | Conventional Hydrogen Bond | 2.33559 |
|  |  | N:UNK1:H | Conventional Hydrogen Bond | 2.33336 |
|  |  | N:UNK1:H | Conventional Hydrogen Bond | 2.83339 |
|  |  | N:UNK1:H | Conventional Hydrogen Bond | 2.57177 |
|  |  | N:UNK1:H | Conventional Hydrogen Bond | 2.91473 |
|  |  | A:SER177:CA | Carbon Hydrogen Bond | 3.46005 |
|  |  | N:UNK1:C | Alkyl | 4.23628 |
|  |  | N:UNK1:C | Alkyl | 4.35647 |
|  |  | N:UNK1:C | Alkyl | 4.30345 |
| **16181015+**  **3U4K** | **-4.1** | A:THR142:HN | Conventional Hydrogen Bond | 2.04267 |
|  |  | A:CYS178:HN | Conventional Hydrogen Bond | 2.36069 |
|  |  | A:CYS178:HN | Conventional Hydrogen Bond | 1.91916 |
|  |  | N:UNK1:H | Conventional Hydrogen Bond | 2.41021 |
|  |  | N:UNK1 | Pi-Alkyl | 5.18175 |
